# Implementation of Natural Products and Derivatives in Acute Myeloid Leukemia Management: Current Treatments, Clinical Trials and Future Directions

**DOI:** 10.3390/cancers18020185

**Published:** 2026-01-06

**Authors:** Faten Merhi, Daniel Dauzonne, Brigitte Bauvois

**Affiliations:** 1 Department of Life & Earth Sciences, Faculty of Sciences (I), Lebanese University, Beirut P.O. Box 14-6573, Lebanon; merhi.faten@gmail.com; 2 Institut Curie, Département Recherche, CNRS UMR3666, Inserm U1339, 75005 Paris, France; daniel.dauzonne@orange.fr; 3 Centre de Recherche des Cordeliers, Sorbonne Université, Université Paris Cité, Inserm UMRS 1138, Drug Resistance in Hematological Malignancies Team, 75006 Paris, France

**Keywords:** acute myeloid leukemia, chemotherapeutic drug, natural compound, phytochemical compound, natural product, chemotherapy, clinical research, signaling

## Abstract

Bioactive natural products (NPs) may play a critical role in cancer progression by targeting nucleic acids and a wide array of proteins, including enzymes. Furthermore, a large number of derivatives (NPDs), including semi-synthetic products and pharmacophores from NPs, have been developed to enhance the solubility and stability of NPs. Acute myeloid leukemia (AML) is a poor-prognosis hematologic malignancy characterized by the clonal accumulation in the blood and bone marrow of myeloid progenitors with high proliferative capacity, survival and propagation abilities. A number of potential pathways and targets have been identified for development in AML, and include, but are not limited to, Fms-like tyrosine kinase 3 (FLT3) and isocitrate dehydrogenases resulting from genetic mutations, BCL2 family members, various signaling kinases and histone deacetylases, as well as tumor-associated antigens (such as CD13, CD33, P-gp). By targeting nucleic acids, FLT3 or CD33, several FDA-approved NPs and NPDs (i.e., cytarabine, anthracyclines, midostaurin, melphalan and calicheamicin linked to anti-CD33) are the major agents of upfront treatment of AML. However, the effective treatment of the disease remains challenging, in part due to the heterogeneity of the disease but also to the involvement of the bone marrow microenvironment and the immune system in favoring leukemic stem cell persistence. This review summarizes the current state of the art, and provides a summary of selected NPs/NPDs which are either entering or have been investigated in preclinical and clinical trials, alone or in combination with current chemotherapy. With multifaceted actions, these biomolecules may target all hallmarks of AML, including multidrug resistance and deregulated metabolism.

## Simple Summary

Acute myeloid leukemia (AML) is a clonal hematologic disorder marked by clinical and biological heterogeneity. Natural products (NPs) derived from plants, animals and microorganisms, have been used as healing agents for thousands of years and still today continue to be the most important source of new potential therapeutic agents. To date, several bioactive NPs and semi-synthetic products, such as cytarabine, anthracyclines, midostaurin and melphalan, are currently approved for the treatment of AML. As the treatment of AML remains an ongoing medical challenge, novel NPs and derivatives targeting tumor-related processes have entered preclinical and clinical trials for AML. This review provides a comprehensive summary of NPs and their derivatives approved for the treatment of AML, as well as those which exhibit potential therapeutic abilities for treating AML.

## 1. Introduction

Acute myeloid leukemia (AML) is a clinically and genetically heterogeneous hematopoietic cancer characterized by the clonal expansion and accumulation of immature myeloid precursors in the bone marrow (BM) and blood [1]. Leukemia cells are unable to undergo growth arrest, terminal differentiation and death in response to appropriate environmental stimuli [1]. Standard therapy consists of induction with 7 days of cytarabine plus 3 days of an anthracycline (7+3) followed by consolidation with additional chemotherapy or stem-cell transplantation [1]. Today, the approval of alternative strategies includes a new liposomal formulation of cytarabine and daunorubicin (CPX-351), or the combination of venetoclax (a BH3 mimetic that inhibits the survival function of the B-cell lymphoma-2 (Bcl-2) anti-apoptotic protein) with hypomethylating agents (decitabine, 5-azacytidine) or low-dose of cytarabine [2,3]. Mylotarg (also named gemtuzumab ozogamicin) has been approved in combination with daunorubicin and cytarabine for the treatment of adult patients newly diagnosed with CD33^+^ AML, and young patients with relapsed/refractory (R/R) CD33^+^ AML [3,4]. Other therapeutic targets have been approved for the treatment of AML patients that carry mutations in the isocitrate dehydrogenase (*IDH1*/*IDH2*) and Fms-like tyrosine kinase 3 (*FLT3*) genes [2,5]. They include ivosidenib and enasidenib, which target IDH1 and IDH2 proteins, respectively [5], and the FLT3 multikinase inhibitors sorafenib and midostaurin [6,7]. Most of these therapies are still accompanied by adverse effects or favored mutations associated with drug resistance [5,8]. Therefore, the development of new drugs directed against AML-specific targets is still needed to increase the cure rate in AML patients exhibiting chemoresistance and poor outcomes.

Nature is a major source of natural products (NPs) with a large chemical diversity and a wide variety of biological activities for treating various diseases, including cancer [9]. Bioactive NPs originate from bacterial, fungal, plant and marine animal sources [9,10,11]. To date, a large variety of NPs have been identified and include anthracyclines, polyphenols, alkaloids, organosulfur compounds, terpenes, terpenoids and other bioactive compounds such as peptides, proteins, carbohydrates, phospholipids, etc. [9,10,11]. NP-derivatives (NPDs) include semi-synthetic NPs and synthetic compounds based on pharmacophores from NPs [9,12]. Semi-synthetic NPs are derived from biologically active NPs by incorporating synthetic components in their molecular structure to improve or modify their molecular properties, including stability and activity [12]. A pharmacophore consists of a biologically active part of a molecule serving as a model [9]. NPs and NPDs may play a critical role in cancer progression by interfering with tumor cell proliferation, survival, metastasis and chemoresistance, as well as AML-associated angiogenesis [13,14,15]. Today, most chemotherapeutic NPs and derivatives used in the clinic are directed at specific nucleic acids, particular proteins or oncogenic pathways [13]. Their mechanisms of action include disruption of chromatin structure, regulation of the synthesis of certain DNA repair proteins, modulation of intracellular signaling pathways (involving a large array of kinases, transcription factors, BCL2 proteins, etc.) and inhibition of various cytokine and enzymatic activities [13,16,17].

An excellent NPD example comes from the use of cytarabine in AML therapy; it is a semi-synthetic derivative of spongothymidine, a natural molecule isolated in 1945 from a Caribbean sea sponge, *Cryptotethia crypta*, and endowed with antitumor activity due to its structural similarity to that of nucleic acid thymidines [10]. In 1969, cytarabine was introduced for the treatment of AML, and is still one of the cornerstones in the treatment of this disease [2]. In this review, we present a summary of the applications of NPs and NPDs that have entered the therapeutic armamentarium for AML, and highlight recent developments that are enabling NP/NPD-based drug discovery with promising results in preclinical and clinical trials for the treatment of AML.

## 2. Roles of Biomarkers in AML Pathogenesis and as Potential Treatment Targets of NPs and NPDs

Human AML cells with abnormally high levels of proliferation and survival transmigrate from BM into peripheral blood and extramedullary organs. BM angiogenesis plays a relevant role in the pathophysiology of AML. In addition to DNA-intercalating NPs/NPDs that block DNA replication and synthesis, a number of AML biomarkers have emerged as targets of NPs and NPDs [18,19]. They are briefly summarized in this section.

### 2.1. HDACs

HDACs as epigenetic modulators regulate gene expression by deacetylation of lysine residues on histone and non-histone proteins [20]. Class I and class II HDAC genes are abnormally expressed in AML and closely correlated with prognosis [21]. HDAC inhibitors promote cell differentiation, growth arrest, apoptosis, and autophagy in preclinical AML models, and inhibit AML-associated angiogenesis [20].

### 2.2. Kinases

The Ras/PI3K/AKT/mTOR and Ras/Raf/MEK/ERK (also known as the MAPK/ERK pathway) cross-talk kinase signalings are frequently activated in AML patient blasts [22,23]. Upregulation of these pathways in AML can result from FLT3 and c-Kit mutated tyrosine kinases or Ras GTPases, which are present in 35% to 40% of all AML [22,23,24,25]. Moreover, the PI3K/AKT/mTOR pathway can regulate the expression of anti-apoptotic BCL2 members (Bcl-2, Mcl-1 and Bcl-x_L_) [26,27]. In consequence, cell survival and proliferation pathways dependent on MAPK, PI3K, AKT and mTOR, as well as NF-κB and STAT3, are deregulated in most cases of AML [28,29,30]. Adhesion to BM stromal cells and migration of AML blasts involve the PI3K signaling [31].

### 2.3. Transcription Factors

Constitutive NF-κB activation has been reported in approximately 40% to 70% of AML patients, and ensues in part from recurrent genetic alterations of upstream regulators of its pathway [32]. Notably, NF-κB is constitutively active in CD34^+^ CD38^−^ stem cells (leukemia stem cells/LSCs) from AML patients with French–American–British (FAB) M3/M4/M5 subtypes [33,34]. NF-κB activation is involved in sustaining AML cell proliferation, survival and chemoresistance [30,35]. NF-κB mediates chemoresistance in AML cells, based at least on its ability to stimulate the expression of anti-apoptotic proteins (i.e., Bcl-2, Bcl-x_L_, Mcl-1, XIAP, FLIP), thus enabling AML cells to evade apoptosis and increase proliferation [36,37]. Constitutive STAT3 activation is observed in approximately 50% of newly diagnosed AML, and is associated with adverse prognosis [38]. STAT3 appears essential to the survival of AML LSCs [39]. STAT3 regulates AML cell survival and proliferation by upregulating the expression of its target genes (e.g., c-Myc, cyclin D1, survivin, Bcl-2, Mcl-1) [38,40,41]. STAT3 mediates oxidative phosphorylation (OXPHOS) and glutamine uptake in AML cells and LSCs [42].

### 2.4. Anti-Apoptotic BCL2 Members

Anti-apoptotic BCL2 members (mainly Bcl-2, Mcl-1, Bcl-x_L_) are frequently overexpressed in AML [43,44]. They are critical for the survival of AML cells and LSCs [44] and associated with relapse of AML, and confer chemotherapy resistance in AML [44,45]. The anti-apoptotic proteins bind and sequester the apoptotic effectors Bax and Bak in an inactive form, and thus act to prevent Bax/Bak-driven apoptosis, thereby maintaining cell survival [46]. High levels of Mcl-1 are found in AML blasts with internal tandem duplications (ITD) of FLT3 [47]. FLT3-ITD upregulates Mcl-1 to promote survival of LSCs via constitutive STAT3 and/or STAT5 signaling [47,48].

### 2.5. Tumor-Associated Antigens

Aminopeptidase-N (APN)/CD13 preferentially cleaves small neutral amino acids from the N-terminus of small peptides [49]. In AML, CD13 is strongly expressed on LSCs and blasts in all AML FAB subtypes [50,51,52]. CD13 regulates cell proliferation and survival in AML cells, through the activation of the PI3K/AKT pathway and modulation of Bcl-2, Mcl-1 and Bax proteins [53,54]. Our laboratory showed that transmigration of AML cell lines in a Transwell migration assay was neither blocked by bestatin (APN inhibitor) nor anti-CD13 Abs, thus strongly suggesting that surface CD13 is not required for the migration of AML cells.

CD33 is a member of the sialic acid-binding immunoglobulin-like lectin family that is involved in cell–cell interactions [55]. CD33 full length and three splice variants of CD33 (that lack exon 2) are present in AML cells [56]. CD33 is highly expressed on a subset of LSCs, on leukemic myeloblasts and AML cells with FLT3 mutations, in most AML patients, regardless of age [55,57,58]. In primary AML cells expressing SYK/ZAP70 kinases, ligation of CD33 mediates induction of death and enhances the cytotoxic effects of cytarabine and idarubicin on leukemic cells [55,59,60].

The presence of various CD44 variants, particularly CD44v6-v10, is observed in primary AML and LSCs [61,62]; their high levels correlate with advanced disease and short survival of patients [61], as well as with relapse and chemotherapy resistance [63,64]. CD44 on AML cells acts as a receptor for the natural glycosaminoglycan hyaluronic acid (HA) and matrix metalloproteinase-9 (MMP-9) [65,66]. CD44 engagement regulates AML cell survival and proliferation (through PI3K/AKT/Mcl-1 pathway) [67,68], mediates AML cell infiltration of BM [69], and reduces AML cell repopulation in serial transplantations by eradication of LSCs [55].

The transmembrane transporter P-glycoprotein/P-gp encoded by the *ABCB1* transporter gene (also named multidrug resistance/*MDR* gene) transports numerous drugs, thus preventing the intracellular accumulation of cytotoxic agents [70]. *ABCB1* polymorphisms affect P-gp expression and activity [71]. AML patients, except those with the FAB M5, have a high expression and activity of P-gp [43,72,73]. P-gp is overexpressed in LSCs compared to more differentiated subsets [74]. Overexpression of P-gp is considered to be the primary cause of MDR in patients with AML, often associated with a poor prognosis, and affects the efficacy of treatment and event-free survival [71,72,73,75,76].

Vascular endothelial growth factor-receptor 2 (VEGF-R2) belongs to the tyrosine kinase receptor superfamily, and is the main receptor for VEGF-A (e.g., VEGF_165_) [77]. Enhanced expression of VEGF and VEGF-R2 in BM correlated with enhanced BM angiogenesis [78,79]. AML cells from BM and blood express high levels of VEGF-R2 and VEGF [79,80,81,82,83]. Overexpression of VEGF and VEGF-R2 has been clinically associated with an aggressive clinical course, chemotherapy resistance and poor prognosis in patients with AML [78,84]. By interacting with VEGF-R2, autocrine VEGF-A supports AML cell survival, proliferation and migration through NF-κB, PI3K/AKT, ERK, HSP90 signaling proteins and Bcl-2 and Mcl-1 anti-apoptotic proteins [79,85,86,87,88].

In addition to VEGF-A, VEGF-C is highly expressed by AML blasts [89,90]. AML patients with high VEGF-C levels at diagnosis show poor biological responses [91,92]. A high VEGF-C expression is related to drug resistance in vitro and in vivo [92]. Binding of VEGF-C to its receptors (e.g., VEGF-R2 and VEGF-R3) favors AML cell proliferation and survival through activation of the endothelin-1/cyclo-oxygenase-2 (ET-1/COX-2)/JNK/AP-1 axis, and upregulating Bcl-2 expression [90,91,93,94]. Accordingly, VEGF-A or VEGF-C stimulation protects AML cells from chemotherapy-induced apoptosis via ET-1/COX-2 pathway and anti-apoptotic proteins [86,93,94,95,96]. Moreover, VEGF can upregulate the expression of MMP-2 and MMP-9 in primary AML cells [97].

### 2.6. Soluble Hemoregulators

Blood and BM AML blasts express and release both active and inactive forms of MMP-2 and MMP-9 [81,98,99,100,101]. MMP-2 transcription is mainly regulated by STAT3 [102], while MMP-9 transcription is regulated via NF-κB, AP-1 and SP-1 [103]. Constitutive MMP-2 release is associated with decreased AML cell chemosensitivity [98,99]. Through their enzymatic activities, MMP-2 and MMP-9 likely contribute to the dissemination of AML cells from the BM, and resistance to chemotherapy [104,105,106]. Accordingly, MMP inhibition improves chemotherapy effectiveness in AML models [98,106,107]. In addition, MMP-2 and MMP-9 can be detected as membrane-bound forms on the surface of AML cells [99,107,108]. The binding of MMP-9 to the integrins αLβ2 and αMβ2 on AML cell lines induces their migration [104].

Tumor necrosis factor-α (TNF-α) exists in two active forms, e.g., the transmembrane form (tmTNF-α) and the secreted form (sTNF-α). Secreted TNF-α is released from tmTNF-α through proteolytic cleavage by ADAM17 (a disintegrin and metalloprotease 17) [109], which is overexpressed on AML cells [52]. Both forms of TNF-α are expressed by AML cells [110,111,112]. A high level of tmTNF-α is correlated with a higher percentage of CD34^+^ AML cells, extramedullary infiltration, and an adverse risk group [111]. TNF-α enhances AML cell survival and drug resistance by inducing NF-κB activation [113,114], and upregulation of Bcl-2, Mcl-1 and Bcl-x_L_ [36,114]. Alternatively, TNF-α without NF-κB activation can enhance AML cell survival and drug resistance by activating a JNK/AP-1/Mcl-1 signaling [112] or upregulating heme oxygenase-1 expression [115]. NF-κB signaling comprises two independent but interlinked signaling pathways [116]: the canonical or classical pathway mediated by the action of the RelA/p50 subunits, and the non-canonical or alternative pathway that is dependent on activation of the RelB subunit associated with p50 or p52 [116]. The canonical pathway of NF-κB is constitutively active in AML cells [33], while the FLT3/ITD activates the noncanonical NF-κB signaling pathway [117].

## 3. Current Treatments of AML Using NPs and NPDs

**Figure 1:**
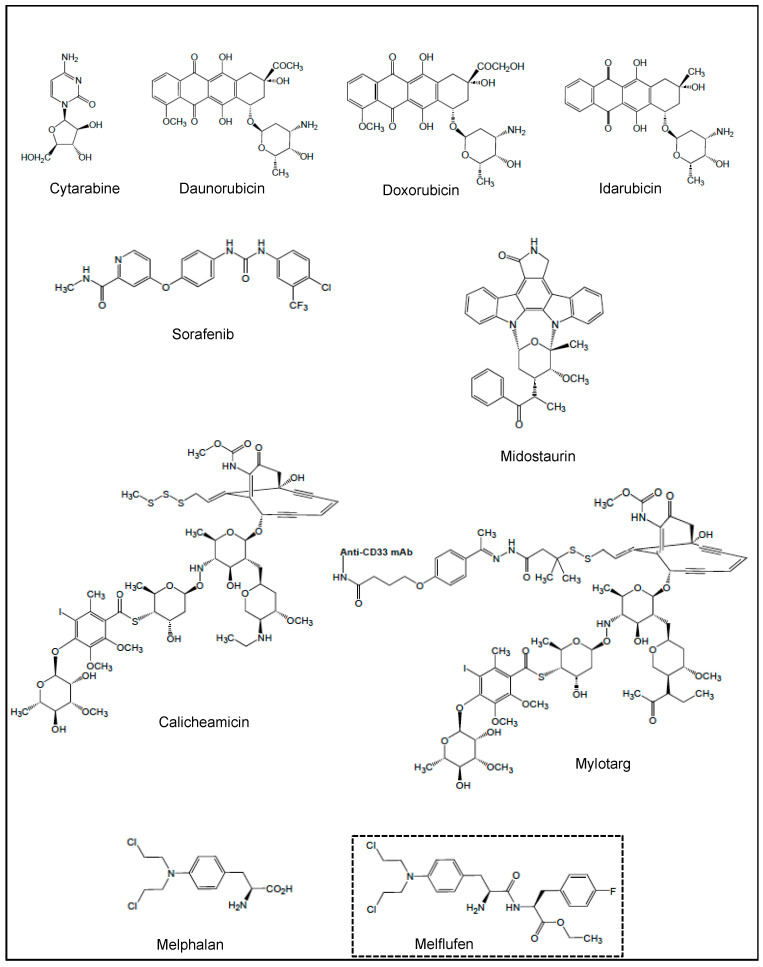
Chemical structures of NPs and NPDs used in current treatments of AML, and melflufen, a melphalan derivative.

To date, the NPs and NPDs currently approved by the US Food and Drug Administration (FDA) for the treatment of AML include the cytosine derivative cytarabine, the anthracyclines daunorubicin, doxorubicin and idarubicin, the FLT3 inhibitors sorafenib and midostaurin, calicheamicin conjugated to anti-CD33 (mylotarg) and melphalan (Figure 1). In addition, clinical and preclinical trials which are either entering or have evaluated these agents in combination with other drugs are summarized in this section.

### 3.1. Cytarabine, Anthracyclines and CPX-351

Cytarabine (also known as cytosine arabinoside, ara-C) is a semi-synthetic derivative of spongothymidine, a natural molecule isolated from a sponge *Cryptotehia crypta*; it is an antimetabolite that interferes with DNA synthesis, and inhibits DNA and RNA polymerases and nucleotide reductases needed for DNA synthesis [118]. Anthracyclines are DNA-intercalating agents that block DNA replication (by disrupting the topoisomerase-II-mediated DNA repair) and its subsequent synthesis, therefore causing cell growth arrest and cell death [118]. Daunorubicin (Figure 1), produced by the fermentation of *Streptomyces* strains, was the first anthracycline discovered in 1963, and received its first marketing authorization in France in 1968 and in the United States in 1974. Daunorubicin was initially used for AML treatment until its semi-synthetic derivatives doxorubicin and idarubicin (Figure 1) were proven more effective by generating free radicals that cause cellular damage [118,119]. The combination and schedule of these molecules for AML, widely known as the “7+3” regimen, remains the backbone of AML standard therapy [120]. Then, the approval of alternative strategies includes a liposomal formulation of cytarabine and daunorubicin (CPX-351) for patients previously exposed to chemotherapy or radiation therapy, or the combination of a low dose of cytarabine with venetoclax [121]. Several phase I/II trials have started to investigate the combination of CPX-351 with other drugs (such as venetoclax, mylotarg, FLT3 inhibitors, etc.) [122,123].

### 3.2. Sorafenib and Midostaurin

Sorafenib (Figure 1) was built from NP pharmacophores, i.e., a synthetic nicotinamide and a diphenylurea derivative; nicotinamide is found in certain meat and vegetables, while N,N′-diphenyl urea was first isolated from coconut milk in 1955. Sorafenib inhibits various intracellular and cell surface kinases, including FLT3, Raf and VEGF-Rs [124]. Sorafenib, in monotherapy or in combination with conventional chemotherapy, has been used in various settings in AML, including front-line R/R disease, including post-allograft failures and post-transplant maintenance therapy [7,125]. Among new developed FLT3 inhibitors with greater potency [126], midostaurin (Figure 1) is a synthetic derivative of staurosporine, an alkaloid originally isolated from the bacterium *Streptomyces staurosporeus*. In 2017, midostaurin was approved by the FDA for the treatment of adult patients with newly diagnosed FLT3-mutated AML, in combination with standard cytarabine and daunorubicin induction, and cytarabine consolidation [127,128]. The addition of midostaurin maintenance therapy following allogeneic hematopoietic cell transplantation (alloHCT) may provide clinical benefit in some patients with FLT3-ITD AML [129]. A recent phase I trial of midostaurin and mylotarg used in combination with standard cytarabine and daunorubicin induction has started in patients with newly diagnosed FLT3-mutated AML (NCT03900949, period 2019–2025) [130]. An active phase I/II trial (NCT04982354, period 2022–2030) has been designed to identify the effect of midostaurin combined with CPX-351 as induction and consolidation therapy for patients with high-risk FLT3 mutated AML, and subsequent alloHCT.

### 3.3. Calicheamicin and Mylotarg

Calicheamicin (Figure 1) is a naturally occurring hydrophobic enediyne that was first isolated from the actinomycete *Micromonospora echinospora calichensis*. It is a potent DNA-binding cytotoxic agent [131]. To enhance its therapeutic value, a derivative of calicheamicin (i.e., calicheamicin 1,2-dimethyl hydrazine dichloride) has been conjugated with an anti-CD33 mAb, giving an antibody–drug conjugate (ADC) (mylotarg, also known as gemtuzumab ozogamicin) (Figure 1) [131]. After binding to CD33 on the surface of AML cells, mylotarg enters the cell via receptor-mediated endocytosis and releases the lethal drug. Despite the clinical efficacy in R/R AML [132], mylotarg was withdrawn from the market in 2010 due to increased early deaths seen in newly diagnosed AML patients receiving mylotarg plus intensive chemotherapy [4]. In 2017, the FDA regranted approval for mylotarg in combination with daunorubicin and cytarabine for the treatment of adult patients newly diagnosed with CD33^+^ AML, and young patients with R/R CD33^+^ AML [3,4]. Several clinical trials have evaluated mylotarg in combination with various therapeutic drugs (i.e., busulfan, cyclophosphamide, azacitidine, all-trans retinoic acid (ATRA), venetoclax, glasdegib (an inhibitor of the Hedgehog pathway), zosuquidar, midostaurin and gilteritinib (a new FLT3 inhibitor) in various AML populations [131,133,134,135,136,137,138,139]. An active phase III in patients with newly diagnosed AML with or without FLT3 mutations (NCT04293562, period 2020–2027)) studies the combination of mylotarg with CPX-351 and/or gilterinitib.

### 3.4. Melphalan and Melflufen

Melphalan (Figure 1) was synthesized from L-phenylalanine in 1954 [140]. Phenylalanine was first discovered in yellow lupine (*Lupinus luteus*), and is mainly found in meat, milk, oil seeds and legumes. Melphalan is an alkylating agent, which interferes with the synthesis of DNA and RNA. Patients with R/R AML have a particularly poor outcome, and alloHCT is the only potential curative option for them. To improve alloHCT results in this setting, patients have to receive high-dose myeloablative chemotherapy before alloHCT. The IDA-FLAG (idarubicin-fludarabine/cytarabine (Ara C)/G-CSF) chemotherapy is based on its cytoreductive properties in R/R AML [141]. Furthermore, the IDA-FLAG plus high-dose melphalan-based sequential alloHCT was selected due to its capability in contributing to a high rate of complete response and a low relapse incidence [142,143].

Among developed derivatives of melphalan, designed to increase its activity or selectivity [144], a prodrug of melphalan, the melphalan flufenamide abbreviated melflufen (L-melphalanyl p-L-fluorophenylalanine ethyl ester hydrochloride) was synthesized [145] (Figure 1). With the insertion of a simple peptide bond, the activity of melflufen is directed to APN/CD13-expressing tumor cells. Under the enzymatic action of APN, the peptide bond of melflufen is cleaved, resulting in the release of melphalan, which, due to its hydrophilicity, is trapped inside the cell, and interacts with DNA [145]. Melflufen shows significant anti-leukemia activity in primary AML cells and murine AML xenografts, and inhibits angiogenesis in different preclinical AML models in vitro and in vivo [144,146]. Regardless of FAB subtype, primary AML cells that are resistant to venetoclax exhibit in vitro good sensitivity to melflufen [147].

## 4. Clinical Trials of AML Using NPs and NPDs

**Figure 2:**
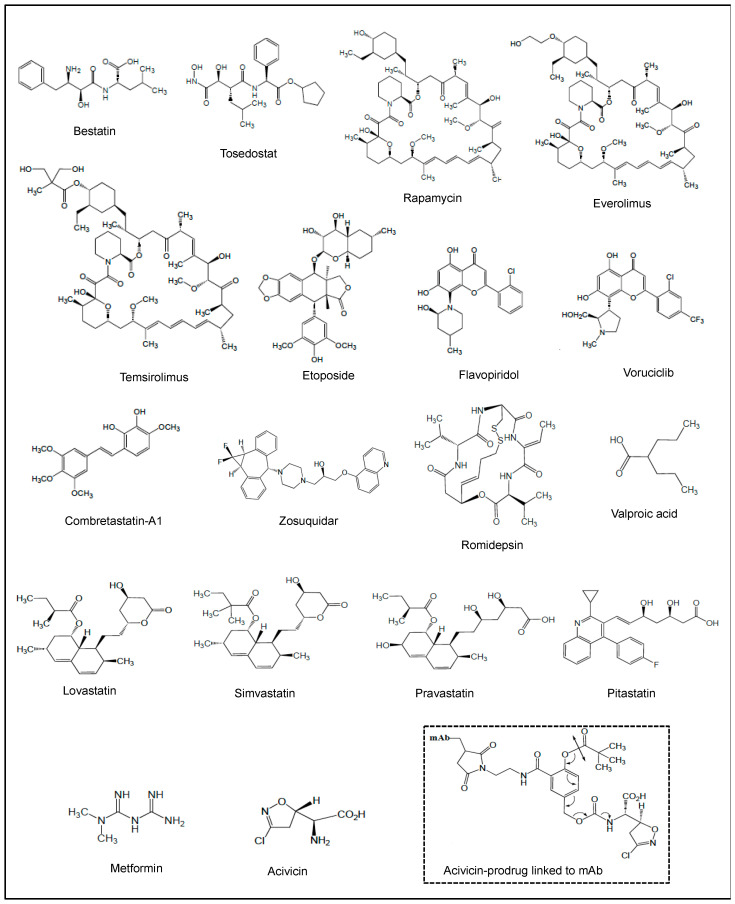
Chemical structures of NPs and NPDs investigated in clinical trials of AML, and acivicin-prodrug linked to mAb.

In this section, we discuss the roles of representative NPs and NPDs which have been involved in AML therapeutic purposes. The structures of these molecules are shown in Figure 2, and Table 1 summarizes their actions in clinical trials of AML. These studies have given hints in what ways to steer more effective treatment regimens.

### 4.1. Bestatin and Tosedostat

Bestatin (also known as Ubenimex) is a Phe-Leu-dipeptide (Figure 2) first isolated from *Streptomyces olivoreticuli*. Bestatin inhibits the proteolytic activity of APN/CD13 in AML cell lines and primary AML blasts [53,54]. Interaction of APN/CD13 with high-dose bestatin induced apoptosis in AML cell lines through the caspase-3, MAPK and glycogen-synthase kinase (GSK)-3β pathways [53]. The combination of cytarabine and bestatin had a synergistic impact in inducing apoptosis of AML cell lines, with Bax expression increased and the expression of Bcl-2, PI3K and AKT decreased [148].

While bestatin has been used for over 35 years in Japan, it has not been approved for any indication in the United States or Europe. In first clinical trials, therapeutic efficacy of bestatin was demonstrated by a prolongation of survival of patients with AML [149,150,151], and in promoting graft versus leukemia effects in patients following alloHCT [152] (Table 1). The effective and safe regimen of cytarabine, aclarubicin (anthracycline produced by *Streptomyces galilaeus*) and G-CSF (CAG) was widely used in China and Japan for the treatment of patients with new or R/R AML [153]. Bestatin had limited cytotoxicity, and was then used at low doses in combination with the CAG regimen to enhance its antitumor effects [154,155] (Table 1).

In order to overcome the cytotoxic limitation of bestatin, several bestatin derivatives or analogues, as well as bestatin-conjugates, have been developed [156,157,158]. Among them, tosedostat (CHR-2797) (Figure 2) is a synthetic aminopeptidase inhibitor related to bestatin [159]. Tosedostat has been evaluated in clinical trials for the treatment of AML (Table 1). In phase I/II trials, tosedostat showed acceptable toxicity and encouraging efficacy in R/R AML [160,161] (Table 1). When combined with low-dose cytarabine, decitabine or azacitidine, tosedostat was not associated with major toxic effects in newly diagnosed patients with AML [162], as well as in elderly patients with AML [163] (Table 1). A randomized phase II multicenter study (HOVON 103) confirmed the safety of the combination of tosedostat with standard chemotherapy (Table 1). However, phase II randomized studies with a large AML sample size demonstrated that addition of tosedostat to standard chemotherapy or low-dose cytarabine negatively affected the therapeutic outcome of AML patients due to more infection-related deaths [164,165] (Table 1). Using the pharmacophore fusion strategy, a new bestatin–fluorouracil conjugate was developed and provided a basis for the design of bestatin-based conjugates or hybrids [157]. Furthermore, two bestatin–vorinostat hybrids have been developed as potent APN/CD13 and HDAC dual inhibitors, which exhibit antitumor activity in vitro [158,166].

**Table 1:** Selected clinical trials using NPs and NPDs in AML.

NP/NPD	Class/Activity	Clinical Study
Bestatin (Ubenimex) (NP)	Phe-Leu-dipeptide/APN inhibitor	Phase I untreated AML [149,150,151]
		Phase I AML following alloHCT [152]
		Phase I untreated and R/R AML, in combination with cytarabine/aclarubicin/G-CSF [154,155]
Tosedostat (NPD)	Bestatin peptidomimetic/APN inhibitor	Phase I/II older or relapsed AML (CHR-2797-002) [160]
		Phase II R/R AML (OPAL, NCT00780598) [161]
		Phase II newly diagnosed AML (NCT01567059), in combination with cytarabine or decitabine [162]
		Phase I/II in elderly R/R AML patients (NCT01636609), in combination with cytarabine or azacitidine [163]
		Phase II very poor risk AML (except FAB M3) (HOVON 103, NL-OMON22002), in combination with daunorubicin/cytarabine
		Phase II in elderly AML patients (Leukemia Working Group of the HOVON/SAKK Cooperative Groups) [164]
		Phase II in untreated elderly patients (ISRCTN40571019), in combination with low-dose cytarabine [165]
Rapamycin (NP)	Macrocyclic lactone/mTOR inhibitor	Phase I R/R AML or untreated secondary AML, in combination with mitoxantrone/etoposide/cytarabine [167]
		Phase I R/R and untreated high-risk AML (NCT00780104)
		Phase II (NCT01184898), in combination with mitoxantrone/etoposide/cytarabine [168]
		Phase I newly diagnosed AML (NCT01822015), in combination with cytarabine/daunorubucin [169]
Temsirolimus (NPD)	Macrocyclic lactone/mTOR inhibitor	Phase II for elderly AML patients (NCT007755903), in combination with clofarabine [170]
Everolimus (NPD)	Macrocyclic lactone/mTOR inhibitor	Phase Ib first relapse AML (NCT01074086), in combination with cytarabine/daunorubicin [171]
		Phase Ib/II R/R AML (ACTRN12610001031055), in combination with cytarabine [172]
		Phase I AML (UK NCRI AML17 trial), in combination with consolidation high-dose cytarabine [173,174]
Etoposide (NPD)	Glycoside/ topoisomerase II inhibitor	Phase I R/R AML, in combination with cytarabine [175]
		Phase I R/R AML, in combination with fludarabine/cytarabine [176]
		Phase I R/R AML, in combination with mitoxantrone/cytarabine [177]
		Phase I/II untreated and R/R AML (NCT02921061), in combination with mitoxantrone/cytarabine following priming with decitabine [178]
		Phase I R/R AML, in combination with fludarabine/cytarabine/G-CSF [179]
		Phase I p53 mutated AML (ChiCTR-INR-16009337) in combination with decitabine [180,181]
Flavopiridol (NPD)	Flavone/kinase inhibitor	Phase I poor-risk AML (NCT00470197), in combination with cytarabine/mitoxantrone [182]
		Phase I AML (NCT00278330), in combination with vorinostat [183]
		Phase II poor-risk AML (NCT00795002), in combination with cytarabine/mitoxantrone [184,185,186]
		Phase I newly diagnosed high-risk AML (NCT03298984), Phase II newly diagnosed AML (NCT00407966), Phase I R/R AML (NCT03563560), in combination with cytarabine/mitoxantrone or cytarabine/daunorubicin [187,188,189]
		Phase II R/R AML (NCT00634244), in combination with cytarabine/mitoxantrone or sirolimus/mitoxantrone/etoposide [190]
		Phase II Mcl-1-dependent-R/R AML (NCT02520011), in combination with cytarabine/mitoxantrone [191]
		Phase II AML (NCT01349972), in combination with cytarabine/daunorubicin/mitoxantrone
		Phase I AML (NCT00064285), in combination with imatinib
		Phase Ib R/R AML (NCT03441555), in combination with venetoclax [192]
		Phase II R/R AML (NCT03969420, active trial), in combination with venetoclax
Voruciclib (NPD)	Flavone/kinase inhibitor	Phase I R/R AML (NCT03547115), in combination with venetoclax [193]
Combretastatin-A1 (NP)	Stilbene/DNA-damaging agent	Phase I/Ia R/R AML (NCT01085656) [194,195]
		Phase Ib R/R AML (NCT02576301), in combination with cytarabine [196,197]
Zosuquidar (NPD)	Quinoline alkaloid/P-gp inhibitor	Phase I newly and relapsed AML, in combination with cytarabine/daunorubicin [198]
		Phase I in untreated AML, in combination with cytarabine/daunorubicin [199]
		Phase III in untreated elderly AML patients (NCT00046930), in combination with cytarabine/daunorubicin [200]
		Phase I/II in untreated elderly AML patients (NCT00129168), in combination with cytarabine/daunorubicin [201]
		Phase I/II untreated AML (NCT00233909), in combination with mylotarg [137]
Romidepsin (NP)	Bicyclic peptide/HDAC inhibitor	Phase I newly and relapsed AML (approval from The Ohio State University Institutional Review Board) [202]
		Phase I/II R/R AML (NCT00062075), in combination with azacytidine [203,204]
Valproic acid (NPD)	Fatty acid/HDAC inhibitor	Phase I/II/III AML, in combination with ATRA [205,206,207,208]
		Phase I/II AML (NCT00414310), Phase I R/R AML (NCT00079378), Phase I AML ((NCI U01 CA 76576-05)
		Phase II AML (NCT01305499), in combination with decitabine [209,210,211]
		Phase I/II relapsed AML after alloHCT (NCT01369368)
		Phase II AML/MDS (NCT00382590), in combination with 5-azacytidine versus low-dose cytarabine
		Phase II AML, in combination with low-dose cytarabine [212]
		Phase II AML, in combination with ATRA and 5-azacytidine (NCT00326170) [213], (NCT00339196) [214]
		Phase I AML (NCT00175812), in combination with ATRA and theophylline [215,216]
		Phase I/II AML (NCT00995332), in combination with ATRA and low-dose cytarabine [217]
		Phase I/II AML (NCT00867672), in combination with ATRA and decitabine [218,219]
		Phase III in old AML patients (NCT00151255), with standard induction (cytarabine/idarubicin), and consolidation (cytarabine/mitoxantrone) [220]
		Phase II high risk AML/MDS (NCT02124174), in combination with 5-azacytidine as maintenance therapy post alloHCT [221]
Lovastatin (NP)	Statin/lipid-lowering drug	Phase I/II R/R AML (NCT NCT00583102), with high-dose cytarabine [222]
Pravastatin (NPD)	Statin/lipid-lowering drug	Phase I AML (MDACC IRB, protocol no. 2004-0185), in combination with idarubicin and high-dose cytarabine [223]
		Phase II R/R AML (NCT00840177), in combination with idarubicin and cytarabine [224]
		Phase I/II R/R AML (NCT01342887), in combination with etoposide, cyclosporine and mitoxantrone [225]
Pitavastatin (NPD)	Statin/lipid-lowering drug	Phase I AML (NCT04512105), in combination with venetoclax [226]
Metformin (NP)	Biguanide/mitochondrial GPDH inhibitor	Retrospective studies with diabetic AML patients [227,228] Phase I R/R AML (NCT01849276), in combination with cytarabine
Acivicin (NP)	Glutamine analog/γ-GT inhibitor	Phase I/II R/R AML [229]

AlloHCT, allogeneic hematopoietic cell transplantation; APN, aminopeptidase-N; ATRA, all-trans retinoic acid; γ-GT, gamma-glutamyl transpeptidase; G-CSF, granulocyte-colony stimulating factor; HDAC, histone deacetylase; NP, natural product; NPD, natural product derivative; R/R, relapsed/refractory.

### 4.2. Rapamycin, Temsirolimus and Everolimus

Rapamycin (also known as sirolimus) (Figure 2) was first isolated from a filamentous bacterium *Streptomyces hygroscopicus*. Its mechanism of action uncovers the inhibition of mTOR kinase that regulates mRNA translation and protein synthesis, an essential step in cell growth, proliferation and survival [230]. Rapamycin markedly impaired the clonogenic properties of fresh AML cells while sparing normal hematopoietic progenitors [231]. In vitro studies showed that rapamycin inhibited the proliferation of AML cells and induced apoptosis associated with the inhibition of the mTOR, 4E-BP1 and p70S6K pathways, and VEGF expression [231,232,233,234].

Initial clinical studies showed that rapamycin was well-tolerated in patients with refractory AML [235], and confirmed the feasibility of combining rapamycin and induction chemotherapy in R/R and untreated high-risk AML [167,168] (Table 1). Recently, a phase I trial of rapamycin with conventional “7+3” chemotherapy in patients with newly diagnosed AML showed it was a well-tolerated and safe regimen [169] (Table 1). Furthermore, two new rapamycin analogues were developed, e.g., temsirolimus, which is a soluble ester of rapamycin [236], and everolimus, which is a hydroxyethyl ether of rapamycin [237] (Figure 2). Both derivatives inhibit the growth of AML cell lines and VEGF expression [232]. Furthermore, they inhibit more efficiently mTOR signaling and AKT activation in primary AML cells [238]. They enhance chemotherapy response in bulk and AML stem populations [231,239]. The combination of temsirolimus with clofarabine (a nucleoside analogue) displays synergistic cytotoxic effects against AML cells, translated by growth arrest, apoptosis and autophagy (associated with inactivation of AKT and mTORC1 downstream targets) [240]. Three clinical trials provided evidence that temsirolimus and everolimus can enhance chemosensitivity in relapsed AML patients [170,171,172] (Table 1). However, one clinical trial performed on a large cohort of AML patients suggested that the addition of everolimus to consolidation therapy provided no benefit and was terminated due to immunosuppressive effects of everolimus leading to infections [173,174] (Table 1).

### 4.3. Etoposide

Etoposide (also known as VP-16) (Figure 2) is a semi-synthetic glycoside derivative of podophyllotoxin which is a non-alkaloid compound extracted from the rhizomes and roots of certain *Podophyllum* species. Etoposide inhibits topoisomerase II, which is responsible for rewinding broken DNA strands [241]. The in vitro pro-apoptotic effect of etoposide related to caspase-3-mediated Bcl-2 cleavage and topoisomerase II inhibition was demonstrated in AML cell lines and primary AML blasts [242,243,244,245,246,247]. Synergism with doxorubicin, rapamycin and other drugs has been demonstrated in vitro and in AML murine xenografts [239].

In initial clinical trials, etoposide, as a single agent, was active in R/R AML, and gave complete response rates of 10% to 25% [248]. Etoposide was safely combined with cytarabine, anthracyclines (daunorubicin, doxorubicin, idarubicin), mitoxantronerapamycin, etc., inducing remission rates of 20% to 60% in R/R AML [249]. Following clinical studies showed encouraging results, with a high percentage of new and R/R AML patients achieving complete remission [175,176] (Table 1). The combination of mitoxantrone, etoposide and cytarabine can induce deep complete remission and is an effective bridge therapy to alloHCT in R/R AML patients [177] (Table 1). Moreover, the fludarabine, high-dose cytarabine, G-CSF and etoposide regimen showed a moderate toxicity profile in heavily pretreated R/R AML patients enabling stem cell consolidation [179] (Table 1). However, a phase I/II study of mitoxantrone, etoposide and cytarabine following priming with decitabine (an analogue of cytarabine) in patients with R/R AML showed no evidence that this combination was substantially better than other cytarabine-based regimens currently used for R/R AML [178] (Table 1). More recently, combinations of decitabine and etoposide regimens appeared relatively safe and tolerable in elderly patients with p53 mutated AML [180,181] (Table 1).

### 4.4. Flavopiridol and Voruciclib

Flavopiridol (also known as alvocidib) (Figure 2) is a semi-synthetic flavone derived from rohitukine present in the Indian tree *Dysoxylum binectariferum*. Initially described as a potent inhibitor of multi-serine threonine cyclin-dependent kinases (CDKs) by binding to the ATP-binding domain of CDKs [250], flavopiridol lacks absolute specificity since it can inhibit the activity of other kinases (including AKT, MAPKS, JNK, PKC, and IκBα kinase) and MMP-9, as well as the expression of anti-apoptotic BCL2 proteins [250,251]. Flavopiridol induced apoptosis in various AML cell lines and BM AML blasts, associated with downregulation of Mcl-1 and Bcl-2 [252,253,254,255,256,257].

Flavopiridol was the first CDK inhibitor to enter clinical trials for the treatment of solid tumors. An initial phase I clinical study of flavopiridol in R/R AML patients showed a modest anti-leukemic activity [258,259]. Correlative pharmacodynamic studies in AML BM blasts demonstrate that flavopiridol induced suppression of tumor-associated genes (encoding Mcl-1, Bcl-2, VEGF, E2F1, STAT3, cyclin D1 and RNA polymerase II), and increased the cytotoxic effects of cytarabine [182,258,260,261,262]. In phase I/II clinical trials, flavopiridol showed a significant clinical activity in AML (R/R and newly diagnosed non-favorable risk AML patients), when combined in various sequential chemotherapy regimens (daunorubicin, cytarabine, imatinib, mitoxantrone, vorinostat, sirolimus, etoposide) [182,183,184,185,186,187,188,189,190,191,250] (Table 1). The best rate of complete remission or remission with incomplete hematologic recovery was seen with flavopiridol combined with the “7+3” conventional therapy [187,188,189]. While a phase Ib of flavopiridol combined with venetoclax for R/R AML showed no increase in efficacy across all cohorts compared to what was previously observed with each agent alone [192] (Table 1), an ongoing phase II R/R AML is still evaluating, in combination, flavopiridol plus venetoclax in R/R AML (Table 1). New synthetic derivatives of flavopiridol, including voruciclib (Figure 2), exhibited improved CDK-inhibitory activity, efficiently induced apoptosis of AML cells and differentiation of LSCs and prevented AML progression in various cellular and animal models [250,263]. In a phase I trial, voruciclib on intermittent dosing was well-tolerated in R/R AML patients [264], and, combined with venetoclax, achieved objective responses in patients with AML [193] (Table 1). Liposomal formulation of flavopiridol was well-tolerated in mice, led to improved plasma concentrations and increased elimination phase half-time relative to flavopiridol alone [265]. This suggests that further increases in flavopiridol retention could result in its improved therapeutic activity.

### 4.5. Combretastatin-A1

Cis-combretastatin-A1 (Figure 2) is a stilbene found in the Eastern Cape South African Bushwillow *Combretum caffrum*. As an anti-angiogenic agent, it destabilizes tubulin, which induces the death of endothelial cells [266]. Cis-combretastatin-A1 diphosphate is a water-soluble prodrug of cis-combretastatin-A1. In vitro, combretastatin-A1 diphosphate counteracted the chemoprotective effects of BM endothelial cells on cytarabine-treated AML cells and induced apoptosis in primary AML cells [267]. The combination of combretastatin-A1 with cytarabine in xenografted human AML models was more effective than either drug alone [268].

In phase I trials in patients with R/R AML, combretastatin-A1 diphosphate showed a manageable safety profile but only a modest anti-leukemic activity as a single agent [194,195] (Table 1). In a multicenter Phase Ib study, combretastatin-A1 diphosphate and cytarabine administered in combination in patients with R/R AML exhibited a manageable toxicity and a promising benefit to risk profile in relapsed AML patients, with a 19% overall response rate, and a considerably longer overall survival for those who obtained complete remission [196,197] (Table 1).

### 4.6. Zosuquidar

Zosuquidar (LY335979) is a synthetic difluorocyclopropyl quinoline alkaloid (Figure 2). The quinoline ring occurs in several NPs found mainly in *Rutaceae* plants (Cinchona Alkaloids), which display a broad range of biological activities, including inhibition of P-gp efflux pump and other related pumps responsible for the development of resistance [269]. Zosuquidar is a highly specific P-gp inhibitor [270]. Compiled in vitro studies demonstrated that zosuquidar restored drug sensitivity in P-gp^+^ AML cell lines, and enhanced the cytotoxicity of anthracyclines (daunorubicin, idarubicin, mitoxantrone) and mylotarg in the majority of primary AML blasts with active P-gp [271,272].

Early clinical studies of zosuquidar given in combination with anthracyclines demonstrated acceptable safety profiles and minimal pharmacokinetic interactions with doxorubicin or daunorubicin [273,274]. Phase I trials have shown that zosuquidar can be given safely to the AML patients in combination with daunorubicin and cytarabine [198,199] (Table 1). In a randomized phase III clinical trial, the combination zosuquidar/daunorubicin/cytarabine did not improve the outcome of elderly patients with newly diagnosed AML [200] (Table 1). However, an open-label, phase I/II multicenter dose escalation study of zosuquidar, combined with daunorubicin and cytarabine in old patients with untreated AML, provided evidence of zosuquidar efficacy with the 72 h continuous intravenous infusion [201] (Table 1). A clinical phase I/II trial in R/R AML evidenced that patients with P-gp^+^/CD33^+^ leukemic blasts were effectively targeted by cotherapy with zosuquidar and mylotarg [137] (Table 1).

### 4.7. Romidepsin

Romidepsin (Figure 2) is a natural bicyclic peptide present in cultures of *Chromobacterium violaceum*, a Gram-negative bacterium isolated from a Japanese soil sample. It is a natural HDAC inhibitor which acts as a prodrug, as its disulfide bridge is reduced by glutathione on uptake into the cell, thus allowing the free thiol groups to interact with Zn^2+^ ions in the active site of class I and II HDAC enzymes [275,276]. HDAC inhibitors, including romidepsin, promoted cell death, apoptosis, autophagy, or cell cycle arrest in preclinical AML models, yet these drugs seemed to be more effective in combination with other drugs than when used as monotherapies [20,204,276].

Romidepsin was first approved for the treatment of cutaneous T-cell lymphoma (TCL) in 2009 by the FDA [275]. Clinical trials in AML with romidepsin had encouraging outcomes in early phase studies [202] (Table 1). In the phase I/II ROMAZA trial, the combination of romidepsin and azacitidine therapy was well-tolerated and clinically active in patients with newly diagnosed R/R AML who are ineligible for conventional chemotherapy [203,204] (Table 1). However, toxicity and tolerability need to be considered in addition to response rate, especially in old patients with AML [20]. Recently, a first-in-class polymer nanoparticle of romidepsin has been developed that exhibits superior pharmacologic properties and superior anti-tumor efficacy in murine xenograft TCL models [277]. The clinical value of nanoromidepsin in AML has yet to be assessed.

### 4.8. Valproic Acid

Valproic acid (Figure 2) is a synthetic molecule derived from valeric acid found naturally in an herbaceous perennial flowering plant, *Valeriana*. This short-chain fatty acid is used as an anticonvulsant to treat epilepsy and bipolar disorder, and to prevent migraine headaches [278]. Valproic acid acts as an HDAC inhibitor by binding to the enzymatic site of HDACs [279]. This molecule induced differentiation and apoptosis in various AML cell lines and primary AML cells expressing P-gp and/or MDR-1, either alone or in combination with agents such as cytarabine, ATRA, and venetoclax [217,280,281,282]. It inhibits HDAC-1 activity in AML blasts, and this effect is accompanied by enhanced levels of histone H3/H4 acetylation [283,284]. Valproic acid increases CAR-T cell toxicity against mouse AML models [285].

A number of clinical trials for AML have been performed on the effects of valproic acid in conjunction with various drugs (Table 1). In phases II/III, valproic acid appears to be a safe and effective treatment option for AML patients, particularly when used in conjunction with ATRA and DNA-hypomethylating drugs (cytarabine, 5-azacytidine) [205,206,207,208,209,210,211,212,213,214,215,216,218,219,220,221,281] (Table 1). Serum samples collected from AML patients included in a previously clinical phase I/II study [217] showed that valproic treatment significantly altered the levels of the amino acid (AA) and lipid metabolites, which may contribute to the antileukemic effects of valproic acid [286].

### 4.9. Lovastatin, Simvastatin, Pravastatin and Pitavastatin

Lovastatin (Figure 2) is a statin initially isolated from a strain of a mold, *Aspergillus terreus*. As a specific inhibitor of hydroxymethylglutaryl-CoA reductase, lovastatin blocks cellular cholesterol synthesis, and hence is extensively used to treat hypercholesterolemia [287]. Lovastatin and its synthetic analogues (such as simvastatin, pravastatin and pitavastatin) (Figure 2) were approved for marketing by the FDA. Lovastatin was shown to inhibit proliferation and colony formation in primary AML cells [288,289,290], as well as to induce apoptosis in AML cells [291,292,293]. Statin-mediated apoptosis is accompanied by the downregulation of Bcl-2 and Raf/MEK/ERK signalings and geranylgeranylation suppression, resulting in upregulation of the pro-apoptotic protein PUMA [289,292,294,295]. Lovastatin enhanced the cytotoxic effect of cytarabine in K-562 cells by downregulating MAPK activity and preventing cytarabine-induced MAPK activation [222]. In line, the majority of CD34^+^ AML primary blasts were affected by lovastatin treatment, which was potentiated when combined with cytarabine and daunorubicin [295]. In AML cell lines, the combination of simvastatin and venetoclax induced more death than either treatment alone [296].

A dose escalation phase I/II trial of lovastatin with high-dose cytarabine for R/R AML was terminated due to slow accrual [222] (Table 1). The combination of pravastatin with cytarabine and idarubicin was successful in two out of three clinical trials for the treatment of newly-diagnosed and relapsed AML patients [223,224,297]. In an initial phase I study, addition of pravastatin to idarubicin and high-dose cytarabine was safe in newly diagnosed and salvage patients with unfavorable or intermediate prognosis cytogenetics [223] (Table 1). In this study, total and LDL (low-density lipoprotein) cholesterol levels decreased in nearly all patients [223]. It has to be pointed out that following exposure to cytotoxic agents, AML blasts elevate cellular cholesterol in a defensive adaptation that increases chemoresistance. Then, inhibition of cholesterol synthesis and uptake could sensitize AML blasts to chemotherapy. A phase II trial of high-dose pravastatin given in combination with idarubicin and cytarabine demonstrated a 30% response rate in untreated and R/R patients with AML, suggesting that targeting the cholesterol pathway may have therapeutic benefit in AML [224] (Table 1). In a phase I/II study, pravastatin with etoposide, cyclosporine and mitoxantrone had adverse effects in patients with R/R AML [225] (Table 1). A phase I study adding pitavastatin to venetoclax-based therapy in new patients with AML showed that pitavastatin was well-tolerated, and toxicities were similar to those of venetoclax-based therapy in standard clinical practice [226] (Table 1).

### 4.10. Metformin

Metformin (Figure 2) is a natural biguanide initially found in *Galega officinalis* (or plant goat’s rue). It is an oral anti-diabetic drug used to treat people with type 2 diabetes at the early stages [298]. Since, the benefits of metformin have been observed for other diseases including cancers. Metformin inhibits cell proliferation in AML cell lines and reduces AML propagation in mice models through the activation of the adenosine monophosphate-activated protein kinase (AMPK) pathway, resulting in the inhibition of downstream mTOR signaling and mitochondrial OXPHOS [299,300,301,302,303]. High-dose metformin promotes AML cell apoptosis in an AMPK-independent manner [304,305,306,307]. When combined with cytarabine or other drugs (i.e., ATRA, paclitaxel, venetoclax, idarubicin, gilteritinib, etc.), metformin may have additive or synergistic cytotoxic effects through AMPK activation, and modulation of distinct signaling pathways (mTORC1/P70S6K; OXPHOS; STAT5; NF-κB; MAP/MAPK; MEK/ERK; Mcl-1, Bcl-x_L_; FLT3/STAT5/ERK/mTOR) [304,305,306,307,308,309,310,311,312,313,314,315].

Two retrospective studies with diabetic AML patients taking metformin showed no metformin benefit [227,228] (Table 1). One phase I clinical trial aimed to test metformin in combination with cytarabine for the treatment of R/R AML. Unfortunately, the study was terminated due to the accrual of only two patients (Table 1).

### 4.11. Acivicin and Acivicin-Prodrug

Acivicin (Figure 2) is a natural structural analogue of glutamine, originally isolated as a fermentation product of *Streptomyces sveceus*. It is a glutamine antimetabolite and a potent inhibitor of the enzymatic activity of γ-glutamyl transpeptidase (γ-GT) activity [316]. The γ-GT is strongly expressed on HL-60 and U937 leukemic cell lines [317,318,319,320,321,322], and on blasts from blood in all AML FAB subtypes (with highest surface activity observed in the FAB M5 group) [317,323,324]. Acivicin inhibited the proliferation of HL-60 and U937 cells and induced their differentiation toward macrophages associated with H_2_O_2_ inhibition and NF-κB activation [318,319,320,321,325]. High-dose acivin induced apoptosis in these cells through H_2_O_2_ inhibition [322,326].

While several phase I studies of acivicin were performed in patients with advanced solid malignancies [327,328], only one phase I clinical trial was conducted, with six patients with R/R AML [229] (Table 1). These clinical trials demonstrated a high rate of severe, albeit reversible, central nervous system toxicity [229,327,328]. A new acivicin prodrug designed for AML-targeted delivery was developed (Figure 2) to target an inactive acivicin precursor to tumor cells when linked to a mAb, which recognizes a tumor-specific antigen at the surface of AML cells. Prodrug cleavage by plasmatic esterases then restored the acivicin’s inhibitory activity against γ-GT and its cytotoxicity towards the HL-60 cell model [329,330]. This suggests that a prodrug-antibody conjugate might be used to target acivicin to AML cells. 

## 5. Preclinical Studies of AML with Selected NPs and NPDs

**Figure 3:**
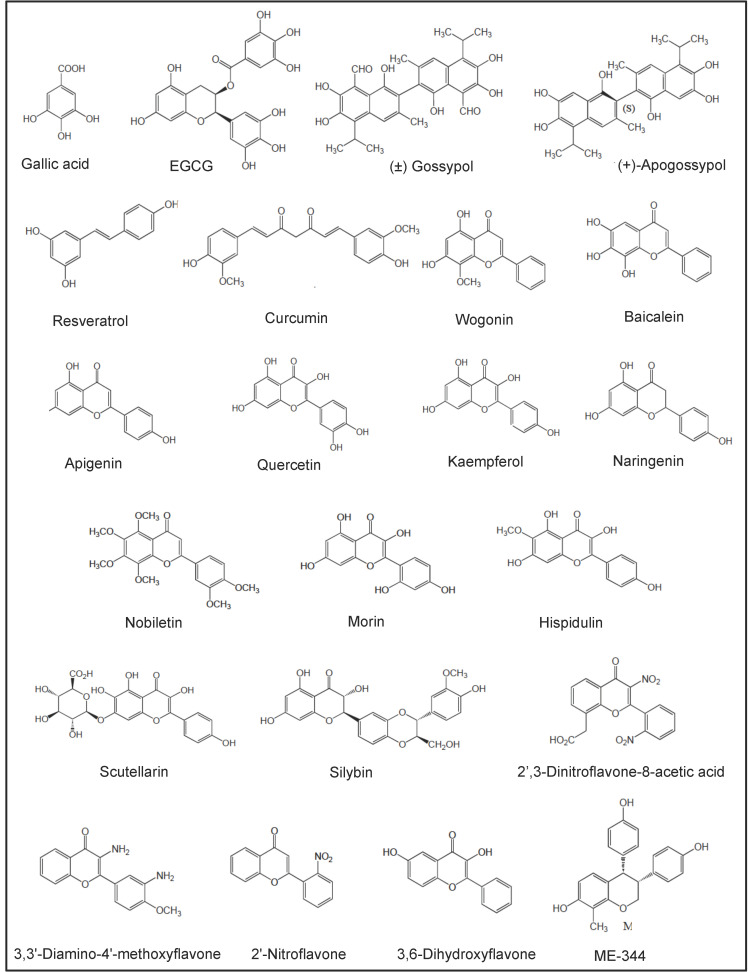
Chemical structures of polyphenols investigated in preclinical studies.

**Figure 4:**
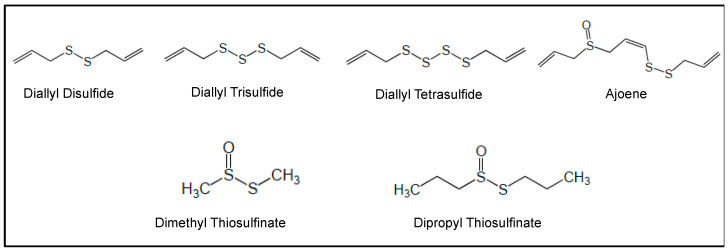
Chemical structures of organosulfur compounds investigated in preclinical studies.

**Figure 5:**
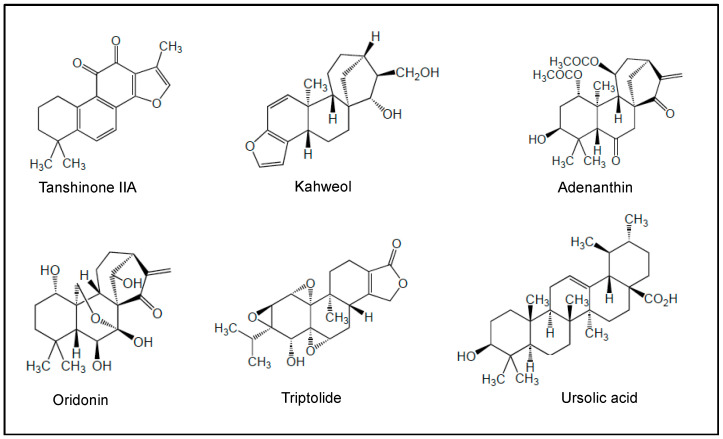
Chemical structures of terpenes investigated in preclinical studies.

**Figure 6:**
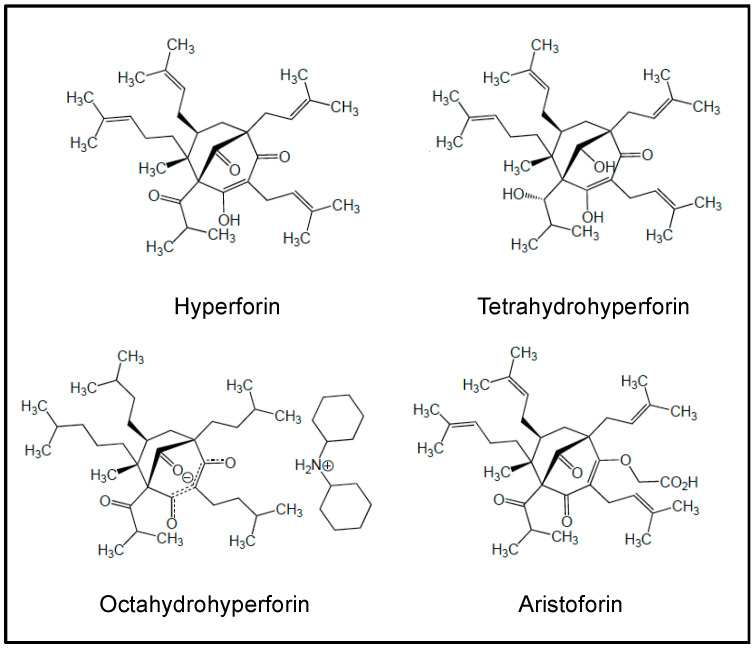
Chemical structures of hyperforin and derivatives investigated in preclinical studies.

In vitro and in vivo studies carried out with a number of NPs and NPDs (Figure 3, Figure 4, Figure 5 and Figure 6 and Table 2) have been performed both in AML models and primary AML samples. Their antitumor effects still need confirmation by clinical trials.

### 5.1. Polyphenols

Polyphenols fall into two main categories, i.e., flavonoids (including flavones, flavonols, flavans, isoflavones, flavanones, catechins, chalcones and anthocyanidins) and non-flavonoids (including stilbenes, curcuminoids, phenolic acids, xanthones, anthraquinones, lignans, coumarin and its derivatives) [438,439,440]. Flavonoids are derived from phenylalanine and initially involve the biosynthesis of 4-coumaroyl-CoA. They are omnipresent in the daily food intake [441,442].

#### 5.1.1. Gallic Acid

Gallic acid (Figure 3) is a polyhydroxyl phenolic compound abundantly found in fruits, grapes, vegetables and green tea. It demonstrated significant growth inhibitory and pro-apoptotic effects in various AML cell lines [331,332,333,334,335,336]. Gallic acid induced apoptosis in AML cell lines and primary CD34^+^ AML cells by inhibiting the AKT/mTOR [335] and NF-κB/BCR-ABL/COX-2 pathways [331,334] or increasing the miR-17/miR-92b/miR-181a pathways [332,333]. It also enhanced the cytotoxic effects of daunorubicin and cytarabine in vitro and in AML xenograft mice [335]. In addition, gallic acid inhibited invasion of K-562 cells related to the downregulation of MMP-2 and MMP-9 expression mediated through the suppression of JNK1/c-Jun/ATF2 and AKT/ERK/c-Jun/c-Fos pathways, respectively [336]. To enhance its therapeutic value, gallic acid has been conjugated with HA, which binds to CD44^+^ AML cells [443]. Doxorubicin and gallic acid coencapsulated in HA-lipid-polymer nanoparticles induced cytotoxicity in doxorubicin-resistant HL-60 cells in vitro and in vivo [444].

#### 5.1.2. (–)-Epigallocatechin-3-Gallate (EGCG)

EGCG (Figure 3) is an abundant biflavanol mainly found in white tea and green tea made from the leaves of the *Camellia sinensis* plant. EGCG is the ester of epigallocatechin and gallic acid. It is a topoisomerase inhibitor and a powerful antioxidant. Preclinical studies have reported the antiproliferative and pro-apoptotic effects of EGCG in AML cell lines and primary AML blasts [337,338,339,340,341,342,343,344,345]. EGCG induces apoptosis by inhibiting Bcl-2 expression and PI3K/AKT activity in HL-60 cells [339,345]. It also inhibits NF-κB expression and increases ROS in UF-1 cells and primary AML cells [342,344]. Moreover, it affects the levels of MDR genes (P-gp and ABCC1) expression in HL-60 cells [345]. EGCG supported differentiation of FLT3-mutated myeloid cell lines toward neutrophils through epigenetic modulations of genes involved in the cell cycle and differentiation (p27, C/EBPα, C/EBPβ) and downregulation of epigenetic regulators DNA methyltransferase-1 and HDAC-1/-2 [338,340,341,342]. In AML xenograft mice, EGCG inhibited AML tumor growth by modulating ROS, BCL-2 proteins (Bax, Bad, Bcl-2) and c-Myc [338,342,344]. Furthermore, EGCG sensitized THP-1 leukemic cells to daunorubicin by inducing the activation of tumor-suppressor proteins, such as retinoblastoma protein and merlin [445].

On the other hand, EGCG has been conjugated with HA, which binds to CD44^+^ AML cells [348]. The HA-EGCG conjugate promoted the terminal differentiation of CD44^+^ myeloid cell lines (NB4 and HL-60 cells), and prolonged survival in the HL-60 xenograft mouse model [348]. A micellar nanocomplex from HA-EGCG and daunorubicin showed superior cytotoxic efficacy against MDR-HL-60 cells over free daunorubicin [346]. The chemosensitizing effect was associated with nucleus accumulation of daunorubicin, elevation of intracellular ROS and caspase-mediated apoptosis induction [346]. A micellar nanocomplex self-assembled from EGCG and sorafenib eradicated BM-residing AML cells in AML xenograft mice, and prolonged survival rates more effectively than free sorafenib [347].

#### 5.1.3. Gossypol

Gossypol (AT-101) (Figure 3) is a natural polyphenol extracted from cotton plants of the genus *Gossypium*. To solve the problems attributable to the presence of the two aldehydes in gossypol, novel derivatives of gossypol were synthesized. Thus, apogossypol (Figure 3), a modified derivative of an atropoisomer of gossypol which lacks the reactive aldehydic groups, was prepared and displays a pro-apoptotic activity comparable to gossypol [446] (Figure 3). Solution-based studies showed that gossypol and apogossypol are capable of binding and inhibiting BCL2 proteins (i.e., Mcl-1, Bcl-2, and Bcl-_XL_) with high affinity [446,447,448].

Both molecules induced caspase-dependent apoptosis in AML cell lines by activating the transcription factors ATF-3/-4 and Noxa, which can then bind to and inhibit Mcl-1 [349,350]. Gossypol was effective towards primary AML cells from patients with adverse prognostic factors (i.e., hyperleukocytosis, FLT3-ITD mutations) and from elderly patients and patients who did not achieve complete remission after induction therapy [350]. Combination of gossypol and idarubicin resulted in synergistic apoptosis of CD34^+^CD38^−^ leukemia stem-like cells (sorted from Kasumi-1 cell lines) and primary LSCs in vitro [351,352], through the inhibition of IL-6/JAK1/STAT3 signaling [352]. The clinical application of gossypol appears limited by poor aqueous solubility, rapid metabolism and systemic toxicity [449]. Various derivatives of gossypol have been developed, such as gossypol Schiff bases and gossypolone, which are less toxic and retain similar therapeutic benefits [450]. Nanocarrier platforms have been engineered to improve gossypol’s therapeutic index in both in vitro and in vivo cancer models [449].

#### 5.1.4. Resveratrol

Resveratrol (3,4′,5-trihydroxystilbene) (Figure 3) is a polyphenol belonging to the class of stilbenes; it is present in the seeds, stems and skins of grapes, nuts, cocoa and certain berries. Resveratrol comes in two isomeric forms, trans- and cis-, with the *trans*-isomer being the bioactive form [451]. Resveratrol exhibits antiproliferative and pro-apoptotic activities in AML cell lines [332,353,354,355,356,357,452]. Resveratrol triggers apoptotic effects in FLT3-ITD AML cells via the inhibition of ceramide catabolism enzymes [452], sensitizes U937 cells to HDAC inhibitors through ROS-mediated activation of the extrinsic apoptotic pathway [357] and increases the expression of miR-181a, miR-17, miR-92b and Bax in HL-60 cells [332]. It suppresses colony-forming cell proliferation of AML BM cells from patients with newly diagnosed AML [359], and induces death in blood AML cells (especially leukemic cells with the FAB M3) [358]. Resveratrol exhibits low water solubility and rapid metabolism, leading to low systemic bioavailability. In order to enhance the cytotoxic effects of resveratrol, a series of analogs and formulations have been developed [453,454,455,456,457], including 3,3′,4,4′,5,5′-hexahydroxystilbene (that induces apoptosis in HL-60 cells through NF-κB inhibition) [454], micronized resveratrol (that allows increased drug absorption, thus increasing availability) [455] and resveratrol encapsulated into nano- and micro-particles [456,457].

#### 5.1.5. Curcumin

Curcumin (Figure 3) is a polyphenolic pigment isolated from the rhizomes of the plant *Curcuma longa*. A number of preclinical studies have showed that curcumin inhibits the growth and induces apoptosis in AML cell lines and primary AML blasts [360,361,362,363,364,365,366,367,368,369,370,371,372,373,458]. Curcumin elicits different signal transduction cascades, including (i) the inhibition of Bcl-2, DNA methyltransferase 1 and survivin [361,363,368,369], (ii) the alteration of the JNK/p38/MAPK/ERK/NF-κB and FLT3/PI3K/AKT pathways [366,370,371,372], and (iii) the downregulation of MMP-2, MMP-9 and VEGF [361,364,366,371]. In line, the growth and invasion of SHI-1 cells in vitro and in vivo appears related to the alteration of MAPK and MMP-2/-9 expression [366]. Curcumin may also promote apoptosis of adriamycin-resistant HL-60 cells by blocking the lncRNA HOTAIR/miR-20a-5p/WT1 axis [373]. In mice implanted with MV4-11 AML cells, administration of curcumin resulted in suppression of the tumor growth [369]. Combined with other drugs (doxorubicin, thalidomide, bortezomib, azacytidine), curcumin induces additive or synergistic cytotoxic effects in AML cell lines [362,364,367] and primary CD34^+^ AML cells [361,367]. In xenograft mouse models, curcumin combined with bortezomib or afuresertib (an AKT inhibitor) suppresses the engraftment, proliferation and survival of AML cells [362,372]. Curcumin exhibits low water solubility and in vivo low bioavailability. This is why a novel curcumin-liposome modified with HA was developed to specifically deliver curcumin to CD44^+^ AML cells [459]. When compared with free curcumin and nontargeted liposome, the HA-curcumin-liposomes exhibited good stability, high affinity to CD44, increased cellular uptake, and more potent activity on inhibiting AML cell proliferation [459].

#### 5.1.6. Other Polyphenols

Several papers reported the ability of other NPs, polyphenols and flavonoids (Figure 3) to induce apoptosis in leukemic myeloid cell lines and primary AML cells through regulation of different signal transduction cascades. These NPs include wogonin and baicalein (isolated from the roots of *Scutellaria baicalensis*), apigenin, quercetin and kaempferol (present in fruits and vegetables), naringenin (present in some citrus fruits), nobiletin (found in citrus peels), morin (found in leaves of *Psidium guajava*), hispidulin (from various plants including *Grindelia argentina*), scutellarin (from plants of the *Scutellaria* family) and silybin (from the milk thistle plant *Silybum marianum*). These molecules were shown to induce caspase-dependent AML cell death associated with (i) the inhibition of the PI3K/AKT and ERK1/2 signaling pathways, (ii) the inhibition of the STAT3 signaling pathway, (iii) the activation of the p38 and/or JNK pathways and (iv) the change of expression of pro-apoptotic (Bad, Bax) and/or anti-apoptotic (Bcl-2, Mcl-1) BCL2 members [383,391,395,460,461]. The VEGF-R2 signaling pathway was also involved in the action of quercetin on mitochondria and Bcl-2 proteins in HL-60 and MV4-11 cells [391]. Quercetin treatment downregulated HDAC-I in HL-60 and U937 cell lines [392]. Furthermore, quercetin induced apoptosis and autophagy in HL-60 leukemic xenografts [396], as well as in primary AML cells by inhibiting PI3K/AKT signaling pathway activation through regulation of miR-224-3p/PTEN axis [395]. Similarly, baicalein induced AML cell autophagy via miR-424 and the PTEN/PI3K/AKT/mTOR pathway [378]. Kaempferol was shown to reduce the expression of MDR genes (P-gp and ABCC1) in HL-60 cells [345,460]. Treatment of THP-1 and KG-1 cells with quercetin or apigenin combined with doxorubicin caused a synergistic downregulation of glutathione levels and increased DNA damage, driving apoptosis [385,386,387].

Several laboratories reported the synthesis of novel flavone derivatives (Figure 3). The 2′,3-dinitroflavone-8-acetic acid was shown to be a noncytotoxic inhibitor of APN activity carried by CD13 in AML cell lines [408] and primary AML blood cells (Nguyen and Bauvois, unpublished results 2008). The 3,3′-diamino-4′-methoxyflavone [462] induced caspase-dependent apoptosis of AML cell lines and suppressed colony-forming cell proliferation of primary AML cells [410]. As a novel proteasome inhibitor, this flavone targeted Bax activation and the degradation of caspase-3 substrate of P70S6K during AML cell death [410]. The 2′-nitroflavone and 3,6-dihydroxyflavone [463,464] induced apoptosis in HL-60 cells through the JNK/ERK1/2/Bax and p38/JNK/ROS signalings [411,413]. ME-344 [4,4′-((3R,4S)-7-Hydroxy-8-methylchroman-3,4-diyl)diphenol] is a second generation of 2*H*-chromene related to phenoxodiol, a synthetic compound related to the natural isoflavone genistein [465]. ME-344 induced apoptosis in leukemia cell lines and primary AML cells (from BM and blood) by increasing mitochondrial ROS generation [414]. It reduced tumor growth in an AML xenograft model [414]. ME-344 also enhanced venetoclax antileukemic activity against leukemic cell lines and primary AML samples, including those resistant to cytarabine, through suppression of OXPHOS and purine biosynthesis [415]. ME-344 is now used as single agent or in combination therapies to treat patients with solid tumors [465], thus supporting its future clinical evaluation in AML patients.

### 5.2. Organosulfur Compounds

Allium vegetables (including garlic, onions, leeks, chives and scallions) are used throughout the world for their apparent health benefits, which are largely attributed to the presence of sulfur compounds in these plants. The major sulfur-containing molecules in intact garlic (*Allium sativum*) are sulfoxides, which are converted into sulfides and thiosulfinates [466,467]. Allicin isolated from garlic is rapidly decomposed to diallyl disulfide, diallyl trisulfide and diallyl tetrasulfide (Figure 4) [466]. These molecules induce apoptosis in AML cell lines [416,417,418,419,420,421,422]. The pro-apoptotic activity of diallyl disulfide may occur through the inhibition of the ERK/p38/MAPK and NF-κB signaling pathways, and ROS increase [418,419,420]. Diallyl tetrasulfide treatment led to the induction of apoptosis by promoting activation of Bax and Bak and counteracting Bcl-x_L_, phospho-Bad and Bcl-2 in U937 cells [422]. Ajoene is another garlic-derived unsaturated disulfide formed by the bonding of three allicin molecules (Figure 4) [423]. Ajoene was shown to inhibit proliferation and induce apoptosis of AML cell lines [423,424]. The addition of ajoene enhanced caspase-dependent apoptosis in both cytarabine- and fludarabine-treated KGI cells through activation of 20S proteasome and inhibition of Bcl-2 expression [425]. The thiosulfinates dimethyl thiosulfinate and dipropyl thiosulfinate are mainly identified in onion and leek (Figure 5) [467]. Both compounds (i) inhibited the growth of AML cell lines (U937, HL-60, NB4, MonoMac-6), (ii) induced differentiation of leukemic cells towards macrophages, and (iii) inhibited the expression of TNF-α and MMP-9 at the post-transcriptional level [426].

### 5.3. Terpenes

Numerous biological characteristics of natural terpenes have been documented, including their antibacterial, antifungal, antiviral, antihyperglycemic, anti-inflammatory and antiparasitic effects, as well as their cancer chemopreventive benefits [468]. Among them, tanshinone IIA (from *Salvia miltiorrhiza*), kahweol (from Arabica coffee), adenanthin (from the leaves of *Rabdosia adenantha*), oridonin (from the herb *Isodon rubescens*, and marine sponges in the genus *Agelas*), triptolide (from the Chinese herb *Tripterygium wilfordii hook F*) and ursolic acid (from many plants such as *Mirabilis jalapa* and numerous fruits and herbs) (Figure 5) have been reported to induce differentiation and apoptosis in AML cell lines and primary AML cells through the modulation of distinct signalings related to (i) the AKT/JNK/ERK signaling pathways, (ii) the activation of C/EBPβ, (iii) the increase in ROS, and (iv) the inhibition of anti-apoptotic BCL2 proteins (Bcl-2, Bcl-x_L_, Mcl-1, XIAP) and upregulation of pro-apoptotic proteins (Bim, Bax) [427,428,429,431,432,469,470]. The combination of oridonin and venetoclax effectively inhibited the growth of AML xenograft tumors in mice and prolonged the survival time of tumor-bearing mice [470].

### 5.4. Hyperforin

The polyprenylated acylphloroglucinol hyperforin (Figure 6) has been isolated from the plant St. John’s wort, *Hypericum perforatum*. As a multi-targeting agent, hyperforin displays antidepressant, antibacterial, anti-oxidant and anti-inflammatory properties [471,472]. It exhibits antiproliferative and pro-apoptotic activities towards AML cell lines and primary AML cells (from blood and BM) independently of the latter’s FAB subtype [81,434,435,471]. Hyperforin was identified as a novel inhibitor of AKT1 activity [435]. Thus, hyperforin’s pro-apoptotic effect in U937 cells involved inhibition of AKT1 signaling, mitochondria and dysfunctions of Bcl-2 members (i.e., Noxa and Bad, which is a direct downstream target of AKT1), thus leading to activation of procaspases -9/-3 [435]. The induction of apoptosis by hyperforin in primary AML blasts significantly paralleled the inhibition of release of MMP-2/-9 and VEGF-A by hyperforin (without altering transcripts) [81]. Hyperforin was also shown to be a potential inhibitor of P-gp functional activity in myeloid cell lines [436]. However, the poor solubility and stability of hyperforin in aqueous solution, as well as its sensitivity to light and oxygen, limits its potential clinical application [473]. Several derivatives with improved stability and ameliorated solubility and pharmacological activities have been next developed, including tetrahydrohyperforin octahydrohyperforin [474] and O-(carboxymethyl)hyperforin, named aristoforin [473] (Figure 6). Encapsulated hyperforin into polymeric nanoparticles shows improved inhibitory bioactivity toward 5-lipoxygenase activity in neutrophils [475]. It remains to be determined whether these analogues retain the anti-leukemic properties of hyperforin in AML models.

## 6. Future Directions for NPs and NPDs in the Context of AML Therapy

This section highlights (i) the relevance of NP/NPDs in prodrug’s strategies, (ii) the interplay between NP/NPDs and AML metabolism and (iii) the identification of additional AML-associated antigens as potential chemotherapeutic targets of NPs and NPDs.

Research efforts opened up the field of NPs and NPDs to prodrug strategies [476,477]. For example, based on the ADC concept (combination of drugs with mAbs), taking advantage of the plasma esterase activity, a prodrug of acivicin was synthesized with a self-immolative spacer capable of releasing acivicin linked to a mAb which can recognize an AML associated-antigen [329,330]. The concept of this prodrug structure could be used with other NPs/NPDs, or conjugated to different AML-associated antigen Abs (directed against CD33, CD13, CD44, etc.), leading to its potential use in AML treatment. Using the pharmacore fusion strategy, bestatin–HDAC inhibitor hybrids were synthesized to obtain novel prodrugs. Among them, two bestatin–vorinostat prodrugs were successfully developed as APN/HDAC dual inhibitors, which exhibit superior antitumor activity compared to bestatin and vorinostat alone [158,166]. In this context, the APN/HDAC inhibitor developed by Jia et al. induces apoptosis in the AML MV4-11 cell line [166]. A third example of prodrug strategy exploited the enzymatic activity of APN/CD13 overexpressed in cancer cells for the activation of melflufen (melphalan-flufenamide) in active cytotoxic melphalan. Melflufen has successfully reached clinical trial phase III in R/R multiple myeloma [478]. In AML, melfufen treatment shows anti-leukemia activity in vitro and in vivo [146]. These examples provide new avenues for the development of NP prodrugs against AML.

As exemplified by CPX-351, the incorporation of bioactive NPs and NPDs into nanodelivery systems can enhance the drug loading capacity, half-life in biological systems, sustained release and selective biodistribution in cancer cells. The nanoformulations developed include polymeric nanoparticles, micelles, nanogels, dendrimers, liposomes, metallic nanoparticles, metallic liposomes and HA-nanoparticles [477,479]. With the focus on AML, two nanosystems were designed and synthesized by incorporating flavopiridol, EGCG and sorafenib. Flavopiridol incorporated into copper-containing liposome exhibited extended circulation lifetimes and demonstrated significant therapeutic activity in subcutaneous AML models [265]. A micellar nanocomplex self-assembled from EGCG and sorafenib successfully eradicated BM-residing AML cells in AML xenograft mice, and prolonged survival rates more effectively than free sorafenib [347]. Besides AML, other studies have demonstrated promising therapeutic activity of nanosystems engineered with polyphenols (including romidepsin, gallic acid, gossypol, resveratrol, curcumin, quercetin, kaempferol), metformin, hyperforin and garlic against solid tumors [265,277,443,449,456,475,480,481,482,483]. These NP/NPD-nanosystems successfully assessed in solid cancers could be evaluated in AML.

A great interest in the potential use of HA-based nanoparticles against CD44^+^ tumors arose when the ability of the natural polysaccharide HA to bind to its primary receptor CD44 overexpressed in cancer cells was demonstrated, thus allowing its efficient internalization in tumor cells [484]. Daunorubicin, doxorubicin, quercetin, EGCG, naringenin, curcumin, resveratrol and gallic acid have been incorporated into HA-based systems and tested in cancer cells of different origins [346,444,459,485,486]. To date, four HA-based nanosystems have been developed and successfully tested in AML models, with doxorubicin crosslinked to HA-lipoic acid-nanoparticles [486], doxorubicin and gallic acid coencapsulated in HA-lipid-polymer nanoparticles [444], daunorubicin and HA-EGCG assembled into micelles [346] and curcumin in HA-liposomes [459]. So far, no NP-based nanomedicines are in clinical use for AML treatment, but undoubtedly, they offer valuable insights for the next generation of AML therapy.

One important insight that deserves attention concerns metabolic reprogramming in AML. The main metabolic pathways dysregulated in AML are glycolysis, AA metabolism, mitochondrial metabolism (OXPHOS) and lipid metabolism, all of which support proliferation and survival of AML cells and LSCs [487,488,489]. In peculiar, LSCs and treatment-resistant cells are highly dependent on mitochondrial metabolism, with high levels of OXPHOS and low levels of ROS [487,488]. As detailed in this review, most NPs and NPDs induced apoptosis in AML cell models by altering distinct metabolic profiles, associated with decreased levels of OXPHOS, COX-1/COX-2, cholesterol, fatty acid (FA) oxidation and increased ROS levels. Elevated levels of ROS (i.e., O^.^_2_, H_2_O_2_, HO^.^ and diverse peroxides) cause damage to lipids, proteins and DNA. Moreover, valproic treatment was shown to alter the levels of the AA and lipid metabolites in serum samples collected from AML patients treated with valproic acid [286]. While clinical trials of metformin or lovastatin, combined with cytarabine for R/R AML, were abandoned due to slow accrual, a phase I study adding pitavastatin to venetoclax-based therapy in new patients with AML showed that pitavastatin was well-tolerated [226]. A phase II trial with pravastatin combined with idarubicin and cytarabine resulted in a high remission rate in relapsed AML patients with favorable risk profiles, associated with inhibition of cholesterol synthesis [224]. In phases II/III, valproic acid appeared to be a safe and effective treatment option for AML patients, particularly when used in conjunction with ATRA and DNA-hypomethylating drugs (Table 1). Clinical trials for AML have evaluated other metabolism-related drugs (which target glycolysis, AA metabolism, OXPHOS and lipid biosynthesis, as well as IDH enzymes involved in the tricarboxylic acid cycle) alone or in combination with other drugs in patients with R/R AML and AML patients with IDH1/2 mutations [489]. Although no metabolic inhibitor has yet been included in standard AML treatment, the value of metabolic-related drugs including NPs/NPDs in the AML metabolic machinery could hold promise in future clinical settings in combination with conventional therapies.

It should be emphasized that the AML-associated antigens CD13, CD33, CD44, VEGF-R2 and P-gp act as ‘’receptors’’ by activating common oncogenic signaling pathways, causing AML cell proliferation, migration, invasion and resistance to chemotherapy. By blocking the expression or the activity of these antigens, tosedostat, mylotarg, quercetin, EGCG and zosuquidar can be considered as ‘’receptor’’ antagonists. The understanding of the molecular pathogenesis of AML has expanded, and allowed the identification of additional AML-associated antigens as potential chemotherapeutic targets. They comprise the chemokine receptor CXCR4, the integrin VLA4, the glycohydrolase CD38, CD47 and CD123, which are overexpressed on primary AML cells and LSCs, and associated with relapse and chemotherapy resistance [490,491,492,493,494]. These antigens regulate AML cell survival and proliferation, mediate AML cell trafficking, and prevent phagocytosis of LSCs, enabling their engraftment in vivo (for CD38 and CD47) [490,491,492,493,494,495]. As we know from solid tumors which share expression of CXCR4, VLA4, CD38, CD47 and CD123, polyphenols (i.e., curcumin, resveratrol, EGCG, naringenin, quercetin, apigenin) are reported to block their expression [440,496,497,498,499,500,501,502,503]. This underlines the potential of NPs and NPDs in targeting CXCR4, VLA4, CD38, CD47 and CD123 involved in AML cell survival and trafficking.

## 7. Conclusions

NPs and NPDs assume an important place in the treatment of AML by offering both opportunities and challenges. Although the list of NPs and NPDs studied in cancers is large and further expanding, this review is not meant to be an exhaustive review of all natural bioactive chemicals acting in the context of AML. Instead, we summarize treatment strategies for AML using selected NPs and NPDs, with promising results in preclinical and clinical studies, which may emerge as new therapeutics in the future.

It appears clear that most drugs used for the routine treatments of AML and conditioning before alloHCT are derived from natural sources (i.e., cytarabine, anthracyclines, midostaurin, calicheamicin linked to anti-CD33, melphalan). Concurrently, other drug candidates, including farnesyl transferase inhibitors, HDAC inhibitors, deoxyadenosine analogues and mAbs derivatives (Ab-drug conjugates, bispecific Abs, and chimeric antigen receptor T cells/CAR-T) are in clinical development for AML therapy [504]. Among the number of NP/NPD drugs tested in clinical trials of AML (Table 1), romidepsin and valproic acid (HDAC inhibitors), pravastatin (statin) and zosuquidar (P-gp inhibitor) have successfully reached clinical trials phases II/III in R/R AML, which raises hope for these agents.

AML remains a challenging malignancy, with chemoresistance contributing to treatment failure and disease relapse. Drug resistance in AML can result to pre-existing genetic alterations that confer intrinsic resistance, or the acquisition of new mutations or phenotypic shifts during treatment in response to the chemotherapeutic treatment [505,506]. In particular, LSCs appear to originate from the acquisition of driver mutations by committed progenitors [507]. Moreover, drug resistance may be caused by drug efflux and metabolic abnormalities. LSCs overexpress P-gp and specifically depend on OXPHOS driven by AA metabolism and/or FA metabolism for their survival [74,507]. Finally, within the BM niche, stromal cells, macrophages and immune cells collectively contribute to tumor inflammation and a hypoxic microenvironment, thereby supporting the persistence and survival of resistant LSCs [508]. This underscores the need to develop more effective drugs to overcome resistance in AML, by targeting P-gp, LSCs and the BM niche. Here, preclinical AML studies indicate that some NPs and NPDs (mainly polyphenols) have the potential to inhibit P-gp activity in AML cells and to target LSCs, dysregulated metabolism and the BM microenvironment.

**Figure 7:**
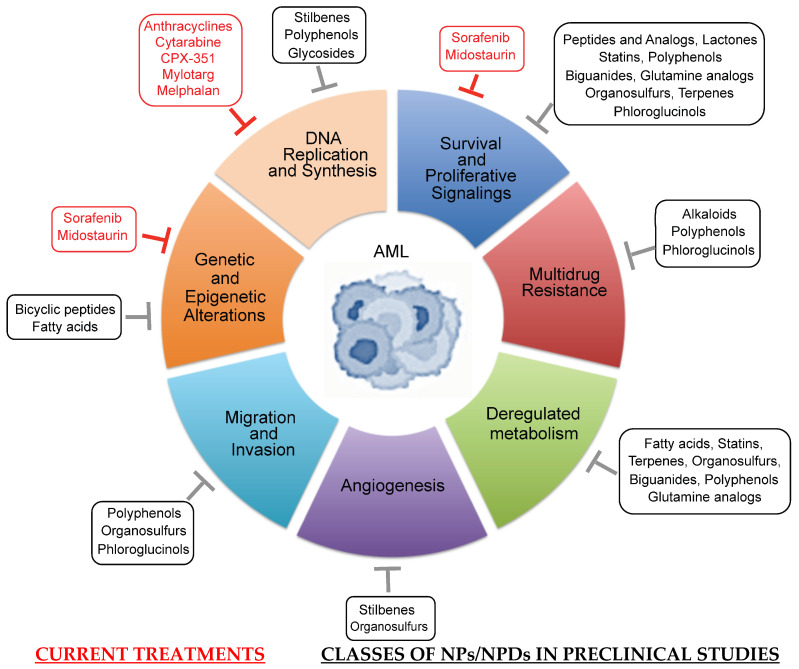
Therapeutic targeting of AML hallmarks. AML is supported by a number of biological capabilities that disable leukemic cell development and progression. NP/NPD drugs currently used in AML treatments interfere mainly with DNA replication/synthesis and proliferative signalings. Different classes of NP/NPD candidates are in preclinical and clinical trials, and may target all hallmarks of AML. One given class may simultaneously target distinct AML processes.

Therapeutic interest in NPs and NPDs is driven by their multiple pharmacological activities identified in AML cells. Figure 7 summarizes the panel of NP/NPD drug candidates targeting AML hallmarks in the context of chemotherapy. By counteracting DNA replication/synthesis and proliferative signalings, NPs and NPDs used in current AML treatments have improved outcomes for many patients. New, effective NPs and NPDs (with multiple modes of action) in combinations with conventional therapeutics are being evaluated in preclinical and clinical studies, and one can expect their participation in the treatment of patients with AML to grow. Integrating the challenges discussed here may further stimulate research in the field of NPs and NPDs as promising drugs towards AML eradication. Finally, all these compounds pave a new avenue for cancer therapy, not only in the field of AML but also in the general treatment of cancer.

## Figures and Tables

**Table 2:** Selected in vitro and in vivo studies of selected NPs and NPDs in AML.

NP/NPD	Class/Activity	Preclinical Study
Gallic acid (NP)	Phenolic acid/antioxidant	Induces apoptosis in HL-60 cells [331,332,333], (COX1/COX2 inhibition) [331], (miRs activation) [332,333] Induces apoptosis in K-562 cells (NF-κB/BCR-ABL/COX2 inhibition) [334]
		Induces apoptosis in THP-1 and MV4-11 cells and CD34^+^ AML cells (AKT/mTOR inhibition) [335] Inhibits invasion of K-562 cells by downregulating MMP-2 and MMP-9 (JNK1/c-Jun/ATF2 and AKT/ERK/c-Jun/c-Fos inhibition) [336]
EGCG (NP) HA-EGCG (NPD)	Biflavanol/topoisomerase inhibitor	Inhibits proliferation of primary AML blasts (c-kit inhibition) [337]
		Inhibits proliferation of THP-1 (FLT3 wild-type) cells (MAPK/AKT/STAT5 inhibition) [338] and (FLT3-ID) MOLM-13/-14 cells (FLT3 downregulation) [338]
		Inhibits proliferation and induces neutrophil differentiation of HL-60 and NB4 cells [338,339,340,341,342], (modulation of p27, C/EBPα,C/EBPɛ, and inhibition of DNMT1 and HDAC1/2) [341,342]
		Induces apoptosis in leukemic cell lines (HL-60, NB4, MOLM-13/-14, KOCL-48 and MV4-11) and primary AML blasts [339,342,343,344,345], (caspase-3/Bcl-2 inhibition) [339,343], (modulation of cyclin D1/NF-κB/ROS/PIN1 axis) [342], (ROS production) [343], (P-gp and ABCC1 inhibition) [345]
		Inhibits AML growth in AML xenograft mice (modulation of ROS, Bax, Bad, Bcl-2, c-Myc) [338,342,344]
		In combination with daunorubicin, enhances cytotoxicity in MDR-HL-60/MX2 cells over free daunorubicin [346]
		In combination with sorafenib, eliminates BM-residing AML cells in AML xenograft mice [347]
		Induces neutrophil differentiation of NB4 and HL-60 cells and prolongs survival in HL-60 xenograft mice [348]
Gossypol (NP)	Phenolic aldehyde/dehydrogenase inhibitor	Induces apoptosis in NB4, K-562, THP-1, and Kasumi-1 cell lines [349,350], (ATF3/4 and NOXA activation, Mcl-1 inhibition) [349], (BCL-2 protein modulation) [350]
		Induces apoptosis in primary CD34^+^ blasts [350]
		Combined with idarubicin, induces apoptosis in CD34^+^CD38^−^ LSCs (sorted from Kasumi-1 cell lines) and primary CD34^+^ AML blasts [351,352], (IL-6/JAK/1/STAT3 inhibition) [352]
Resveratrol (NP)	Stilbene/antioxidant	Induces apoptosis in HL-60 and U937 cells [332,353,354,355,356,357], (increased expression of miR-181a, miR-17, miR-92b and Bax) [332], (ROS production) [357] Induces apoptosis in blood AML cells [358]
		Suppresses colony-forming cell proliferation of primary AML BM cells [359]
Curcumin (NP)	Curcuminoid/antioxidant	Induces apoptosis in HL-60, K-562, U937, Kasumi-1, NB4, KG-1, KG-1a, SHI-1 and OCI-AML3 cell lines [360,361,362,363,364,365,366,367]
		Induces apoptosis in daunorubicin-insensitive CD34+ KG-1a and Kasumi-1 cells, U937 cells and primary CD34^+^ AML cells (Bcl-2 inhibition) [368]
		Induces apoptosis in K-562, HL-60, THP-1, ML-1 and Kasumi-1 cell lines and in primary AML cells (DNA methyltransferase 1 inhibition) [369]
		Induces apoptosis in HL-60, Kasumi-1, NB4 and KG-1 cell lines and inhibits angiogenesis (inhibition of survivin, Bcl-2, MMP-2, MMP-9 and VEGF) [361]
		Combined with doxorubicin, induces increased apoptosis in primary CD34^+^ AML cells [361] and KG-1 and EoL-1 cell lines (FLT3 inhibition) [370]
		Combined with bortezomib, induces additive apoptotic effects in HL-60 cells and xenograft models [362]
		Induces apoptosis in SHI-1 cells (JNK, p38, MAPK and ERK inhibition) and suppresses their invasion (MMP-2/-9 inhibition) [371]
		Enhances arsenic trioxide-induced apoptosis in SKM-1 cells (survivin inhibition) [363]
		Induces apoptosis in KG-1 and U937 cells with an effect stronger in combination with thalidomide, and VEGF inhibition in KG-1 cells [364]
		Combined with azacytidine, induces a synergistic apoptosis in HL-60, U-937, K-562, OCI-AML3 and SHI-1 cell lines and primary AML cells [367]
		Inhibits the growth and invasion of SHI-1 cells in vivo (inhibition of MAPK and MMP-2/-9) [366]
		Induces apoptosis in ML-2 and OCI-AML5 cells (Bcl2/FLT3/PI3K/AKT inhibition) [372]
		Induces apoptosis in adriamycin-resistant HL-60 cells (lncRNA HOTAIR/miR-20a-5p/WT1 inhibition) [373]
Wogonin (NP)	Flavone/antioxidant	Induces apoptosis in HL-60 cells [374,375,376], (Bcl-2/telomerase inhibition) [375], (PI3K/AKT inhibition) [376]
Baicalein (NP)	Flavone/antioxidant	Induces apoptosis in U937, THP-1, Kasumi-1, SKNO-1 and ME-1 cell lines and primary AML cells-bearing NOD/SCID mice models [377]
		Induces autophagy in HL-60 and THP-1 cells (miR424/PTEN/PI3K/AKT/mTOR inhibition) [378]
Apigenin (NP)	Trihydroxyflavone/ antioxidant	Induces apoptosis in HL-60, THP-1, U937 and K-562 cell lines [379,380,381,382,383,384], (PKC/caspase-3 inhibition) [380], (JAK/STAT inhibition) [381], (Hsp27/ERK/p38 inhibition) [382], (AKT/JNK/Mcl-1/Bcl-2 inhibition) [383,384]
		Blocks tumor growth in U937 xenografts [383]
		Combined with etoposide or cyclophosphamide, induces apoptosis in THP-1 and KG-1 cells (Bax activation) [385,386,387]
Quercetin (NP)	Flavonol/antioxidant	Induces apoptosis in KG-1, U937, HL-60, THP-1 and MV4-11 cell lines [256,379,388,389,390,391,392], (modulation of Mcl-1/Bax) [388], (PI3K/AKT/Bcl-2 inhibition) [389], (ERK inhibition and ROS activation) [390], (VEGF-R2/PI3K/AKT/Mcl-1/Bcl-2 inhibition) [391], (HDAC inhibition) [392]
		Suppresses colony-forming cell proliferation of primary AML cells [393,394]
		Induces apoptosis in primary AML cells [389,395], (PI3K/AKT inhibition) [389], (miR-224-3p/PTEN/PI3K/AKT inhibition) [395]
		Reduces tumor growth in mice xenografted with HL-60, U937 cells [388,390,392,396]
		Combined with venetoclax, induces death in KG-1 and Kasumi-1 cells (Bcl-2/Bax modulation) [397]
		Induces autophagy in HL-60 cells and primary AML cells [395,396,398], (CaMKKβ/AMPK/mTOR inhibition) [398]
Kaempferol (NP)	Flavonol/antioxidant	Induces apoptosis in HL-60, K-562 and U937 cell lines [379,399], (inhibition of PI3K/AKT/Bcl-2/Bax axis) [399]
		Induces apoptosis in HL-60 cells and inhibits MDR genes (P-gp and ABCC1) [345]
Naringenin (NP)	Flavanone/antioxidant	Induces apoptosis in THP-1 and HL-60 cell lines [400,401], (PI3K/AKT inhibition) [400], (lncRNA XIST/miR-34a/HDAC1 inhibition) [401]
		Cotreatment of THP-1 cells with curcumin induces apoptosis (AKT/ERK/JNK/p53 inhibition) [402]
Nobiletin (NP)	Flavone/antioxidant	Induces apoptosis in HL-60 cells (MAPK inhibition) [403]
Morin (NP)	Flavonol/antioxidant	Induces apoptosis in U937 cells (Bcl-2/Bax/Bad modulation) [404]
Hispidulin (NP)	Flavone/antioxidant	Induces apoptosis in U937 and HL-60 cells (EMMPRIN inhibition) [405]
Scutellarin (NP)	Flavone heteroside/antioxidant	Induces apoptosis in HL-60 cells (JAK2 and STAT3 inhibition) [406]
Silybin *(NP)*	Flavonolignan/antioxidant	Induces apoptosis in NB4 cells, and binds to the BH3 domain of Bcl-2 [407]
2′,3-dinitroflavone-8-acetic acid (NPD)	Flavone/APN inhibitor	Inhibits APN/CD13 enzymatic activity in cell lines (HL-60, U937, THP-1, MonoMac-6) and primary AML blood cells [408,409]
3,3′-diamino-4′-methoxyflavone (NPD)	Flavone/proteasome inhibitor	Induces apoptosis in U937, OCI-AML3, NB4 and HL-60 cell lines (Bax/P70S6K activation) and inhibits the proteasome’s chymotrypsin-like activity in U937 cells [410]
		Inhibits colony-forming cell proliferation of primary AML cells [410]
2′-nitroflavone (NPD)	Flavone/anti-apoptotic	Induces apoptosis in HL-60 cells (JNK/ERK1/2 inhibition and Bax activation) [411]
3,6-dihydroxyflavone (NPD)	Flavonol/anti-apoptotic	Induces apoptosis in HL-60 cells (p38/JNK inhbition and ROS activation) [412,413]
4,4′-(7-hydroxy-8-methylchroman-3,4-diyl) diphenol/ME-344 (NPD)	Isoflavone/mTOR 1/2 kinase inhibitor	Induces apoptosis in OCI-AML2, NB4, U937, K-562 and HL-60 cell lines and AML blasts [414]
		Reduces tumor growth in OCI-AML2 xenografts [414]
		Enhances venetoclax apoptosis in OCI-AML2, NB4, U937, K-562 and HL-60 cell lines, and AML blasts (OXPHOS inhibition) [415]
Diallyl disulfide (NP)	Organosulfure/antioxidant	Induces apoptosis in HL-60 cells [416,417,418,419,420], (ERK/p38/MAPK inhibition) [418], (NF-κB inhibition) [419], (ROS increase) [420]
Diallyl trisulfide (NP)	Organosulfure/antioxidant	Induces apoptosis in HL-60 cells [421]
Diallyl tetrasulfide (NP)	Organosulfure/antioxidant	Induces apoptosis in U937 cells (Bcl-2/Bcl-xL, Bad/Bax/Bak modulation) [422]
Ajoene (NP)	Organosulfure/antioxidant	Induces apoptosis of HL-60, U937, HEL and OCIM-1 cell lines [423,424], (20S proteasome activation) [424]
		Induces apoptosis in cytarabine-treated KG-1 cells (Bcl-2 inhibition) [425]
Dipropyl Thiosulfinate and Dimethyl Thiosulfinate (NP)	Organosulfure/antioxidant	Inhibit proliferation and induce monocytic differentiation of U937, HL-60, NB4 and MonoMac-6 cell lines, and inhibit TNF-α and MMP-9 expression [426]
Tanshinone IIA (NP)	Terpene/antioxidant	Induces monocytic differentiation and apoptosis in NB4 cells (C-EBPβ activation) [427]
Kahweol (NP)	Diterpene/antioxidant	Induces apoptosis in U937 cells (AKT/JNK/Bcl-2/Bcl-xL/Mcl-1/XIAP inhibition) [428]
Adenanthin (NP)	Diterpene/NF-κB inhibitor	Induces monocytic differentiation of NB4 cells (ERK inhibition and H_2_O_2_ production) [429]
Oridonin (NP)	Diterpene/NF-κB inhibitor	Induces apoptosis in t(8.21) AML cells (ROS production) [430]
Triptolide (NP)	Diterpene triepoxide/NF-κB inhibitor	Sensitizes U937 and OCI-AML-3 cells to TRAIL-induced apoptosis (XIAP activation and p53-mediated increase of DR5) [431]
Ursolic acid (NP)	Triterpene/antioxidant	Induces monocytic differentiation of HL-60 cells (ERK inhibition and C/EBPβ activation) [432]
Hyperforin (NP)	Phloroglucinol/ anti-inflammatory	Induces apoptosis in K-562, U937, OCI-AML3, NB4 and HL-60 cell lines [433,434,435], (AKT1/Bad/Bcl-2/Noxa modulation) [435]
		Induces apoptosis in primary AML blood and BM cells, and inhibits MMP-2/MMP-9/VEGF-A release [81]
		Inhibits P-gp and BCRP activities in HL-60 cells [436]

APN, aminopeptidase-N; BCRP, breast cancer resistance protein; BM, bone marrow; C/EBPβ, CCAAT/enhancer-binding protein beta; DR5, death receptor 5; EMMPRIN, extracellular matrix MMP inducer; COX, cyclooxygenase; HA-EGCG, hyaluronic acid linked to EGCG; HDAC, histone deacetylase; HOTAIR, HOX antisense intergenic RNA; MDR, multidrug resistant; MMP, matrix metalloproteinase; NP, natural product; NP-derivative; OXPHOS, oxidative phosphorylation; ROS, reactive oxygen species; TRAIL, tumor-necrosis-factor related apoptosis inducing ligand; VEGF, vascular endothelial growth factor. FAB subtypes of AML cell lines [437]: M1, AML without maturation, KG-1, K-562; M2, AML with maturation, Kasumi-1, SKNO-1, HL-60; M3, acute promyelocytic leukemia, NB4; M4, acute myelomonocytic leukemia, OCI-AML2, OCI-AML3, OC-AML4, OCI-AML5, Eol-1, KOCL-48, ML-2, ME-1; M5, acute monoblastic and monocytic leukemia, THP-1, U937, MOLM-13, MOLM-14, MV4-11, SHI-1, SKM-1, Mono-Mac6; M6, acute erythoid leukemia, HEL, OCIM-1.

## Data Availability

A literature search was conducted for all English-language literature published up to September 2025. Electronic databases including PubMed and Web of Science were searched with the keywords “acute myeloid leukemia, traditional medicine, natural medicine, chemotherapeutic drugs, natural compound, phytochemical compound, natural product, herbs, cancer treatment, chemotherapy, clinical research” amongst others.

## References

[1] Wachter F., Pikman Y. (2024). Pathophysiology of Acute Myeloid Leukemia. Acta Haematol..

[2] Forsberg M., Konopleva M. (2024). AML treatment: Conventional chemotherapy and emerging novel agents. Trends Pharmacol. Sci..

[3] Kantarjian H.M., DiNardo C.D., Kadia T.M., Daver N.G., Altman J.K., Stein E.M., Jabbour E., Schiffer C.A., Lang A., Ravandi F. (2025). Acute myeloid leukemia management and research in 2025. CA Cancer J. Clin..

[4] Gottardi M., Sperotto A., Ghelli Luserna Di Rorà A., Padella A., Cangini D., Giannini M.B., Simonetti G., Martinelli G., Cerchione C. (2020). Gemtuzumab ozogamicin in acute myeloid leukemia: Past, present and future. Minerva Medica.

[5] Yao K., Liu H., Yu S., Zhu H., Pan J. (2022). Resistance to mutant IDH inhibitors in acute myeloid leukemia: Molecular mechanisms and therapeutic strategies. Cancer Lett..

[6] Zhao J.C., Agarwal S., Ahmad H., Amin K., Bewersdorf J.P., Zeidan A.M. (2022). A review of FLT3 inhibitors in acute myeloid leukemia. Blood Rev..

[7] Antar A., Otrock Z.K., El-Cheikh J., Kharfan-Dabaja M.A., Battipaglia G., Mahfouz R., Mohty M., Bazarbachi A. (2017). Inhibition of FLT3 in AML: A focus on sorafenib. Bone Marrow Transplant..

[8] Nair R., Salinas-Illarena A., Baldauf H.M. (2021). New strategies to treat AML: Novel insights into AML survival pathways and combination therapies. Leukemia.

[9] Atanasov A.G., Zotchev S.B., Dirsch V.M., Supuran C.T. (2021). Natural products in drug discovery: Advances and opportunities. Nat. Rev. Drug Discov..

[10] Banday A.H., Azha N.U., Farooq R., Sheikh S.A., Ganie M.A., Parray M.N., Mushtaq H., Hameed I., Lone M.A. (2024). Exploring the potential of marine natural products in drug development: A comprehensive review. Phytochem. Lett..

[11] Ghosh S., Das S.K., Sinha K., Ghosh B., Sen K., Ghosh N., Sil P.C. (2024). The Emerging Role of Natural Products in Cancer Treatment. Arch. Toxicol..

[12] Martin S.F. (2017). Natural Products and Their Mimics as Targets of Opportunity for Discovery. J. Org. Chem..

[13] Naeem A., Hu P., Yang M., Zhang J., Liu Y., Zhu W., Zheng Q. (2022). Natural Products as Anticancer Agents: Current Status and Future Perspectives. Molecules.

[14] Varghese R., Dalvi Y.B. (2020). Natural Products as Anticancer Agents. Curr. Drug Targets.

[15] Lu X., Friedrich L.J., Efferth T. (2025). Natural products targeting tumour angiogenesis. Br. J. Pharmacol..

[16] Calixto J.B., Otuki M.F., Santos A.R. (2003). Anti-inflammatory compounds of plant origin. Part I. Action on arachidonic acid pathway, nitric oxide and nuclear factor kappa B (NF-kappaB). Planta Med..

[17] Calixto J.B., Campos M.M., Otuki M.F., Santos A.R. (2004). Anti-inflammatory compounds of plant origin. Part II. modulation of pro-inflammatory cytokines, chemokines and adhesion molecules. Planta Med..

[18] Schmid M.C., Varner J.A. (2007). Myeloid cell trafficking and tumor angiogenesis. Cancer Lett..

[19] Haouas H. (2014). Angiogenesis and acute myeloid leukemia. Hematology.

[20] Deng Y., Cheng Q., He J. (2023). HDAC inhibitors: Promising agents for leukemia treatment. Biochem. Biophys. Res. Commun..

[21] Urwanisch L., Unger M.S., Sieberer H., Dang H.H., Neuper T., Regl C., Vetter J., Schaller S., Winkler S.M., Kerschbamer E. (2023). The Class IIA Histone Deacetylase (HDAC) Inhibitor TMP269 Downregulates Ribosomal Proteins and Has Anti-Proliferative and Pro-Apoptotic Effects on AML Cells. Cancers.

[22] Martelli A.M., Nyåkern M., Tabellini G., Bortul R., Tazzari P.L., Evangelisti C., Cocco L. (2006). Phosphoinositide 3-kinase/Akt signaling pathway and its therapeutical implications for human acute myeloid leukemia. Leukemia.

[23] Steelman L.S., Chappell W.H., Abrams S.L., Kempf R.C., Long J., Laidler P., Mijatovic S., Maksimovic-Ivanic D., Stivala F., Mazzarino M.C. (2011). Roles of the Raf/MEK/ERK and PI3K/PTEN/Akt/mTOR pathways in controlling growth and sensitivity to therapy-implications for cancer and aging. Aging.

[24] Shafer D., Grant S. (2016). Update on rational targeted therapy in AML. Blood Rev..

[25] Ozeki K., Kiyoi H., Hirose Y., Iwai M., Ninomiya M., Kodera Y., Miyawaki S., Kuriyama K., Shimazaki C., Akiyama H. (2004). Biologic and clinical significance of the FLT3 transcript level in acute myeloid leukemia. Blood.

[26] Vachhani P., Bose P., Rahmani M., Grant S. (2014). Rational combination of dual PI3K/mTOR blockade and Bcl-2/-xL inhibition in AML. Physiol. Genom..

[27] Sancho M., Leiva D., Lucendo E., Orzáez M. (2022). Understanding MCL1: From cellular function and regulation to pharmacological inhibition. FEBS J..

[28] Spiekermann K., Biethahn S., Wilde S., Hiddemann W., Alves F. (2001). Constitutive activation of STAT transcription factors in acute myelogenous leukemia. Eur. J. Haematol..

[29] Park S., Chapuis N., Tamburini J., Bardet V., Cornillet-Lefebvre P., Willems L., Green A., Mayeux P., Lacombe C., Bouscary D. (2010). Role of the PI3K/AKT and mTOR signaling pathways in acute myeloid leukemia. Haematologica.

[30] Grandage V.L., Gale R.E., Linch D.C., Khwaja A. (2022). Correction: PI3-kinase/Akt is constitutively active in primary acute myeloid leukaemia cells and regulates survival and chemoresistance via NF-kB, MAPkinase and p53 pathways. Leukemia.

[31] Pillinger G., Loughran N.V., Piddock R.E., Shafat M.S., Zaitseva L., Abdul-Aziz A., Lawes M.J., Bowles K.M., Rushworth S.A. (2016). Targeting PI3Kδ and PI3Kγ signalling disrupts human AML survival and bone marrow stromal cell mediated protection. Oncotarget.

[32] Di Francesco B., Verzella D., Capece D., Vecchiotti D., Di Vito Nolfi M., Flati I., Cornice J., Di Padova M., Angelucci A., Alesse E. (2022). NF-κB: A Druggable Target in Acute Myeloid Leukemia. Cancers.

[33] Guzman M.L., Neering S.J., Upchurch D., Grimes B., Howard D.S., Rizzieri D.A., Luger S.M., Jordan C.T. (2001). Nuclear factor-kappaB is constitutively activated in primitive human acute myelogenous leukemia cells. Blood.

[34] Baumgartner B., Weber M., Quirling M., Fischer C., Page S., Adam M., Von Schilling C., Waterhouse C., Schmid C., Neumeier D. (2002). Increased IkappaB kinase activity is associated with activated NF-kappaB in acute myeloid blasts. Leukemia.

[35] Bosman M.C., Schuringa J.J., Vellenga E. (2016). Constitutive NF-κB activation in AML: Causes and treatment strategies. Crit. Rev. Oncol. Hematol..

[36] Mehta S.V., Shukla S.N., Vora H.H. (2013). Overexpression of Bcl2 protein predicts chemoresistance in acute myeloid leukemia: Its correlation with FLT3. Neoplasma.

[37] Zhou J., Chooi J.Y., Ching Y.Q., Quah J.Y., Toh S.H., Ng Y., Tan T.Z., Chng W.J. (2018). NF-κB promotes the stem-like properties of leukemia cells by activation of LIN28B. World J. Stem Cells.

[38] Shi Y., Zhang Z., Qu X., Zhu X., Zhao L., Wei R., Guo Q., Sun L., Yin X., Zhang Y. (2018). Roles of STAT3 in leukemia (Review). Int. J. Oncol..

[39] Cook A.M., Li L., Ho Y., Lin A., Li L., Stein A., Forman S., Perrotti D., Jove R., Bhatia R. (2014). Role of altered growth factor receptor-mediated JAK2 signaling in growth and maintenance of human acute myeloid leukemia stem cells. Blood.

[40] Gil K.J., Borg J., Sheth A.I., Pereira R., Rahkola J., Amaya M.L. (2023). Mitochondrial STAT3 Plays a Critical Role in Survival of AML Cells. Blood.

[41] Cheng H., Chen L., Huang C. (2025). Advances of signal transducer and activator of transcription 3 inhibitors in acute myeloid leukemia (Review). Oncol. Lett..

[42] Amaya M.L., Inguva A., Pei S., Jones C., Krug A., Ye H., Minhajuddin M., Winters A., Furtek S.L., Gamboni F. (2022). The STAT3-MYC axis promotes survival of leukemia stem cells by regulating SLC1A5 and oxidative phosphorylation. Blood.

[43] Wuchter C., Karawajew L., Ruppert V., Büchner T., Schoch C., Haferlach T., Ratei R., Dörken B., Ludwig W.D. (1999). Clinical significance of CD95, Bcl-2 and Bax expression and CD95 function in adult de novo acute myeloid leukemia in context of P-glycoprotein function, maturation stage, and cytogenetics. Leukemia.

[44] Wei Y., Cao Y., Sun R., Cheng L., Xiong X., Jin X., He X., Lu W., Zhao M. (2020). Targeting Bcl-2 Proteins in Acute Myeloid Leukemia. Front. Oncol..

[45] Glaser S.P., Lee E.F., Trounson E., Bouillet P., Wei A., Fairlie W.D., Izon D.J., Zuber J., Rappaport A.R., Herold M.J. (2012). Anti-apoptotic Mcl-1 is essential for the development and sustained growth of acute myeloid leukemia. Genes Dev..

[46] Roberts A.W., Wei A.H., Huang D.C.S. (2021). BCL2 and MCL1 inhibitors for hematologic malignancies. Blood.

[47] Yoshimoto G., Miyamoto T., Jabbarzadeh-Tabrizi S., Iino T., Rocnik J.L., Kikushige Y., Mori Y., Shima T., Iwasaki H., Takenaka K. (2009). FLT3-ITD up-regulates MCL-1 to promote survival of stem cells in acute myeloid leukemia via FLT3-ITD-specific STAT5 activation. Blood.

[48] Breitenbuecher F., Markova B., Kasper S., Carius B., Stauder T., Böhmer F.D., Masson K., Rönnstrand L., Huber C., Kindler T. (2009). A novel molecular mechanism of primary resistance to FLT3-kinase inhibitors in AML. Blood.

[49] Bauvois B. (2001). Transmembrane proteases in focus: Diversity and redundancy?. J. Leukoc. Biol..

[50] Klobusicka M., Kusenda J., Babusikova O. (2005). Myeloid enzymes profile related to the immunophenotypic characteristics of blast cells from patients with acute myeloid leukemia (AML) at diagnosis. Neoplasma.

[51] Taussig D.C., Pearce D.J., Simpson C., Rohatiner A.Z., Lister T.A., Kelly G., Luongo J.L., Danet-Desnoyers G.A., Bonnet D. (2005). Hematopoietic stem cells express multiple myeloid markers: Implications for the origin and targeted therapy of acute myeloid leukemia. Blood.

[52] Bouchet S., Tang R., Fava F., Legrand O., Bauvois B. (2014). Targeting CD13 (aminopeptidase-N) in turn downregulates ADAM17 by internalization in acute myeloid leukaemia cells. Oncotarget.

[53] Bauvois B., Dauzonne D. (2006). Aminopeptidase-N/CD13 (EC 3.4.11.2) inhibitors: Chemistry, biological evaluations, and therapeutic prospects. Med. Res. Rev..

[54] Piedfer M., Dauzonne D., Tang R., N’Guyen J., Billard C., Bauvois B. (2011). Aminopeptidase-N/CD13 is a potential proapoptotic target in human myeloid tumor cells. FASEB J..

[55] Liu J., Tong J., Yang H. (2022). Targeting CD33 for acute myeloid leukemia therapy. BMC Cancer.

[56] Laszlo G.S., Harrington K.H., Gudgeon C.J., Beddoe M.E., Fitzgibbon M.P., Ries R.E., Lamba J.K., McIntosh M.W., Meshinchi S., Walter R.B. (2016). Expression and functional characterization of CD33 transcript variants in human acute myeloid leukemia. Oncotarget.

[57] Ehninger A., Kramer M., Röllig C., Thiede C., Bornhäuser M., von Bonin M., Wermke M., Feldmann A., Bachmann M., Ehninger G. (2014). Distribution and levels of cell surface expression of CD33 and CD123 in acute myeloid leukemia. Blood Cancer J..

[58] Pollard J.A., Alonzo T.A., Loken M., Gerbing R.B., Ho P.A., Bernstein I.D., Raimondi S.C., Hirsch B., Franklin J., Walter R.B. (2012). Correlation of CD33 expression level with disease characteristics and response to gemtuzumab ozogamicin containing chemotherapy in childhood AML. Blood.

[59] Paul S.P., Taylor L.S., Stansbury E.K., McVicar D.W. (2000). Myeloid specific human CD33 is an inhibitory receptor with differential ITIM function in recruiting the phosphatases SHP-1 and SHP-2. Blood.

[60] Balaian L., Zhong R.K., Ball E.D. (2003). The inhibitory effect of anti-CD33 monoclonal antibodies on AML cell growth correlates with Syk and/or ZAP-70 expression. Exp. Hematol..

[61] Legras S., Günthert U., Stauder R., Curt F., Oliferenko S., Kluin-Nelemans H.C., Marie J.P., Proctor S., Jasmin C., Smadja-Joffe F. (1998). A strong expression of CD44-6v correlates with shorter survival of patients with acute myeloid leukemia. Blood.

[62] Chen P., Huang H.F., Lu R., Wu Y., Chen Y.Z. (2012). Prognostic significance of CD44v6/v7 in acute promyelocytic leukemia. Asian Pac. J. Cancer Prev..

[63] Quéré R., Andradottir S., Brun A.C., Zubarev R.A., Karlsson G., Olsson K., Magnusson M., Cammenga J., Karlsson S. (2011). High levels of the adhesion molecule CD44 on leukemic cells generate acute myeloid leukemia relapse after withdrawal of the initial transforming event. Leukemia.

[64] Huang X., Li D., Li T., Zhao B.O., Chen X. (2015). Prognostic value of the expression of phosphatase and tensin homolog and CD44 in elderly patients with refractory acute myeloid leukemia. Oncol. Lett..

[65] Hanke M., Hoffmann I., Christophis C., Schubert M., Hoang V.T., Zepeda-Moreno A., Baran N., Eckstein V., Wuchter P., Rosenhahn A. (2014). Differences between healthy hematopoietic progenitors and leukemia cells with respect to CD44 mediated rolling versus adherence behavior on hyaluronic acid coated surfaces. Biomaterials.

[66] Izzi V., Lakkala J., Devarajan R., Ruotsalainen H., Savolainen E.R., Koistinen P., Heljasvaara R., Pihlajaniemi T. (2017). An extracellular matrix signature in leukemia precursor cells and acute myeloid leukemia. Haematologica.

[67] Sansonetti A., Bourcier S., Durand L., Chomienne C., Smadja-Joffe F., Robert-Lézénès J. (2012). CD44 activation enhances acute monoblastic leukemia cell survival via Mcl-1 upregulation. Leuk. Res..

[68] Chen P., Huang H., Wu J., Lu R., Wu Y., Jiang X., Yuan Q., Chen Y. (2015). Bone marrow stromal cells protect acute myeloid leukemia cells from anti-CD44 therapy partly through regulating PI3K/Akt-p27(Kip1) axis. Mol. Carcinog..

[69] Gutjahr J.C., Bayer E., Yu X., Laufer J.M., Höpner J.P., Tesanovic S., Härzschel A., Auer G., Rieß T., Salmhofer A. (2021). CD44 engagement enhances acute myeloid leukemia cell adhesion to the bone marrow microenvironment by increasing VLA-4 avidity. Haematologica.

[70] Salvia A.M., Cuviello F., Coluzzi S., Nuccorini R., Attolico I., Pascale S.P., Bisaccia F., Pizzuti M., Ostuni A. (2017). Expression of some ATP-binding cassette transporters in acute myeloid leukemia. Hematol. Rep..

[71] Ankathil R. (2017). ABCB1 genetic variants in leukemias: Current insights into treatment outcomes. Pharmacogenom. Pers. Med..

[72] Legrand O., Zompi S., Perrot J.Y., Faussat A.M., Benderra Z., Chaoui D., Marie J.P. (2004). P-glycoprotein and multidrug resistance associated protein-1 activity in 132 acute myeloid leukemias according to FAB subtypes and cytogenetics risk groups. Haematologica.

[73] Marie J.P., Legrand O. (1999). MDR1/P-GP expression as a prognostic factor in acute leukemias. Adv. Exp. Med. Biol..

[74] de Figueiredo-Pontes L.L., Pintão M.C., Oliveira L.C., Dalmazzo L.F., Jácomo R.H., Garcia A.B., Falcão R.P., Rego E.M. (2008). Determination of P-glycoprotein, MDR-related protein 1, breast cancer resistance protein, and lung-resistance protein expression in leukemic stem cells of acute myeloid leukemia. Cytom. Part B Clin. Cytom..

[75] Senent L., Jarque I., Martín G., Sempere A., González-García Y., Gomis F., Pérez-Sirvent M., De La Rubia J., Sanz M.A. (1998). P-glycoprotein expression and prognostic value in acute myeloid leukemia. Haematologica.

[76] Borg A.G., Burgess R., Green L.M., Scheper R.J., Yin J.A. (1998). Overexpression of lung-resistance protein and increased P-glycoprotein function in acute myeloid leukaemia cells predict a poor response to chemotherapy and reduced patient survival. Br. J. Haematol..

[77] Wang X., Bove A.M., Simone G., Ma B. (2020). Molecular Bases of VEGFR-2-Mediated Physiological Function and Pathological Role. Front. Cell Dev. Biol..

[78] Aguayo A., Estey E., Kantarjian H., Mansouri T., Gidel C., Keating M., Giles F., Estrov Z., Barlogie B., Albitar M. (1999). Cellular vascular endothelial growth factor is a predictor of outcome in patients with acute myeloid leukemia. Blood.

[79] Padró T., Bieker R., Ruiz S., Steins M., Retzlaff S., Bürger H., Büchner T., Kessler T., Herrera F., Kienast J. (2002). Overexpression of vascular endothelial growth factor (VEGF) and its cellular receptor KDR (VEGFR-2) in the bone marrow of patients with acute myeloid leukemia. Leukemia.

[80] Hiramatsu A., Miwa H., Shikami M., Ikai T., Tajima E., Yamamoto H., Imai N., Hattori A., Kyo T., Watarai M. (2006). Disease-specific expression of VEGF and its receptors in AML cells: Possible autocrine pathway of VEGF/type1 receptor of VEGF in t(15;17) AML and VEGF/type2 receptor of VEGF in t(8;21) AML. Leuk. Lymphoma.

[81] Merhi F., Tang R., Legrand O., Nguyen-Khac F., Susin S.A., Bauvois B. (2021). The antiangiogenic phloroglucinol hyperforin inhibits the secretion of proMMP-2, proMMP-9 and VEGF-A during apoptosis of primary acute myeloid leukemia cells. J. Cancer Metastasis Treat..

[82] Bellamy W.T., Richter L., Sirjani D., Roxas C., Glinsmann-Gibson B., Frutiger Y., Grogan T.M., List A.F. (2001). Vascular endothelial cell growth factor is an autocrine promoter of abnormal localized immature myeloid precursors and leukemia progenitor formation in myelodysplastic syndromes. Blood.

[83] Ghannadan M., Wimazal F., Simonitsch I., Sperr W.R., Mayerhofer M., Sillaber C., Hauswirth A.W., Gadner H., Chott A., Horny H.P. (2003). Immunohistochemical detection of VEGF in the bone marrow of patients with acute myeloid leukemia. Correlation between VEGF expression and the FAB category. Am. J. Clin. Pathol..

[84] Wang L., Zhang W., Ding Y., Xiu B., Li P., Dong Y., Zhu Q., Liang A. (2015). Up-regulation of VEGF and its receptor in refractory leukemia cells. Int. J. Clin. Exp. Pathol..

[85] Dias S., Hattori K., Zhu Z., Heissig B., Choy M., Lane W., Wu Y., Chadburn A., Hyjek E., Gill M. (2000). Autocrine stimulation of VEGFR-2 activates human leukemic cell growth and migration. J. Clin. Investig..

[86] Dias S., Shmelkov S.V., Lam G., Rafii S. (2002). VEGF(165) promotes survival of leukemic cells by Hsp90-mediated induction of Bcl-2 expression and apoptosis inhibition. Blood.

[87] Santos S.C., Dias S. (2004). Internal and external autocrine VEGF/KDR loops regulate survival of subsets of acute leukemia through distinct signaling pathways. Blood.

[88] Wegiel B., Ekberg J., Talasila K.M., Jalili S., Persson J.L. (2009). The role of VEGF and a functional link between VEGF and p27Kip1 in acute myeloid leukemia. Leukemia.

[89] Liersch R., Schliemann C., Bieker R., Hintelmann H., Buechner T., Berdel W.E., Mesters R.M. (2008). Expression of VEGF-C and its receptor VEGFR-3 in the bone marrow of patients with acute myeloid leukaemia. Leuk. Res..

[90] Chien M.H., Ku C.C., Johansson G., Chen M.W., Hsiao M., Su J.L., Inoue H., Hua K.T., Wei L.H., Kuo M.L. (2009). Vascular endothelial growth factor-C (VEGF-C) promotes angiogenesis by induction of COX-2 in leukemic cells via the VEGF-R3/JNK/AP-1 pathway. Carcinogenesis.

[91] Kampen K.R., Ter Elst A., de Bont E.S. (2013). Vascular endothelial growth factor signaling in acute myeloid leukemia. Cell. Mol. Life Sci..

[92] de Jonge H.J., Valk P.J., Veeger N.J., ter Elst A., den Boer M.L., Cloos J., de Haas V., van den Heuvel-Eibrink M.M., Kaspers G.J., Zwaan C.M. (2010). High VEGFC expression is associated with unique gene expression profiles and predicts adverse prognosis in pediatric and adult acute myeloid leukemia. Blood.

[93] Dias S., Choy M., Alitalo K., Rafii S. (2002). Vascular endothelial growth factor (VEGF)-C signaling through FLT-4 (VEGFR-3) mediates leukemic cell proliferation, survival, and resistance to chemotherapy. Blood.

[94] Hua K.T., Lee W.J., Yang S.F., Chen C.K., Hsiao M., Ku C.C., Wei L.H., Kuo M.L., Chien M.H. (2014). Vascular endothelial growth factor-C modulates proliferation and chemoresistance in acute myeloid leukemic cells through an endothelin-1-dependent induction of cyclooxygenase-2. Biochim. Biophys. Acta.

[95] Brunhoff C., Ekblad J., Persson J.L. (2005). Vascular Endothelial Growth Factor Contributes to Adverse Patient Outcome in Acute Myeloid Leukemia by Promoting Cell Growth and Survival and by Reducing the Sensitivities of Leukemic Cells to Drug-Induced Apoptosis. Blood.

[96] Schepers H., Geugien M., van der Toorn M., Bryantsev A.L., Kampinga H.H., Eggen B.J., Vellenga E. (2005). HSP27 protects AML cells against VP-16-induced apoptosis through modulation of p38 and c-Jun. Exp. Hematol..

[97] Yang L., Dong Z.R., Wen S.P., Pan L., Zhang X.J., Luo J.M., Xu S.R. (2006). Relationship between VEGF and MMP-2, MMP-9 in 82 patients with acute myeloid leukemia. Zhongguo Shi Yan Xue Ye Xue Za Zhi.

[98] Song J.H., Kim S.H., Cho D., Lee I.K., Kim H.J., Kim T.S. (2009). Enhanced invasiveness of drug-resistant acute myeloid leukemia cells through increased expression of matrix metalloproteinase-2. Int. J. Cancer.

[99] Reikvam H., Hatfield K.J., Oyan A.M., Kalland K.H., Kittang A.O., Bruserud O. (2010). Primary human acute myelogenous leukemia cells release matrix metalloproteases and their inhibitors: Release profile and pharmacological modulation. Eur. J. Haematol..

[100] Bauvois B. (2012). New facets of matrix metalloproteinases MMP-2 and MMP-9 as cell surface transducers: Outside-in signaling and relationship to tumor progression. Biochim. Biophys. Acta.

[101] Chaudhary A.K., Chaudhary S., Ghosh K., Shanmukaiah C., Nadkarni A.H. (2016). Secretion and Expression of Matrix Metalloproteinase-2 and 9 from Bone Marrow Mononuclear Cells in Myelodysplastic Syndrome and Acute Myeloid Leukemia. Asian Pac. J. Cancer Prev..

[102] Tolomeo M., Cascio A. (2021). The Multifaced Role of STAT3 in Cancer and Its Implication for Anticancer Therapy. Int. J. Mol. Sci..

[103] Vandooren J., Van den Steen P.E., Opdenakker G. (2013). Biochemistry and molecular biology of gelatinase B or matrix metalloproteinase-9 (MMP-9): The next decade. Crit. Rev. Biochem. Mol. Biol..

[104] Stefanidakis M., Karjalainen K., Jaalouk D.E., Gahmberg C.G., O’Brien S., Pasqualini R., Arap W., Koivunen E. (2009). Role of leukemia cell invadosome in extramedullary infiltration. Blood.

[105] Wang C., Chen Z., Li Z., Cen J. (2010). The essential roles of matrix metalloproteinase-2, membrane type 1 metalloproteinase and tissue inhibitor of metalloproteinase-2 in the invasive capacity of acute monocytic leukemia SHI-1 cells. Leuk. Res..

[106] Pirillo C., Birch F., Tissot F.S., Anton S.G., Haltalli M., Tini V., Kong I., Piot C., Partridge B., Pospori C. (2022). Metalloproteinase inhibition reduces AML growth, prevents stem cell loss, and improves chemotherapy effectiveness. Blood Adv..

[107] Paupert J., Mansat-De Mas V., Demur C., Salles B., Muller C. (2008). Cell-surface MMP-9 regulates the invasive capacity of leukemia blast cells with monocytic features. Cell Cycle.

[108] Sawicki G., Matsuzaki A., Janowska-Wieczorek A. (1998). Expression of the active form of MMP-2 on the surface of leukemic cells accounts for their in vitro invasion. J. Cancer Res. Clin. Oncol..

[109] Schumacher N., Rose-John S. (2022). ADAM17 orchestrates Interleukin-6, TNFα and EGF-R signaling in inflammation and cancer. Biochim. Biophys. Acta Mol. Cell Res..

[110] Kupsa T., Vasatova M., Karesova I., Zak P., Horacek J.M. (2014). Baseline serum levels of multiple cytokines and adhesion molecules in patients with acute myeloid leukemia: Results of a pivotal trial. Exp. Oncol..

[111] Zhou X., Zhou S., Li B., Li Q., Gao L., Li D., Gong Q., Zhu L., Wang J., Wang N. (2015). Transmembrane TNF-α preferentially expressed by leukemia stem cells and blasts is a potent target for antibody therapy. Blood.

[112] Volk A., Li J., Xin J.J., You D. (2013). AML Cells Utilize TNF-Driven JNK Signaling As a Critical NF-κB-Independent Survival Signal. Blood.

[113] Dong Q.M., Ling C., Chen X., Zhao L.I. (2015). Inhibition of tumor necrosis factor-α enhances apoptosis induced by nuclear factor-κB inhibition in leukemia cells. Oncol. Lett..

[114] Zhou X., Li Z., Zhou J. (2017). Tumor necrosis factor α in the onset and progression of leukemia. Exp. Hematol..

[115] Shirley S., Micheau O. (2010). The heme oxygenase-1 and c-FLIP in acute myeloid leukemias: Two non-redundant but mutually exclusive cellular safeguards protecting cells against TNF-induced cell death?. Oncotarget.

[116] Gasparini C., Celeghini C., Monasta L., Zauli G. (2014). NF-κB pathways in hematological malignancies. Cell. Mol. Life Sci..

[117] Shanmugam R., Gade P., Wilson-Weekes A., Sayar H., Suvannasankha A., Goswami C., Li L., Gupta S., Cardoso A.A., Baghdadi T.A. (2012). A noncanonical Flt3ITD/NF-κB signaling pathway represses DAPK1 in acute myeloid leukemia. Clin. Cancer Res..

[118] Murphy T., Yee K.W.L. (2017). Cytarabine and daunorubicin for the treatment of acute myeloid leukemia. Expert Opin. Pharmacother..

[119] Sherif H.A., Magdy A., Elshesheni H.A., Ramadan S.M., Rashed R.A. (2021). Treatment outcome of doxorubicin versus idarubicin in adult acute myeloid leukemia. Leuk. Res. Rep..

[120] Rowe J.M. (2022). The “7+3” regimen in acute myeloid leukemia. Haematologica.

[121] Mayer L.D., Tardi P., Louie A.C. (2019). CPX-351: A nanoscale liposomal co-formulation of daunorubicin and cytarabine with unique biodistribution and tumor cell uptake properties. Int. J. Nanomed..

[122] Mohty R., El Hamed R., Brissot E., Bazarbachi A., Mohty M. (2023). New drugs before, during, and after hematopoietic stem cell transplantation for patients with acute myeloid leukemia. Haematologica.

[123] Alzahrani A.M., Alnuhait M.A., Alqahtani T. (2025). The Clinical Safety and Efficacy of Cytarabine and Daunorubicin Liposome (CPX-351) in Acute Myeloid Leukemia Patients: A Systematic Review. Cancer Rep..

[124] Iyer R., Fetterly G., Lugade A., Thanavala Y. (2010). Sorafenib: A clinical and pharmacologic review. Expert Opin. Pharmacother..

[125] Bazarbachi A., Labopin M., Battipaglia G., Djabali A., Passweg J., Socié G., Forcade E., Blaise D., Chevallier P., Orvain C. (2019). Sorafenib improves survival of FLT3-mutated acute myeloid leukemia in relapse after allogeneic stem cell transplantation: A report of the EBMT Acute Leukemia Working Party. Haematologica.

[126] Weisberg E., Meng C., Case A.E., Sattler M., Tiv H.L., Gokhale P.C., Buhrlage S.J., Liu X., Yang J., Wang J. (2019). Comparison of effects of midostaurin, crenolanib, quizartinib, gilteritinib, sorafenib and BLU-285 on oncogenic mutants of KIT, CBL and FLT3 in haematological malignancies. Br. J. Haematol..

[127] Kim E.S. (2017). Midostaurin: First Global Approval. Drugs.

[128] Levis M. (2017). Midostaurin approved for FLT3-mutated AML. Blood.

[129] Maziarz R.T., Levis M., Patnaik M.M., Scott B.L., Mohan S.R., Deol A., Rowley S.D., Kim D.D.H., Hernandez D., Rajkhowa T. (2021). Midostaurin after allogeneic stem cell transplant in patients with FLT3-internal tandem duplication-positive acute myeloid leukemia. Bone Marrow Transplant..

[130] Borate U., Dvorak-Kornaus K.M., Zhao Q., Walter R.B., Cook R.J., Saultz J.N., Swords R.T., Grieselhuber N.R., Mims A., Larkin K.T. (2024). A Phase I Study to Evaluate the Safety and Tolerability of Gemtuzumab Ozogamicin and Midostaurin When Used in Combination with Standard Cytarabine and Daunorubicin Induction for Newly Diagnosed FLT3-Mutated Acute Myeloid Leukemia. Blood.

[131] Molica M., Perrone S., Mazzone C., Niscola P., Cesini L., Abruzzese E., de Fabritiis P. (2021). CD33 Expression and Gentuzumab Ozogamicin in Acute Myeloid Leukemia: Two Sides of the Same Coin. Cancers.

[132] Bross P.F., Beitz J., Chen G., Chen X.H., Duffy E., Kieffer L., Roy S., Sridhara R., Rahman A., Williams G. (2001). Approval summary: Gemtuzumab ozogamicin in relapsed acute myeloid leukemia. Clin. Cancer Res..

[133] Arain S., Avila A.M., Christian S., Patel P., Sweiss K., Parkin B., Saraf S.L., Rubinstein P.G., Calip G.S., Quigley J.G. (2020). Updated Analyses: Safety and Efficacy of Gemtuzumab Ozogamicin and Venetoclax in Patients with Relapsed or Refractory CD33+ Acute Myeloid Leukemia: A Phase Ib/II Study. Blood.

[134] Schlenk R.F., Paschka P., Krzykalla J., Weber D., Kapp-Schwoerer S., Gaidzik V.I., Leis C., Fiedler W., Kindler T., Schroeder T. (2020). Gemtuzumab Ozogamicin in NPM1-Mutated Acute Myeloid Leukemia: Early Results From the Prospective Randomized AMLSG 09-09 Phase III Study. J. Clin. Oncol..

[135] Godwin C.D., Rodríguez-Arbolí E., Othus M., Halpern A.B., Appelbaum J.S., Percival M.M., Hendrie P.C., Oehler V.G., Keel S.B., Abkowitz J.L. (2022). Phase 1/2 Trial of CLAG-M with Dose-Escalated Mitoxantrone in Combination with Fractionated-Dose Gemtuzumab Ozogamicin for Newly Diagnosed Acute Myeloid Leukemia and Other High-Grade Myeloid Neoplasms. Cancers.

[136] Short N.J., Borthakur G., Pemmaraju N., Dinardo C.D., Kadia T.M., Jabbour E., Konopleva M., Macaron W., Ning J., Ma J. (2022). A multi-arm phase Ib/II study designed for rapid, parallel evaluation of novel immunotherapy combinations in relapsed/refractory acute myeloid leukemia. Leuk. Lymphoma.

[137] Marcelletti J.F., Sikic B.I. (2023). A clinical trial of zosuquidar plus gemtuzumab ozogamicin (GO) in relapsed or refractory acute myeloid leukemia (RR AML): Evidence of efficacy based on leukemic blast P-glycoprotein functional phenotype. Cancer Chemother. Pharmacol..

[138] Weinbergerová B., Čerňan M., Kabut T., Semerád L., Podstavková N., Szotkowski T., Ježíšková I., Mayer J. (2023). Gemtuzumab ozogamicin plus midostaurin in conjunction with standard intensive therapy for FLT3- mutated acute myeloid leukemia patients—Czech center experience. Haematologica.

[139] Röllig C., Schliemann C., Ruhnke L., Fransecky L., Heydrich B.N., Hanoun M., Noppeney R., Schäfer-Eckart K., Wendelin K., Mikesch J.H. (2024). Gemtuzumab ozogamicin plus midostaurin in combination with standard ‘7 + 3’ induction therapy in newly diagnosed AML: Results from the SAL-MODULE phase I study. Br. J. Haematol..

[140] Bergel F.S.J.A. (1954). Cyto-active amino-acid and peptide derivatives. Part I. Substituted phenylalanines. J. Chem. Soc..

[141] Parker J.E., Pagliuca A., Mijovic A., Cullis J.O., Czepulkowski B., Rassam S.M., Samaratunga I.R., Grace R., Gover P.A., Mufti G.J. (1997). Fludarabine, cytarabine, G-CSF and idarubicin (FLAG-IDA) for the treatment of poor-risk myelodysplastic syndromes and acute myeloid leukaemia. Br. J. Haematol..

[142] Guijarro F., Bataller A., Diaz-Beyá M., Garrido A., Coll-Ferrà C., Vives S., Salamero O., Valcárcel D., Tormo M., Arnan M. (2022). Long-term outcomes in patients with relapsed/refractory acute myeloid leukemia and other high-risk myeloid malignancies after undergoing sequential conditioning regimen based on IDA-FLAG and high-dose melphalan. Bone Marrow Transplant..

[143] Gruber I., Koelbl O., Treutwein M., Zeman F., Herr W., Holler E., Edinger M., Wolff D. (2023). Analysis of long-term mortality after total body irradiation-based and melphalan-based chemotherapy conditioning for acute myeloid leukemia. Ann. Hematol..

[144] Wickström M., Nygren P., Larsson R., Harmenberg J., Lindberg J., Sjöberg P., Jerling M., Lehmann F., Richardson P., Anderson K. (2017). Melflufen—A peptidase-potentiated alkylating agent in clinical trials. Oncotarget.

[145] Gullbo J., Dhar S., Luthman K., Ehrsson H., Lewensohn R., Nygren P., Larsson R. (2003). Antitumor activity of the alkylating oligopeptides J1 (L-melphalanyl-p-L-fluorophenylalanine ethyl ester) and P2 (L-prolyl-m-L-sarcolysyl-p-L-fluorophenylalanine ethyl ester): Comparison with melphalan. Anticancer Drugs.

[146] Strese S., Hassan S.B., Velander E., Haglund C., Höglund M., Larsson R., Gullbo J. (2017). In vitro and in vivo anti-leukemic activity of the peptidase-potentiated alkylator melflufen in acute myeloid leukemia. Oncotarget.

[147] Kumari R., Miettinen J.J., Tambe M.B., Jun O., Ruokoranta T., Ikonen N., Suvela M.H., Huppunen M.E., Svensson Gelius S., Acs K. (2024). Efcacy of the Peptide Drug Conjugates Mel ufen and OPDC3 in Venetoclax Resistant Acute Myeloid Leukemi. Blood.

[148] Chen C., Huang Y., Zhang L., Zhou M., Xu Y., Lu J., Cheng Y., Miao Y. (2025). Synergistic antitumor activity of azacitidine and ubenimex on acute myeloid leukemia cells. Aging.

[149] Arimori S., Nagao T., Shimizu Y., Watanabe K., Komatsuda M. (1980). The effect of bestatin on patients with acute and chronic leukemia and malignant lymphoma. Tokai J. Exp. Clin. Med..

[150] Ota K., Kurita S., Yamada K., Masaoka T., Uzuka Y., Ogawa N. (1986). Immunotherapy with bestatin for acute nonlymphocytic leukemia in adults. Cancer Immunol. Immunother..

[151] Ota K., Kurita S., Yamada K., Masaoka T., Uzuka Y., Ogawa N. (1986). Results of follow-up studies on prognosis after immunotherapy with bestatin in acute nonlymphocytic leukemia. Gan Kagaku Ryoho Cancer Chemother..

[152] Hiraoka A., Shibata H., Masaoka T. (1992). Immunopotentiation with Ubenimex for prevention of leukemia relapse after allogeneic BMT. The Study Group of Ubenimex for BMT. Transplant. Proc..

[153] Wei G., Ni W., Chiao J.W., Cai Z., Huang H., Liu D. (2011). A meta-analysis of CAG (cytarabine, aclarubicin, G-CSF) regimen for the treatment of 1029 patients with acute myeloid leukemia and myelodysplastic syndrome. J. Hematol. Oncol..

[154] Saito K., Nakamura Y., Aoyagi M., Waga K., Yamamoto K., Aoyagi A., Inoue F., Nakamura Y., Arai Y., Tadokoro J. (2000). Low-dose cytarabine and aclarubicin in combination with granulocyte colony-stimulating factor (CAG regimen) for previously treated patients with relapsed or primary resistant acute myelogenous leukemia (AML) and previously untreated elderly patients with AML, secondary AML, and refractory anemia with excess blasts in transformation. Int. J. Hematol..

[155] Hirayama Y., Sakamaki S., Takayanagi N., Tsuji Y., Sagawa T., Chiba H., Matsunaga T., Niitsu Y. (2003). Chemotherapy with ubenimex corresponding to patient age and organ disorder for 18 cases of acute myelogeneous leukemia in elderly patients--effects, complications and long-term survival. Gan Kagaku Ryoho Cancer Chemother..

[156] Amin S.A., Adhikari N., Jha T. (2018). Design of Aminopeptidase N Inhibitors as Anti-cancer Agents. J. Med. Chem..

[157] Jiang Y., Li X., Hou J., Huang Y., Wang X., Jia Y., Wang Q., Xu W., Zhang J., Zhang Y. (2018). Synthesis and biological characterization of ubenimex-fluorouracil conjugates for anti-cancer therapy. Eur. J. Med. Chem..

[158] Cao J., Zhao W., Zhao C., Liu Q., Li S., Zhang G., Chou C.J., Zhang Y. (2020). Development of a Bestatin-SAHA Hybrid with Dual Inhibitory Activity against APN and HDAC. Molecules.

[159] Farsa O., Ballayová V., Žáčková R., Kollar P., Kauerová T., Zubáč P. (2022). Aminopeptidase N Inhibitors as Pointers for Overcoming Antitumor Treatment Resistance. Int. J. Mol. Sci..

[160] Löwenberg B., Morgan G., Ossenkoppele G.J., Burnett A.K., Zachée P., Dührsen U., Dierickx D., Müller-Tidow C., Sonneveld P., Krug U. (2010). Phase I/II clinical study of Tosedostat, an inhibitor of aminopeptidases, in patients with acute myeloid leukemia and myelodysplasia. J. Clin. Oncol..

[161] Cortes J., Feldman E., Yee K., Rizzieri D., Advani A.S., Charman A., Spruyt R., Toal M., Kantarjian H. (2013). Two dosing regimens of tosedostat in elderly patients with relapsed or refractory acute myeloid leukaemia (OPAL): A randomised open-label phase 2 study. Lancet. Oncol..

[162] Mawad R., Becker P.S., Hendrie P., Scott B., Wood B.L., Dean C., Sandhu V., Deeg H.J., Walter R., Wang L. (2016). Phase II study of tosedostat with cytarabine or decitabine in newly diagnosed older patients with acute myeloid leukaemia or high-risk MDS. Br. J. Haematol..

[163] DiNardo C.D., Cortes J.E. (2014). Tosedostat for the treatment of relapsed and refractory acute myeloid leukemia. Expert Opin. Investig. Drugs.

[164] Janssen J., Löwenberg B., Manz M., Bargetzi M., Biemond B., von dem Borne P., Breems D., Brouwer R., Chalandon Y., Deeren D. (2021). Inferior Outcome of Addition of the Aminopeptidase Inhibitor Tosedostat to Standard Intensive Treatment for Elderly Patients with AML and High Risk MDS. Cancers.

[165] Dennis M., Burnett A., Hills R., Thomas I., Ariti C., Severinsen M.T., Hemmaway C., Greaves P., Clark R.E., Copland M. (2021). A randomised evaluation of low-dose cytosine arabinoside (ara-C) plus tosedostat versus low-dose ara-C in older patients with acute myeloid leukaemia: Results of the LI-1 trial. Br. J. Haematol..

[166] Jia G., Qi K., Hou B., Yue K., Xu T., Jiang Y., Li X. (2023). Design, synthesis, and biological evaluation of novel HDAC/CD13 dual inhibitors for the treatment of cancer. Eur. J. Med. Chem..

[167] Perl A.E., Kasner M.T., Tsai D.E., Vogl D.T., Loren A.W., Schuster S.J., Porter D.L., Stadtmauer E.A., Goldstein S.C., Frey N.V. (2009). A phase I study of the mammalian target of rapamycin inhibitor sirolimus and MEC chemotherapy in relapsed and refractory acute myelogenous leukemia. Clin. Cancer Res..

[168] Kasner M.T., Mick R., Jeschke G.R., Carabasi M., Filicko-O’Hara J., Flomenberg N., Frey N.V., Hexner E.O., Luger S.M., Loren A.W. (2018). Sirolimus enhances remission induction in patients with high risk acute myeloid leukemia and mTORC1 target inhibition. Investig. New Drugs.

[169] Palmisiano N., Jeschke G., Wilde L., Alpdogan O., Carabasi M., Filicko-O’Hara J., Grosso D., Klumpp T., Martinez U., Wagner J. (2023). A Phase I Trial of Sirolimus with “7&3” Induction Chemotherapy in Patients with Newly Diagnosed Acute Myeloid Leukemia. Cancers.

[170] Amadori S., Stasi R., Martelli A.M., Venditti A., Meloni G., Pane F., Martinelli G., Lunghi M., Pagano L., Cilloni D. (2012). Temsirolimus, an mTOR inhibitor, in combination with lower-dose clofarabine as salvage therapy for older patients with acute myeloid leukaemia: Results of a phase II GIMEMA study (AML-1107). Br. J. Haematol..

[171] Park S., Chapuis N., Saint Marcoux F., Recher C., Prebet T., Chevallier P., Cahn J.Y., Leguay T., Bories P., Witz F. (2013). A phase Ib GOELAMS study of the mTOR inhibitor RAD001 in association with chemotherapy for AML patients in first relapse. Leukemia.

[172] Tan P., Tiong I.S., Fleming S., Pomilio G., Cummings N., Droogleever M., McManus J., Schwarer A., Catalano J., Patil S. (2017). The mTOR inhibitor everolimus in combination with azacitidine in patients with relapsed/refractory acute myeloid leukemia: A phase Ib/II study. Oncotarget.

[173] Burnett A.K., Das Gupta E., Knapper S., Khwaja A., Sweeney M., Kjeldsen L., Hawkins T., Betteridge S.E., Cahalin P., Clark R.E. (2018). Addition of the mammalian target of rapamycin inhibitor, everolimus, to consolidation therapy in acute myeloid leukemia: Experience from the UK NCRI AML17 trial. Haematologica.

[174] Schlenk R.F., Jaramillo S., Müller-Tidow C. (2018). Improving consolidation therapy in acute myeloid leukemia—A tough nut to crack. Haematologica.

[175] Gore M., Powles R., Lakhani A., Milan S., Maitland J., Goss G., Nandi A., Perren T., Forgeson G., Treleaven J. (1989). Treatment of relapsed and refractory acute leukaemia with high-dose cytosine arabinoside and etoposide. Cancer Chemother. Pharmacol..

[176] Aldoss I., Ji L., Haider M., Pullarkat V. (2014). The combination of fludarabine, cytarabine and etoposide is an active and well-tolerated regimen in relapsed/refractory acute myeloid leukemia. Acta Haematol..

[177] Marconi G., Talami A., Abbenante M.C., Paolini S., Sartor C., Parisi S., de Polo S., Nanni J., Bertamini L., Ragaini S. (2018). Mitoxantrone, Etoposide and Cytarabine (MEC) Can Induce Deep Complete Remission and Is an Effective Bridge Therapy to Allotransplantation (SCT) in Refractory/Relapsed Acute Myeloid Leukemia (AML) Patients. Blood.

[178] Halpern A.B., Othus M., Huebner E.M., Buckley S.A., Pogosova-Agadjanyan E.L., Orlowski K.F., Scott B.L., Becker P.S., Hendrie P.C., Chen T.L. (2017). Mitoxantrone, etoposide and cytarabine following epigenetic priming with decitabine in adults with relapsed/refractory acute myeloid leukemia or other high-grade myeloid neoplasms: A phase 1/2 study. Leukemia.

[179] Westhus J., Noppeney R., Schmitz C., Flasshove M., Dührsen U., Hanoun M. (2020). Etoposide Combined with FLAG Salvage Therapy Is Effective in Multiple Relapsed/Refractory Acute Myeloid leukemia. Acta Haematol..

[180] Ma J., Sun S., Wu M., Zuo X., Xie M., Wang X., Ye X.-J., Shen S., Xie Y. (2023). Combination of Decitabine and Etoposide Is Highly Effective in Treating p53-Mutated MDS/AML Via Activating Notch Signaling. Blood.

[181] Ma J., Sun S., Xie Y., Zhou S., Wu M., Zuo X., Xie M., Wang X. (2025). Decitabine with etoposide is effective in TP53 mutated myeloid tumors via overcoming differentiation block. Blood Cancer J..

[182] Karp J.E., Smith B.D., Resar L.S., Greer J.M., Blackford A., Zhao M., Moton-Nelson D., Alino K., Levis M.J., Gore S.D. (2011). Phase 1 and pharmacokinetic study of bolus-infusion flavopiridol followed by cytosine arabinoside and mitoxantrone for acute leukemias. Blood.

[183] Holkova B., Supko J.G., Ames M.M., Reid J.M., Shapiro G.I., Perkins E.B., Ramakrishnan V., Tombes M.B., Honeycutt C., McGovern R.M. (2013). A phase I trial of vorinostat and alvocidib in patients with relapsed, refractory, or poor prognosis acute leukemia, or refractory anemia with excess blasts-2. Clin. Cancer Res..

[184] Karp J.E., Smith B.D., Levis M.J., Gore S.D., Greer J., Hattenburg C., Briel J., Jones R.J., Wright J.J., Colevas A.D. (2007). Sequential flavopiridol, cytosine arabinoside, and mitoxantrone: A phase II trial in adults with poor-risk acute myelogenous leukemia. Clin. Cancer Res..

[185] Karp J.E., Blackford A., Smith B.D., Alino K., Seung A.H., Bolaños-Meade J., Greer J.M., Carraway H.E., Gore S.D., Jones R.J. (2010). Clinical activity of sequential flavopiridol, cytosine arabinoside, and mitoxantrone for adults with newly diagnosed, poor-risk acute myelogenous leukemia. Leuk. Res..

[186] Karp J.E., Garrett-Mayer E., Estey E.H., Rudek M.A., Smith B.D., Greer J.M., Drye D.M., Mackey K., Dorcy K.S., Gore S.D. (2012). Randomized phase II study of two schedules of flavopiridol given as timed sequential therapy with cytosine arabinoside and mitoxantrone for adults with newly diagnosed, poor-risk acute myelogenous leukemia. Haematologica.

[187] Zeidner J.F., Foster M.C., Blackford A.L., Litzow M.R., Morris L.E., Strickland S.A., Lancet J.E., Bose P., Levy M.Y., Tibes R. (2015). Randomized multicenter phase II study of flavopiridol (alvocidib), cytarabine, and mitoxantrone (FLAM) versus cytarabine/daunorubicin (7+3) in newly diagnosed acute myeloid leukemia. Haematologica.

[188] Zeidner J.F., Foster M.C., Blackford A.L., Litzow M.R., Morris L.E., Strickland S.A., Lancet J.E., Bose P., Levy M.Y., Tibes R. (2018). Final results of a randomized multicenter phase II study of alvocidib, cytarabine, and mitoxantrone versus cytarabine and daunorubicin (7 + 3) in newly diagnosed high-risk acute myeloid leukemia (AML). Leuk. Res..

[189] Ikezoe T., Ando K., Onozawa M., Yamane T., Hosono N., Morita Y., Kiguchi T., Iwasaki H., Miyamoto T., Matsubara K. (2022). Phase I study of alvocidib plus cytarabine/mitoxantrone or cytarabine/daunorubicin for acute myeloid leukemia in Japan. Cancer Sci..

[190] Litzow M.R., Wang X.V., Carroll M.P., Karp J.E., Ketterling R.P., Zhang Y., Kaufmann S.H., Lazarus H.M., Luger S.M., Paietta E.M. (2019). A randomized trial of three novel regimens for recurrent acute myeloid leukemia demonstrates the continuing challenge of treating this difficult disease. Am. J. Hematol..

[191] Zeidner J.F., Lin T.L., Vigil C.E., Fine G., Yair Levy M., Nazha A., Esteve J., Lee D.J., Yee K., Dalovisio A. (2021). A prospective biomarker analysis of alvocidib followed by cytarabine and mitoxantrone in MCL-1-dependent relapsed/refractory acute myeloid leukemia. Blood Cancer J..

[192] Jonas B.A., Hou J.Z., Roboz G.J., Alvares C.L., Jeyakumar D., Edwards J.R., Erba H.P., Kelly R.J., Röllig C., Fiedler W. (2023). A phase 1b study of venetoclax and alvocidib in patients with relapsed/refractory acute myeloid leukemia. Hematol. Oncol..

[193] Alvarado-Valero Y., Cook R.J., Dinner S.N., Keng M., Begna K.H., Javidi-Sharifi N., Abedin S., Al Malki M.M., Raj Bhatt V., Rajagopalan P. (2025). The oral CDK9 inhibitor voruciclib combined with venetoclax for patients with relapsed/refractory acute myeloid leukemia. Blood.

[194] Stockton S.S., Pettiford L., Cline C., Chaplin D., Hsu J.W., Wingard J.R., Cogle C.R. (2015). The vascular disrupting agent OXi4503 in relapsed and refractory AML and MDS. Blood.

[195] Cogle C.R., Collins B., Turner D., Pettiford L.C., Bossé R., Hawkins K.E., Beachamp Z., Wise E., Cline C., May W.S. (2020). Safety, feasibility and preliminary efficacy of single agent combretastatin A1 diphosphate (OXi4503) in patients with relapsed or refractory acute myeloid leukemia or myelodysplastic syndromes. Br. J. Haematol..

[196] Stahl M., DeVeaux M., Montesinos P., Itzykson R., Ritchie E.K., Sekeres M.A., Barnard J.D., Podoltsev N.A., Brunner A.M., Komrokji R.S. (2018). Hypomethylating agents in relapsed and refractory AML: Outcomes and their predictors in a large international patient cohort. Blood Adv..

[197] Uckun F.M., Cogle C.R., Lin T.L., Qazi S., Trieu V.N., Schiller G., Watts J.M. (2019). A Phase 1B Clinical Study of Combretastatin A1 Diphosphate (OXi4503) and Cytarabine (ARA-C) in Combination (OXA) for Patients with Relapsed or Refractory Acute Myeloid Leukemia. Cancers.

[198] Gerrard G., Payne E., Baker R.J., Jones D.T., Potter M., Prentice H.G., Ethell M., McCullough H., Burgess M., Mehta A.B. (2004). Clinical effects and P-glycoprotein inhibition in patients with acute myeloid leukemia treated with zosuquidar trihydrochloride, daunorubicin and cytarabine. Haematologica.

[199] Lancet J.E., Baer M.R., Duran G.E., List A.F., Fielding R., Marcelletti J.F., Multani P.S., Sikic B.I. (2009). A phase I trial of continuous infusion of the multidrug resistance inhibitor zosuquidar with daunorubicin and cytarabine in acute myeloid leukemia. Leuk. Res..

[200] Cripe L.D., Uno H., Paietta E.M., Litzow M.R., Ketterling R.P., Bennett J.M., Rowe J.M., Lazarus H.M., Luger S., Tallman M.S. (2010). Zosuquidar, a novel modulator of P-glycoprotein, does not improve the outcome of older patients with newly diagnosed acute myeloid leukemia: A randomized, placebo-controlled trial of the Eastern Cooperative Oncology Group 3999. Blood.

[201] Marcelletti J.F., Sikic B.I. (2024). Continuous 72-h infusion of zosuquidar with chemotherapy in patients with newly diagnosed acute myeloid leukemia stratified for leukemic blast P-glycoprotein phenotype. Cancer Chemother. Pharmacol..

[202] Byrd J.C., Marcucci G., Parthun M.R., Xiao J.J., Klisovic R.B., Moran M., Lin T.S., Liu S., Sklenar A.R., Davis M.E. (2005). A phase 1 and pharmacodynamic study of depsipeptide (FK228) in chronic lymphocytic leukemia and acute myeloid leukemia. Blood.

[203] Craddock C., Tholouli E., Munoz Vicente S., Gbandi E., Houlton A.E., Drummond M.W., Pavlu J., Vyas P. (2017). Safety and Clinical Activity of Combined Romidepsin and Azacitidine Therapy in High Risk Acute Myeloid Leukemia: Preliminary Results of the Romaza Trial. Blood.

[204] Loke J., Metzner M., Boucher R., Jackson A., Hopkins L., Pavlu J., Tholouli E., Drummond M., Peniket A., Bishop R. (2022). Combination romidepsin and azacitidine therapy is well tolerated and clinically active in adults with high-risk acute myeloid leukaemia ineligible for intensive chemotherapy. Br. J. Haematol..

[205] Bug G., Ritter M., Wassmann B., Schoch C., Heinzel T., Schwarz K., Romanski A., Kramer O.H., Kampfmann M., Hoelzer D. (2005). Clinical trial of valproic acid and all-trans retinoic acid in patients with poor-risk acute myeloid leukemia. Cancer.

[206] Kuendgen A., Strupp C., Aivado M., Bernhardt A., Hildebrandt B., Haas R., Germing U., Gattermann N. (2004). Treatment of myelodysplastic syndromes with valproic acid alone or in combination with all-trans retinoic acid. Blood.

[207] Kuendgen A., Knipp S., Fox F., Strupp C., Hildebrandt B., Steidl C., Germing U., Haas R., Gattermann N. (2005). Results of a phase 2 study of valproic acid alone or in combination with all-trans retinoic acid in 75 patients with myelodysplastic syndrome and relapsed or refractory acute myeloid leukemia. Ann. Hematol..

[208] Kuendgen A., Schmid M., Schlenk R., Knipp S., Hildebrandt B., Steidl C., Germing U., Haas R., Dohner H., Gattermann N. (2006). The histone deacetylase (HDAC) inhibitor valproic acid as monotherapy or in combination with all-trans retinoic acid in patients with acute myeloid leukemia. Cancer.

[209] Garcia-Manero G., Kantarjian H.M., Sanchez-Gonzalez B., Yang H., Rosner G., Verstovsek S., Rytting M., Wierda W.G., Ravandi F., Koller C. (2006). Phase 1/2 study of the combination of 5-aza-2′-deoxycytidine with valproic acid in patients with leukemia. Blood.

[210] Blum W., Klisovic R.B., Hackanson B., Liu Z., Liu S., Devine H., Vukosavljevic T., Huynh L., Lozanski G., Kefauver C. (2007). Phase I study of decitabine alone or in combination with valproic acid in acute myeloid leukemia. J. Clin. Oncol..

[211] Issa J.P., Garcia-Manero G., Huang X., Cortes J., Ravandi F., Jabbour E., Borthakur G., Brandt M., Pierce S., Kantarjian H.M. (2015). Results of phase 2 randomized study of low-dose decitabine with or without valproic acid in patients with myelodysplastic syndrome and acute myelogenous leukemia. Cancer.

[212] Corsetti M.T., Salvi F., Perticone S., Baraldi A., De Paoli L., Gatto S., Pietrasanta D., Pini M., Primon V., Zallio F. (2011). Hematologic improvement and response in elderly AML/RAEB patients treated with valproic acid and low-dose Ara-C. Leuk. Res..

[213] Soriano A.O., Yang H., Faderl S., Estrov Z., Giles F., Ravandi F., Cortes J., Wierda W.G., Ouzounian S., Quezada A. (2007). Safety and clinical activity of the combination of 5-azacytidine, valproic acid, and all-trans retinoic acid in acute myeloid leukemia and myelodysplastic syndrome. Blood.

[214] Raffoux E., Cras A., Recher C., Boëlle P.Y., de Labarthe A., Turlure P., Marolleau J.P., Reman O., Gardin C., Victor M. (2010). Phase 2 clinical trial of 5-azacitidine, valproic acid, and all-trans retinoic acid in patients with high-risk acute myeloid leukemia or myelodysplastic syndrome. Oncotarget.

[215] Ryningen A., Stapnes C., Lassalle P., Corbascio M., Gjertsen B.T., Bruserud O. (2009). A subset of patients with high-risk acute myelogenous leukemia shows improved peripheral blood cell counts when treated with the combination of valproic acid, theophylline and all-trans retinoic acid. Leuk. Res..

[216] Skavland J., Jørgensen K.M., Hadziavdic K., Hovland R., Jonassen I., Bruserud O., Gjertsen B.T. (2011). Specific cellular signal-transduction responses to in vivo combination therapy with ATRA, valproic acid and theophylline in acute myeloid leukemia. Blood Cancer J..

[217] Fredly H., Ersvær E., Kittang A.O., Tsykunova G., Gjertsen B.T., Bruserud O. (2013). The combination of valproic acid, all-trans retinoic acid and low-dose cytarabine as disease-stabilizing treatment in acute myeloid leukemia. Clin. Epigenetics.

[218] Lübbert M., Grishina O., Schmoor C., Schlenk R.F., Jost E., Crysandt M., Heuser M., Thol F., Salih H.R., Schittenhelm M.M. (2020). Valproate and Retinoic Acid in Combination With Decitabine in Elderly Nonfit Patients With Acute Myeloid Leukemia: Results of a Multicenter, Randomized, 2 × 2, Phase II Trial. J. Clin. Oncol..

[219] Rummelt C., Grishina O., Schmoor C., Crysandt M., Heuser M., Götze K.S., Schlenk R.F., Döhner K., Salih H.R., Heil G. (2023). Activity of decitabine combined with all-trans retinoic acid in oligoblastic acute myeloid leukemia: Results from a randomized 2x2 phase II trial (DECIDER). Haematologica.

[220] Tassara M., Döhner K., Brossart P., Held G., Götze K., Horst H.A., Ringhoffer M., Köhne C.H., Kremers S., Raghavachar A. (2014). Valproic acid in combination with all-trans retinoic acid and intensive therapy for acute myeloid leukemia in older patients. Blood.

[221] O’Connor T.E., Whitcomb C., Gomez K., Tsai S., Stiff P.J., Hagen P. (2025). Maintenance therapy with azacitidine and valproic acid after allogeneic stem cell transplant in patients with high-risk myelodysplastic syndrome and acute myelogenous leukemia. J. Clin. Oncol..

[222] Holstein S.A., Hohl R.J. (2001). Interaction of cytosine arabinoside and lovastatin in human leukemia cells. Leuk. Res..

[223] Kornblau S.M., Banker D.E., Stirewalt D., Shen D., Lemker E., Verstovsek S., Estrov Z., Faderl S., Cortes J., Beran M. (2007). Blockade of adaptive defensive changes in cholesterol uptake and synthesis in AML by the addition of pravastatin to idarubicin + high-dose Ara-C: A phase 1 study. Blood.

[224] Advani A.S., Li H., Michaelis L.C., Medeiros B.C., Liedtke M., List A.F., O’Dwyer K., Othus M., Erba H.P., Appelbaum F.R. (2018). Report of the relapsed/refractory cohort of SWOG S0919: A phase 2 study of idarubicin and cytarabine in combination with pravastatin for acute myelogenous leukemia (AML). Leuk. Res..

[225] Chen T.L., Estey E.H., Othus M., Gardner K.M., Markle L.J., Walter R.B. (2013). Cyclosporine modulation of multidrug resistance in combination with pravastatin, mitoxantrone and etoposide for adult patients with relapsed/refractory acute myeloid leukemia: A phase 1/2 study. Leuk. Lymphoma.

[226] Brem E.A., Shieh K., Juarez D., Buono R., Jeyakumar D., O’Brien S., Taylor T.H., Fruman D.A. (2024). A phase 1 study adding pitavastatin to venetoclax therapy in AML and CLL/SLL: A mechanism-based drug repurposing strategy. Blood Neoplasia.

[227] Ceacareanu A.C., Nimako G.K., Wintrob Z.A.P. (2017). Missing the Benefit of Metformin in Acute Myeloid Leukemia: A Problem of Contrast?. J. Res. Pharm. Pract..

[228] Tseng C.H. (2020). Metformin Use and Leukemia Risk in Patients With Type 2 Diabetes Mellitus. Front. Endocrinol..

[229] Powell B.L., Craig J.B., Capizzi R.L., Richards F. (1988). Phase I-II trial of acivicin in adult acute leukemia. Investig. New Drugs.

[230] Wullschleger S., Loewith R., Hall M.N. (2006). TOR signaling in growth and metabolism. Cell.

[231] Récher C., Beyne-Rauzy O., Demur C., Chicanne G., Dos Santos C., Mas V.M., Benzaquen D., Laurent G., Huguet F., Payrastre B. (2005). Antileukemic activity of rapamycin in acute myeloid leukemia. Blood.

[232] Boehm A., Aichberger K.J., Mayerhofer M., Krauth M.T., Derdak S., Pickl W.F., Samorapoompichit P., Sillaber C., Florian S., Sonneck K. (2004). Targeting of mTOR in AML Is Associated with Decreased Growth of Leukemic Cells and Downregulation of VEGF. Blood.

[233] Janus A., Linke A., Cebula B., Robak T., Smolewski P. (2009). Rapamycin, the mTOR kinase inhibitor, sensitizes acute myeloid leukemia cells, HL-60 cells, to the cytotoxic effect of arabinozide cytarabine. Anticancer Drugs.

[234] Ryningen A., Reikvam H., Nepstad I., Paulsen Rye K., Bruserud O. (2012). Inhibition of Mammalian target of rapamycin in human acute myeloid leukemia cells has diverse effects that depend on the environmental in vitro stress. Bone Marrow Res..

[235] Boehm A., Mayerhofer M., Herndlhofer S., Knoebl P., Sillaber C., Sperr W.R., Jaeger U., Valent P. (2009). Evaluation of in vivo antineoplastic effects of rapamycin in patients with chemotherapy-refractory AML. Eur. J. Intern. Med..

[236] Rini B.I. (2008). Temsirolimus, an inhibitor of mammalian target of rapamycin. Clin. Cancer Res..

[237] Lévy A., Sauvin L.A., Massard C., Soria J.C. (2008). Everolimus (RAD001) and solid tumours: A 2008 summary. Bull. Du Cancer.

[238] Zeng Z., Sarbassov D.D., Samudio I.J., Yee K.W., Munsell M.F., Ellen Jackson C., Giles F.J., Sabatini D.M., Andreeff M., Konopleva M. (2007). Rapamycin derivatives reduce mTORC2 signaling and inhibit AKT activation in AML. Blood.

[239] Xu Q., Thompson J.E., Carroll M. (2005). mTOR regulates cell survival after etoposide treatment in primary AML cells. Blood.

[240] Chiarini F., Lonetti A., Teti G., Orsini E., Bressanin D., Cappellini A., Ricci F., Tazzari P.L., Ognibene A., Falconi M. (2012). A combination of temsirolimus, an allosteric mTOR inhibitor, with clofarabine as a new therapeutic option for patients with acute myeloid leukemia. Oncotarget.

[241] Zhang W., Gou P., Dupret J.M., Chomienne C., Rodrigues-Lima F. (2021). Etoposide, an anticancer drug involved in therapy-related secondary leukemia: Enzymes at play. Transl. Oncol..

[242] Chang T.T., Gulati S., Chou T.C., Colvin M., Clarkson B. (1987). Comparative cytotoxicity of various drug combinations for human leukemic cells and normal hematopoietic precursors. Cancer Res..

[243] Edwards C.M., Glisson B.S., King C.K., Smallwood-Kentro S., Ross W.E. (1987). Etoposide-induced DNA cleavage in human leukemia cells. Cancer Chemother. Pharmacol..

[244] Chiron M., Demur C., Pierson V., Jaffrezou J.P., Muller C., Saivin S., Bordier C., Bousquet C., Dastugue N., Laurent G. (1992). Sensitivity of fresh acute myeloid leukemia cells to etoposide: Relationship with cell growth characteristics and DNA single-strand breaks. Blood.

[245] Gieseler F., Glasmacher A., Kämpfe D., Wandt H., Nuessler V., Valsamas S., Kunze J., Wilms K. (1996). Topoisomerase II activities in AML blasts and their correlation with cellular sensitivity to anthracyclines and epipodophyllotoxines. Leukemia.

[246] Fujita N., Tsuruo T. (1998). Involvement of Bcl-2 cleavage in the acceleration of VP-16-induced U937 cell apoptosis. Biochem. Biophys. Res. Commun..

[247] Bruni E., Reichle A., Scimeca M., Bonanno E., Ghibelli L. (2018). Lowering Etoposide Doses Shifts Cell Demise From Caspase-Dependent to Differentiation and Caspase-3-Independent Apoptosis via DNA Damage Response, Inducing AML Culture Extinction. Front. Pharmacol..

[248] Bennett J.M., Lymann G.H., Cassileth P.A., Glick J.H., Oken M.M. (1984). A phase II trial of VP 16-213 in adults with refractory acute myeloid leukemia. An Eastern Cooperative Oncology Group study. Am. J. Clin. Oncol..

[249] Ho A.D., Brado B., Haas R., Hunstein W. (1991). Etoposide in acute leukemia. Past experience and future perspectives. Cancer.

[250] Joshi H., Tuli H.S., Ranjan A., Chauhan A., Haque S., Ramniwas S., Bhatia G.K., Kandari D. (2023). The Pharmacological Implications of Flavopiridol: An Updated Overview. Molecules.

[251] Takada Y., Aggarwal B.B. (2004). Flavopiridol inhibits NF-kappaB activation induced by various carcinogens and inflammatory agents through inhibition of IkappaBalpha kinase and p65 phosphorylation: Abrogation of cyclin D1, cyclooxygenase-2, and matrix metalloprotease-9. J. Biol. Chem..

[252] Parker B.W., Kaur G., Nieves-Neira W., Taimi M., Kohlhagen G., Shimizu T., Losiewicz M.D., Pommier Y., Sausville E.A., Senderowicz A.M. (1998). Early induction of apoptosis in hematopoietic cell lines after exposure to flavopiridol. Blood.

[253] Decker R.H., Dai Y., Grant S. (2001). The cyclin-dependent kinase inhibitor flavopiridol induces apoptosis in human leukemia cells (U937) through the mitochondrial rather than the receptor-mediated pathway. Cell Death Differ..

[254] Rapoport A.P., Simons-Evelyn M., Chen T., Sidell R., Luhowskyj S., Rosell K., Obrig T., Hicks D., Hinkle P.M., Nahm M. (2001). Flavopiridol induces apoptosis and caspase-3 activation of a newly characterized Burkitt’s lymphoma cell line containing mutant p53 genes. Blood Cells Mol. Dis..

[255] Rosato R.R., Almenara J.A., Cartee L., Betts V., Chellappan S.P., Grant S. (2002). The cyclin-dependent kinase inhibitor flavopiridol disrupts sodium butyrate-induced p21WAF1/CIP1 expression and maturation while reciprocally potentiating apoptosis in human leukemia cells. Mol. Cancer Ther..

[256] Liesveld J.L., Abboud C.N., Lu C., McNair C., Menon A., Smith A., Rosell K., Rapoport A.P. (2003). Flavonoid effects on normal and leukemic cells. Leuk. Res..

[257] Smith B.D., Warner S.L., Whatcott C., Siddiqui-Jain A., Bahr B., Dettman E., Doykan C., Cardone M.H., Weitman S.D., Bearss D. (2015). An alvocidib-containing regimen is highly effective in AML patients through a mechanism dependent on MCL1 expression and function. J. Clin. Oncol..

[258] Karp J.E., Passaniti A., Gojo I., Kaufmann S., Bible K., Garimella T.S., Greer J., Briel J., Smith B.D., Gore S.D. (2005). Phase I and pharmacokinetic study of flavopiridol followed by 1-beta-D-arabinofuranosylcytosine and mitoxantrone in relapsed and refractory adult acute leukemias. Clin. Cancer Res..

[259] Blum W., Phelps M.A., Klisovic R.B., Rozewski D.M., Ni W., Albanese K.A., Rovin B., Kefauver C., Devine S.M., Lucas D.M. (2010). Phase I clinical and pharmacokinetic study of a novel schedule of flavopiridol in relapsed or refractory acute leukemias. Haematologica.

[260] Lee Y.K., Tidwell M.L., Kaufmann S.H., Karp J.E., Bible K.C. (2002). Flavopiridol disrupts STAT-3/DNA binding and down-regulates the downstream antiapoptotic protein MCL-1 in vivo in leukemic cells from flavopiridol-treated AML patients. Proc. Am. Assoc. Cancer Res..

[261] Karp J.E., Ross D.D., Yang W., Tidwell M.L., Wei Y., Greer J., Mann D.L., Nakanishi T., Wright J.J., Colevas A.D. (2003). Timed sequential therapy of acute leukemia with flavopiridol: In vitro model for a phase I clinical trial. Clin. Cancer Res..

[262] Nelson D.M., Joseph B., Hillion J., Segal J., Karp J.E., Resar L.M. (2011). Flavopiridol induces BCL-2 expression and represses oncogenic transcription factors in leukemic blasts from adults with refractory acute myeloid leukemia. Leuk. Lymphoma.

[263] Sorf A., Sucha S., Morell A., Novotna E., Staud F., Zavrelova A., Visek B., Wsol V., Ceckova M. (2020). Targeting Pharmacokinetic Drug Resistance in Acute Myeloid Leukemia Cells with CDK4/6 Inhibitors. Cancers.

[264] Davids M.S., Brander D.M., Alvarado-Valero Y., Diefenbach C.S., Egan D.N., Dinner S.N., Javidi-Sharifi N., Al Malki M.M., Begna K.H., Bhatt V.R. (2025). A phase 1 study of the CDK9 inhibitor voruciclib in relapsed/refractory acute myeloid leukemia and B-cell malignancies. Blood Adv..

[265] Chen K.T.J., Militao G.G.C., Anantha M., Witzigmann D., Leung A.W.Y., Bally M.B. (2021). Development and characterization of a novel flavopiridol formulation for treatment of acute myeloid leukemia. J. Control. Release.

[266] Holwell S.E., Cooper P.A., Thompson M.J., Pettit G.R., Lippert L.W., Martin S.W., Bibby M.C. (2002). Anti-tumor and anti-vascular effects of the novel tubulin-binding agent combretastatin A-1 phosphate. Anticancer Res..

[267] Madlambayan G.J., Meacham A.M., Hosaka K., Mir S., Jorgensen M., Scott E.W., Siemann D.W., Cogle C.R. (2010). Leukemia regression by vascular disruption and antiangiogenic therapy. Blood.

[268] Benezra M., Phillips E., Tilki D., Ding B.S., Butler J., Dobrenkov K., Siim B., Chaplin D., Rafii S., Rabbany S. (2012). Serial monitoring of human systemic and xenograft models of leukemia using a novel vascular disrupting agent. Leukemia.

[269] Joshi P., Vishwakarma R.A., Bharate S.B. (2017). Natural alkaloids as P-gp inhibitors for multidrug resistance reversal in cancer. Eur. J. Med. Chem..

[270] Labbozzetta M., Poma P., Notarbartolo M. (2023). Natural Inhibitors of P-glycoprotein in Acute Myeloid Leukemia. Int. J. Mol. Sci..

[271] Tang R., Faussat A.M., Perrot J.Y., Marjanovic Z., Cohen S., Storme T., Morjani H., Legrand O., Marie J.P. (2008). Zosuquidar restores drug sensitivity in P-glycoprotein expressing acute myeloid leukemia (AML). BMC Cancer.

[272] Marcelletti J.F., Multani P.S., Lancet J.E., Baer M.R., Sikic B.I. (2009). Leukemic blast and natural killer cell P-glycoprotein function and inhibition in a clinical trial of zosuquidar infusion in acute myeloid leukemia. Leuk. Res..

[273] Rubin E.H., de Alwis D.P., Pouliquen I., Green L., Marder P., Lin Y., Musanti R., Grospe S.L., Smith S.L., Toppmeyer D.L. (2002). A phase I trial of a potent P-glycoprotein inhibitor, Zosuquidar.3HCl trihydrochloride (LY335979), administered orally in combination with doxorubicin in patients with advanced malignancies. Clin. Cancer Res..

[274] Sandler A., Gordon M., De Alwis D.P., Pouliquen I., Green L., Marder P., Chaudhary A., Fife K., Battiato L., Sweeney C. (2004). A Phase I trial of a potent P-glycoprotein inhibitor, zosuquidar trihydrochloride (LY335979), administered intravenously in combination with doxorubicin in patients with advanced malignancy. Clin. Cancer Res..

[275] VanderMolen K.M., McCulloch W., Pearce C.J., Oberlies N.H. (2011). Romidepsin (Istodax, NSC 630176, FR901228, FK228, depsipeptide): A natural product recently approved for cutaneous T-cell lymphoma. J. Antibiot..

[276] San José-Enériz E., Gimenez-Camino N., Agirre X., Prosper F. (2019). HDAC Inhibitors in Acute Myeloid Leukemia. Cancers.

[277] Pal I., Illendula A., Joyner A.M., Manavalan J.S., Deddens T.M., Sabzevari A., Damera D.P., Zuberi S., Marchi E., Fox T.E. (2025). Nanoromidepsin, a Polymer Nanoparticle of the HDAC Inhibitor, Improves Safety and Efficacy in Models of T-cell Lymphoma. Blood.

[278] Safdar A., Ismail F. (2023). A comprehensive review on pharmacological applications and drug-induced toxicity of valproic acid. Saudi Pharm. J..

[279] Duenas-Gonzalez A., Candelaria M., Perez-Plascencia C., Perez-Cardenas E., de la Cruz-Hernandez E., Herrera L.A. (2008). Valproic acid as epigenetic cancer drug: Preclinical, clinical and transcriptional effects on solid tumors. Cancer Treat. Rev..

[280] Kuendgen A., Gattermann N. (2007). Valproic acid for the treatment of myeloid malignancies. Cancer.

[281] Fredly H., Gjertsen B.T., Bruserud O. (2013). Histone deacetylase inhibition in the treatment of acute myeloid leukemia: The effects of valproic acid on leukemic cells, and the clinical and experimental evidence for combining valproic acid with other antileukemic agents. Clin. Epigenet..

[282] Kawakatsu R., Tadagaki K., Yamasaki K., Kuwahara Y., Yoshida T. (2025). Valproic Acid Enhances Venetoclax Efficacy in Targeting Acute Myeloid Leukemia. Diseases.

[283] Bots M., Verbrugge I., Martin B.P., Salmon J.M., Ghisi M., Baker A., Stanley K., Shortt J., Ossenkoppele G.J., Zuber J. (2014). Differentiation therapy for the treatment of t(8;21) acute myeloid leukemia using histone deacetylase inhibitors. Blood.

[284] Liss A., Ooi C.H., Zjablovskaja P., Benoukraf T., Radomska H.S., Ju C., Wu M., Balastik M., Delwel R., Brdicka T. (2014). The gene signature in CCAAT-enhancer-binding protein α dysfunctional acute myeloid leukemia predicts responsiveness to histone deacetylase inhibitors. Haematologica.

[285] Wen J., Chen Y., Yang J., Dai C., Yu S., Zhong W., Liu L., He C., Zhang W., Yang T. (2023). Valproic acid increases CAR T cell cytotoxicity against acute myeloid leukemia. J. Immunother. Cancer.

[286] Grønningsæter I.S., Fredly H.K., Gjertsen B.T., Hatfield K.J., Bruserud Ø. (2019). Systemic Metabolomic Profiling of Acute Myeloid Leukemia Patients before and During Disease-Stabilizing Treatment Based on All-Trans Retinoic Acid, Valproic Acid, and Low-Dose Chemotherapy. Cells.

[287] Sizar O., Khare S., Patel P., Talati R. (2025). Statin Medications. StatPearls.

[288] Scheffold C., Schöttker B., Lefterova P., Csipai M., Glasmacher A., Huhn D., Neubauer A., Schmidt-Wolf I.G. (2000). Increased sensitivity of myeloid leukemia cell lines: Potential of lovastatin as bone-marrow-purging agent. Acta Haematol..

[289] Wong W.W., Dimitroulakos J., Minden M.D., Penn L.Z. (2002). HMG-CoA reductase inhibitors and the malignant cell: The statin family of drugs as triggers of tumor-specific apoptosis. Leukemia.

[290] Stirewalt D.L., Appelbaum F.R., Willman C.L., Zager R.A., Banker D.E. (2003). Mevastatin can increase toxicity in primary AMLs exposed to standard therapeutic agents, but statin efficacy is not simply associated with ras hotspot mutations or overexpression. Leuk. Res..

[291] Dimitroulakos J., Nohynek D., Backway K.L., Hedley D.W., Yeger H., Freedman M.H., Minden M.D., Penn L.Z. (1999). Increased sensitivity of acute myeloid leukemias to lovastatin-induced apoptosis: A potential therapeutic approach. Blood.

[292] Wu J., Wong W.W., Khosravi F., Minden M.D., Penn L.Z. (2004). Blocking the Raf/MEK/ERK pathway sensitizes acute myelogenous leukemia cells to lovastatin-induced apoptosis. Cancer Res..

[293] Burke L.P., Kukoly C.A. (2008). Statins induce lethal effects in acute myeloblastic leukemia cells within 72 hours. Leuk. Lymphoma.

[294] Xia Z., Tan M.M., Wong W.W., Dimitroulakos J., Minden M.D., Penn L.Z. (2001). Blocking protein geranylgeranylation is essential for lovastatin-induced apoptosis of human acute myeloid leukemia cells. Leukemia.

[295] de Jonge-Peeters S.D., van der Weide K., Kuipers F., Sluiter W.J., de Vries E.G., Vellenga E. (2009). Variability in responsiveness to lovastatin of the primitive CD34+ AML subfraction compared to normal CD34+ cells. Ann. Hematol..

[296] Lee J.S., Roberts A., Juarez D., Vo T.T., Bhatt S., Herzog L.O., Mallya S., Bellin R.J., Agarwal S.K., Salem A.H. (2018). Statins enhance efficacy of venetoclax in blood cancers. Sci. Transl. Med..

[297] Shadman M., Mawad R., Dean C., Chen T.L., Shannon-Dorcy K., Sandhu V., Hendrie P.C., Scott B.L., Walter R.B., Becker P.S. (2015). Idarubicin, cytarabine, and pravastatin as induction therapy for untreated acute myeloid leukemia and high-risk myelodysplastic syndrome. Am. J. Hematol..

[298] Cunha Júnior A.D., Pericole F.V., Carvalheira J.B.C. (2018). Metformin and blood cancers. Clinics.

[299] Green A.S., Chapuis N., Maciel T.T., Willems L., Lambert M., Arnoult C., Boyer O., Bardet V., Park S., Foretz M. (2010). The LKB1/AMPK signaling pathway has tumor suppressor activity in acute myeloid leukemia through the repression of mTOR-dependent oncogenic mRNA translation. Blood.

[300] Skrtić M., Sriskanthadevan S., Jhas B., Gebbia M., Wang X., Wang Z., Hurren R., Jitkova Y., Gronda M., Maclean N. (2011). Inhibition of mitochondrial translation as a therapeutic strategy for human acute myeloid leukemia. Cancer Cell.

[301] Zhou X., Kuang Y., Liang S., Wang L. (2019). Metformin inhibits cell proliferation in SKM-1 cells via AMPK-mediated cell cycle arrest. J. Pharmacol. Sci..

[302] Liu L., Patnana P.K., Xie X., Frank D., Nimmagadda S.C., Rosemann A., Liebmann M., Klotz L., Opalka B., Khandanpour C. (2022). High Metabolic Dependence on Oxidative Phosphorylation Drives Sensitivity to Metformin Treatment in MLL/AF9 Acute Myeloid Leukemia. Cancers.

[303] Zhang Y., Li J., Shi W. (2022). Metformin Inhibits Acute Myeloid Leukemia Cells Growth through the AMPK/mTOR Pathway and Autophagic Regulation. Blood.

[304] Scotland C., Micklow E., Wang Z., Boutzen H., Recher C., Danet-Desnoyers G., Selak M., Carroll M., Sarry J.E. (2010). Metformin for Therapeutic Intervention In Acute Myeloid Leukemia. Blood.

[305] Huai L., Wang C., Zhang C., Li Q., Chen Y., Jia Y., Li Y., Xing H., Tian Z., Rao Q. (2012). Metformin induces differentiation in acute promyelocytic leukemia by activating the MEK/ERK signaling pathway. Biochem. Biophys. Res. Commun..

[306] Yuan F., Cheng C., Xiao F., Liu H., Cao S., Zhou G. (2020). Inhibition of mTORC1/P70S6K pathway by Metformin synergistically sensitizes Acute Myeloid Leukemia to Ara-C. Life Sci..

[307] Allende-Vega N., Marco Brualla J., Falvo P., Alexia C., Constantinides M., Fayd’herbe de Maudave A., Coenon L., Gitenay D., Mitola G., Massa P. (2022). Metformin sensitizes leukemic cells to cytotoxic lymphocytes by increasing expression of intercellular adhesion molecule-1 (ICAM-1). Sci. Rep..

[308] You R., Wang B., Chen P., Zheng X., Hou D., Wang X., Zhang B., Chen L., Li D., Lin X. (2022). Metformin sensitizes AML cells to chemotherapy through blocking mitochondrial transfer from stromal cells to AML cells. Cancer Lett..

[309] Valiulienė G., Vitkevičienė A., Skliutė G., Borutinskaitė V., Navakauskienė R. (2021). Pharmaceutical Drug Metformin and MCL1 Inhibitor S63845 Exhibit Anticancer Activity in Myeloid Leukemia Cells via Redox Remodeling. Molecules.

[310] Sabnis H.S., Bradley H.L., Tripathi S., Yu W.M., Tse W., Qu C.K., Bunting K.D. (2016). Synergistic cell death in FLT3-ITD positive acute myeloid leukemia by combined treatment with metformin and 6-benzylthioinosine. Leuk. Res..

[311] Asik A., Kayabasi C., Ozmen Yelken B., Yılmaz Susluer S., Dogan Sigva Z.O., Balcı Okcanoglu T., Saydam G., Biray Avci C., Gunduz C. (2018). Antileukemic effect of paclitaxel in combination with metformin in HL-60 cell line. Gene.

[312] Vitkevičienė A., Janulis V., Žučenka A., Borutinskaitė V., Kaupinis A., Valius M., Griškevičius L., Navakauskienė R. (2019). Oxidative phosphorylation inhibition induces anticancerous changes in therapy-resistant-acute myeloid leukemia patient cells. Mol. Carcinog..

[313] Zhou F.J., Zeng C.X., Kuang W., Cheng C., Liu H.C., Yan X.Y., Chen X.P., Zhou G., Cao S. (2021). Metformin exerts a synergistic effect with venetoclax by downregulating Mcl-1 protein in acute myeloid leukemia. J. Cancer.

[314] Chen M., Shen C., Chen Y., Chen Z., Zhou K., Chen Y., Li W., Zeng C., Qing Y., Wu D. (2024). Metformin synergizes with gilteritinib in treating FLT3-mutated leukemia via targeting PLK1 signaling. Cell Rep. Med..

[315] Krastinaite I., Charkavliuk S., Navakauskiene R., Borutinskaite V.V. (2024). Metformin as an Enhancer for the Treatment of Chemoresistant CD34+ Acute Myeloid Leukemia Cells. Genes.

[316] Allen L., Meck R., Yunis A. (1980). The inhibition of gamma-glutamyl transpeptidase from human pancreatic carcinoma cells by (alpha S,5S)-alpha-amino-3-chloro-4,5-dihydro-5-isoxazoleacetic acid (AT-125; NSC-163501). Res. Commun. Chem. Pathol. Pharmacol..

[317] Morell A., Losa G., Carrel S., Heumann D., von Fliedner V.E. (1986). Determination of ectoenzyme activities in leukemic cells and in established hematopoietic cell lines. Am. J. Hematol..

[318] Nichols K.E., Chitneni S.R., Moore J.O., Weinberg J.B. (1989). Monocytoid differentiation of freshly isolated human myeloid leukemia cells and HL-60 cells induced by the glutamine antagonist acivicin. Blood.

[319] Weinberg J.B., Mason S.N. (1991). Relationship of acivicin-induced monocytoid differentiation of human myeloid leukemia cells to acivicin-induced modulation of growth factor, cytokine, and protooncogene mRNA expression. Cancer Res..

[320] Bauvois B., Laouar A., Rouillard D., Wietzerbin J. (1995). Inhibition of gamma-glutamyl transpeptidase activity at the surface of human myeloid cells is correlated with macrophage maturation and transforming growth factor beta production. Cell Growth Differ..

[321] Bauvois B. (1997). In vitro effects of gamma-glutamyltranspeptidase inhibitor acivicin on human myeloid and B lineage cells. Adv. Exp. Med. Biol..

[322] del Bello B., Paolicchi A., Comporti M., Pompella A., Maellaro E. (1999). Hydrogen peroxide produced during gamma-glutamyl transpeptidase activity is involved in prevention of apoptosis and maintainance of proliferation in U937 cells. FASEB J..

[323] Losa G.A., Leoni L., Grob J.P. (1993). Enzymes transducing extracellular signals in acute myeloid human leukemias. Schweiz. Med. Wochenschr..

[324] Rzymowska J. (1995). Activities of enzyme transducing extracellular signals--gamma glutamyltransferase and enzymes metabolizing glutathione in acute lymphoblastic and myeloid human leukemias. Neoplasma.

[325] Ahmed N., Weidemann M.J. (1995). Biochemical effect of three different inhibitors of purine/pyrimidine metabolism on differentiation in HL60 cells. Leuk. Res..

[326] Antczak C., Karp D.R., London R.E., Bauvois B. (2001). Reanalysis of the involvement of gamma-glutamyl transpeptidase in the cell activation process. FEBS Lett..

[327] O’Dwyer P.J., Alonso M.T., Leyland-Jones B. (1984). Acivicin: A new glutamine antagonist in clinical trials. J. Clin. Oncol..

[328] Hidalgo M., Rodriguez G., Kuhn J.G., Brown T., Weiss G., MacGovren J.P., Von Hoff D.D., Rowinsky E.K. (1998). A Phase I and pharmacological study of the glutamine antagonist acivicin with the amino acid solution aminosyn in patients with advanced solid malignancies. Clin. Cancer Res..

[329] Antczak C., Bauvois B., Monneret C., Florent J.C. (2001). Targeting of acivicin prodrugs as antibody conjugates. J. Control. Release.

[330] Antczak C., Bauvois B., Monneret C., Florent J.C. (2001). A new acivicin prodrug designed for tumor-targeted delivery. Bioorg. Med. Chem..

[331] Madlener S., Illmer C., Horvath Z., Saiko P., Losert A., Herbacek I., Grusch M., Elford H.L., Krupitza G., Bernhaus A. (2007). Gallic acid inhibits ribonucleotide reductase and cyclooxygenases in human HL-60 promyelocytic leukemia cells. Cancer Lett..

[332] Iravani Saadi M., Moayedi J., Hosseini F., Rostamipour H.A., Karimi Z., Rahimian Z., Ahmadyan M., Ghahramani Z., Dehghani M., Yousefi K. (2024). The Effects of Resveratrol, Gallic Acid, and Piperine on the Expression of miR-17, miR-92b, miR-181a, miR-222, BAX, BCL-2, MCL-1, WT1, c-Kit, and CEBPA in Human Acute Myeloid Leukemia Cells and Their Roles in Apoptosis. Biochem. Genet..

[333] Xiang W., Sng C., Lam Y.H., Kok Z.H., Linn Y.C., Neo S.Y., Siew Y.Y., Singh D., Koh H.L., Chuah C. (2024). Gallic Acid Enhances the Efficacy of BCR::ABL1 Tyrosine Kinase Inhibitors in Chronic Myeloid Leukemia through Inhibition of Mitochondrial Respiration and Modulation of Oncogenic Signaling Pathways. Int. J. Mol. Sci..

[334] Reddy T.C., Bharat Reddy D., Aparna A., Arunasree K.M., Gupta G., Achari C., Reddy G.V., Lakshmipathi V., Subramanyam A., Reddanna P. (2012). Anti-leukemic effects of gallic acid on human leukemia K562 cells: Downregulation of COX-2, inhibition of BCR/ABL kinase and NF-κB inactivation. Toxicol. In Vitro.

[335] Gu R., Zhang M., Meng H., Xu D., Xie Y. (2018). Gallic acid targets acute myeloid leukemia via Akt/mTOR-dependent mitochondrial respiration inhibition. Biomed. Pharmacother..

[336] Chen Y.J., Chang L.S. (2012). Gallic acid downregulates matrix metalloproteinase-2 (MMP-2) and MMP-9 in human leukemia cells with expressed Bcr/Abl. Mol. Nutr. Food Res..

[337] Asano Y., Okamura S., Ogo T., Eto T., Otsuka T., Niho Y. (1997). Effect of (-)-epigallocatechin gallate on leukemic blast cells from patients with acute myeloblastic leukemia. Life Sci..

[338] Ly B.T., Chi H.T., Yamagishi M., Kano Y., Hara Y., Nakano K., Sato Y., Watanabe T. (2013). Inhibition of FLT3 expression by green tea catechins in FLT3 mutated-AML cells. PLoS ONE.

[339] Han D.H., Jeong J.H., Kim J.H. (2009). Anti-proliferative and apoptosis induction activity of green tea polyphenols on human promyelocytic leukemia HL-60 cells. Anticancer Res..

[340] Britschgi A., Simon H.U., Tobler A., Fey M.F., Tschan M.P. (2010). Epigallocatechin-3-gallate induces cell death in acute myeloid leukaemia cells and supports all-trans retinoic acid-induced neutrophil differentiation via death-associated protein kinase 2. Br. J. Haematol..

[341] Borutinskaitė V., Virkšaitė A., Gudelytė G., Navakauskienė R. (2018). Green tea polyphenol EGCG causes anti-cancerous epigenetic modulations in acute promyelocytic leukemia cells. Leuk. Lymphoma.

[342] Della Via F.I., Shiraishi R.N., Santos I., Ferro K.P., Salazar-Terreros M.J., Franchi Junior G.C., Rego E.M., Saad S.T.O., Torello C.O. (2021). (-)-Epigallocatechin-3-gallate induces apoptosis and differentiation in leukaemia by targeting reactive oxygen species and PIN1. Sci. Rep..

[343] Nakazato T., Ito K., Miyakawa Y., Kinjo K., Yamada T., Hozumi N., Ikeda Y., Kizaki M. (2005). Catechin, a green tea component, rapidly induces apoptosis of myeloid leukemic cells via modulation of reactive oxygen species production in vitro and inhibits tumor growth in vivo. Haematologica.

[344] Ly B.T.K., Chi H.T. (2020). Combined effect of (-)-epigallocatechin-3-gallate and all-trans retinoic acid in FLT3-mutated cell lines. Biomed. Rep..

[345] Jokar M.H., Sedighi S., Moradzadeh M. (2021). A comparative study of anti-leukemic effects of kaempferol and epigallocatechin-3-gallate (EGCG) on human leukemia HL-60 cells. Avicenna J. Phytomed..

[346] Bae K.H., Lai F., Oruc B., Osato M., Chen Q., Kurisawa M. (2022). Self-Assembled Daunorubicin/Epigallocatechin Gallate Nanocomplex for Synergistic Reversal of Chemoresistance in Leukemia. Int. J. Mol. Sci..

[347] Bae K.H., Lai F., Mong J., Niibori-Nambu A., Chan K.H., Her Z., Osato M., Tan M.H., Chen Q., Kurisawa M. (2022). Bone marrow-targetable Green Tea Catechin-Based Micellar Nanocomplex for synergistic therapy of Acute myeloid leukemia. J. Nanobiotechnol..

[348] Liang K., Bae K.H., Nambu A., Dutta B., Chung J.E., Osato M., Kurisawa M. (2019). A two-pronged anti-leukemic agent based on a hyaluronic acid-green tea catechin conjugate for inducing targeted cell death and terminal differentiation. Biomater. Sci..

[349] Pan R., Ruvolo V.R., Wei J., Konopleva M., Reed J.C., Pellecchia M., Andreeff M., Ruvolo P.P. (2015). Inhibition of Mcl-1 with the pan-Bcl-2 family inhibitor (-)BI97D6 overcomes ABT-737 resistance in acute myeloid leukemia. Blood.

[350] Zhang L., Zhou Y., Chen K., Shi P., Li Y., Deng M., Jiang Z., Wang X., Li P., Xu B. (2017). The pan-Bcl2 Inhibitor AT101 Activates the Intrinsic Apoptotic Pathway and Causes DNA Damage in Acute Myeloid Leukemia Stem-Like Cells. Target. Oncol..

[351] Yang Q., Chen K., Zhang L., Feng L., Fu G., Jiang S., Bi S., Lin C., Zhou Y., Zhao H. (2019). Synthetic lethality of combined AT-101 with idarubicin in acute myeloid leukemia via blockade of DNA repair and activation of intrinsic apoptotic pathway. Cancer Lett..

[352] Ai C.J., Chen L.J., Guo L.X., Wang Y.P., Zhao Z.Y. (2024). Gossypol acetic acid regulates leukemia stem cells by degrading LRPPRC via inhibiting IL-6/JAK1/STAT3 signaling or resulting mitochondrial dysfunction. World J. Stem Cells.

[353] Jang M., Cai L., Udeani G.O., Slowing K.V., Thomas C.F., Beecher C.W., Fong H.H., Farnsworth N.R., Kinghorn A.D., Mehta R.G. (1997). Cancer chemopreventive activity of resveratrol, a natural product derived from grapes. Science.

[354] Surh Y.J., Hurh Y.J., Kang J.Y., Lee E., Kong G., Lee S.J. (1999). Resveratrol, an antioxidant present in red wine, induces apoptosis in human promyelocytic leukemia (HL-60) cells. Cancer Lett..

[355] Park J.W., Choi Y.J., Jang M.A., Lee Y.S., Jun D.Y., Suh S.I., Baek W.K., Suh M.H., Jin I.N., Kwon T.K. (2001). Chemopreventive agent resveratrol, a natural product derived from grapes, reversibly inhibits progression through S and G2 phases of the cell cycle in U937 cells. Cancer Lett..

[356] Ferry-Dumazet H., Garnier O., Mamani-Matsuda M., Vercauteren J., Belloc F., Billiard C., Dupouy M., Thiolat D., Kolb J.P., Marit G. (2002). Resveratrol inhibits the growth and induces the apoptosis of both normal and leukemic hematopoietic cells. Carcinogenesis.

[357] Yaseen A., Chen S., Hock S., Rosato R., Dent P., Dai Y., Grant S. (2012). Resveratrol sensitizes acute myelogenous leukemia cells to histone deacetylase inhibitors through reactive oxygen species-mediated activation of the extrinsic apoptotic pathway. Mol. Pharmacol..

[358] Tsan M.F., White J.E., Maheshwari J.G., Chikkappa G. (2002). Anti-leukemia effect of resveratrol. Leuk. Lymphoma.

[359] Estrov Z., Shishodia S., Faderl S., Harris D., Van Q., Kantarjian H.M., Talpaz M., Aggarwal B.B. (2003). Resveratrol blocks interleukin-1beta-induced activation of the nuclear transcription factor NF-kappaB, inhibits proliferation, causes S-phase arrest, and induces apoptosis of acute myeloid leukemia cells. Blood.

[360] Anuchapreedr S., Sadjapong W., Duangratb C., Limtrakul P. (2006). The cytotoxic effect of curcumin, demethoxycurcumin and bisdemethoxycurcumin purified from Turmeric powder on leukemic cell lines. Bull. Chisng Mti Assoc. Mcd Sci..

[361] Zhang J.R., Lu F., Lu T., Dong W.H., Li P., Liu N., Ma D.X., Ji C.Y. (2014). Inactivation of FoxM1 transcription factor contributes to curcumin-induced inhibition of survival, angiogenesis, and chemosensitivity in acute myeloid leukemia cells. J. Mol. Med..

[362] Nagy L.I., Fehér L.Z., Szebeni G.J., Gyuris M., Sipos P., Alföldi R., Ózsvári B., Hackler L., Balázs A., Batár P. (2015). Curcumin and its analogue induce apoptosis in leukemia cells and have additive effects with bortezomib in cellular and xenograft models. BioMed Res. Int..

[363] Zeng Y., Weng G., Fan J., Li Z., Wu J., Li Y., Zheng R., Xia P., Guo K. (2016). Curcumin reduces the expression of survivin, leading to enhancement of arsenic trioxide-induced apoptosis in myelodysplastic syndrome and leukemia stem-like cells. Oncol. Rep..

[364] Salemi M., Mohammadi S., Ghavamzadeh A., Nikbakht M. (2017). Anti-Vascular Endothelial Growth Factor Targeting by Curcumin and Thalidomide in Acute Myeloid Leukemia Cells. Asian Pac. J. Cancer Prev..

[365] Martínez-Castillo M., Villegas-Sepúlveda N., Meraz-Rios M.A., Hernández-Zavala A., Berumen J., Coleman M.A., Orozco L., Cordova E.J. (2018). Curcumin differentially affects cell cycle and cell death in acute and chronic myeloid leukemia cells. Oncol. Lett..

[366] Zhu G., Shen Q., Jiang H., Ji O., Zhu L., Zhang L. (2020). Curcumin inhibited the growth and invasion of human monocytic leukaemia SHI-1 cells in vivo by altering MAPK and MMP signalling. Pharm. Biol..

[367] Martín I., Navarro B., Solano C., Calabuig M., Hernández-Boluda J.C., Amat P., Remigia M.J., García F., Villamón E., Tormo M. (2019). Synergistic Antioncogenic Activity of Azacitidine and Curcumin in Myeloid Leukemia Cell Lines and Patient Samples. Anticancer Res..

[368] Rao J., Xu D.R., Zheng F.M., Long Z.J., Huang S.S., Wu X., Zhou W.H., Huang R.W., Liu Q. (2011). Curcumin reduces expression of Bcl-2, leading to apoptosis in daunorubicin-insensitive CD34+ acute myeloid leukemia cell lines and primary sorted CD34+ acute myeloid leukemia cells. J. Transl. Med..

[369] Yu J., Peng Y., Wu L.C., Xie Z., Deng Y., Hughes T., He S., Mo X., Chiu M., Wang Q.E. (2013). Curcumin down-regulates DNA methyltransferase 1 and plays an anti-leukemic role in acute myeloid leukemia. PLoS ONE.

[370] Chueahongthong F., Tima S., Chiampanichayakul S., Berkland C., Anuchapreeda S. (2021). Co-Treatments of Edible Curcumin from Turmeric Rhizomes and Chemotherapeutic Drugs on Cytotoxicity and FLT3 Protein Expression in Leukemic Stem Cells. Molecules.

[371] Zhu G.H., Dai H.P., Shen Q., Ji O., Zhang Q., Zhai Y.L. (2016). Curcumin induces apoptosis and suppresses invasion through MAPK and MMP signaling in human monocytic leukemia SHI-1 cells. Pharm. Biol..

[372] Zhou H., Ning Y., Zeng G., Zhou C., Ding X. (2021). Curcumin promotes cell cycle arrest and apoptosis of acute myeloid leukemia cells by inactivating AKT. Oncol. Rep..

[373] Liu J.M., Li M., Luo W., Sun H.B. (2021). Curcumin attenuates Adriamycin-resistance of acute myeloid leukemia by inhibiting the lncRNA HOTAIR/miR-20a-5p/WT1 axis. Lab. Investig..

[374] Chow J.M., Huang G.C., Shen S.C., Wu C.Y., Lin C.W., Chen Y.C. (2008). Differential apoptotic effect of wogonin and nor-wogonin via stimulation of ROS production in human leukemia cells. J. Cell. Biochem..

[375] Huang S.T., Wang C.Y., Yang R.C., Chu C.J., Wu H.T., Pang J.H. (2010). Wogonin, an active compound in Scutellaria baicalensis, induces apoptosis and reduces telomerase activity in the HL-60 leukemia cells. Phytomedicine.

[376] Hu C., Xu M., Qin R., Chen W., Xu X. (2015). Wogonin induces apoptosis and endoplasmic reticulum stress in HL-60 leukemia cells through inhibition of the PI3K-AKT signaling pathway. Oncol. Rep..

[377] Yu X., Li H., Hu P., Qing Y., Wang X., Zhu M., Wang H., Wang Z., Xu J., Guo Q. (2020). Natural HDAC-1/8 inhibitor baicalein exerts therapeutic effect in CBF-AML. Clin. Transl. Med..

[378] Li Q., Ren J. (2024). Baicalein Promotes Acute Myeloid Leukemia Cell Autophagy via miR-424 and the PTEN/PI3K/AKT/mTOR Pathway. Lett. Drug Des. Discov..

[379] Wang I.K., Lin-Shiau S.Y., Lin J.K. (1999). Induction of apoptosis by apigenin and related flavonoids through cytochrome c release and activation of caspase-9 and caspase-3 in leukaemia HL-60 cells. Eur. J. Cancer.

[380] Vargo M.A., Voss O.H., Poustka F., Cardounel A.J., Grotewold E., Doseff A.I. (2006). Apigenin-induced-apoptosis is mediated by the activation of PKCdelta and caspases in leukemia cells. Biochem. Pharmacol..

[381] Ruela-de-Sousa R.R., Fuhler G.M., Blom N., Ferreira C.V., Aoyama H., Peppelenbosch M.P. (2010). Cytotoxicity of apigenin on leukemia cell lines: Implications for prevention and therapy. Cell Death Dis..

[382] Gonzalez-Mejia M.E., Voss O.H., Murnan E.J., Doseff A.I. (2010). Apigenin-induced apoptosis of leukemia cells is mediated by a bimodal and differentially regulated residue-specific phosphorylation of heat-shock protein-27. Cell Death Dis..

[383] Budhraja A., Gao N., Zhang Z., Son Y.O., Cheng S., Wang X., Ding S., Hitron A., Chen G., Luo J. (2012). Apigenin induces apoptosis in human leukemia cells and exhibits anti-leukemic activity in vivo. Mol. Cancer Ther..

[384] Mahbub A.A., Le Maitre C.L., Haywood-Small S.L., McDougall G.J., Cross N.A., Jordan-Mahy N. (2013). Differential effects of polyphenols on proliferation and apoptosis in human myeloid and lymphoid leukemia cell lines. Anti-Cancer Agents Med. Chem..

[385] Mahbub A.A., Le Maitre C.L., Haywood-Small S.L., Cross N.A., Jordan-Mahy N. (2015). Polyphenols act synergistically with doxorubicin and etoposide in leukaemia cell lines. Cell Death Discov..

[386] Mahbub A.A., Maitre C.L.L., Haywood-Small S., Cross N.A., Jordan-Mahy N. (2019). Polyphenols enhance the activity of alkylating agents in leukaemia cell lines. Oncotarget.

[387] Mahbub A.A., Le Maitre C.L., Cross N.A., Jordan-Mahy N. (2022). The effect of apigenin and chemotherapy combination treatments on apoptosis-related genes and proteins in acute leukaemia cell lines. Sci. Rep..

[388] Cheng S., Gao N., Zhang Z., Chen G., Budhraja A., Ke Z., Son Y.O., Wang X., Luo J., Shi X. (2010). Quercetin induces tumor-selective apoptosis through downregulation of Mcl-1 and activation of Bax. Clin. Cancer Res..

[389] Yuan Z., Long C., Junming T., Qihuan L., Youshun Z., Chan Z. (2012). Quercetin-induced apoptosis of HL-60 cells by reducing PI3K/Akt. Mol. Biol. Rep..

[390] Lee W.J., Hsiao M., Chang J.L., Yang S.F., Tseng T.H., Cheng C.W., Chow J.M., Lin K.H., Lin Y.W., Liu C.C. (2015). Quercetin induces mitochondrial-derived apoptosis via reactive oxygen species-mediated ERK activation in HL-60 leukemia cells and xenograft. Arch. Toxicol..

[391] Shi H., Li X.Y., Chen Y., Zhang X., Wu Y., Wang Z.X., Chen P.H., Dai H.Q., Feng J., Chatterjee S. (2020). Quercetin Induces Apoptosis via Downregulation of Vascular Endothelial Growth Factor/Akt Signaling Pathway in Acute Myeloid Leukemia Cells. Front. Pharmacol..

[392] Alvarez M.C., Maso V., Torello C.O., Ferro K.P., Saad S.T.O. (2018). The polyphenol quercetin induces cell death in leukemia by targeting epigenetic regulators of pro-apoptotic genes. Clin. Epigenetics.

[393] Larocca L.M., Teofili L., Leone G., Sica S., Pierelli L., Menichella G., Scambia G., Benedetti Panici P., Ricci R., Piantelli M. (1991). Antiproliferative activity of quercetin on normal bone marrow and leukaemic progenitors. Br. J. Haematol..

[394] Larocca L.M., Teofili L., Sica S., Piantelli M., Maggiano N., Leone G., Ranelletti F.O. (1995). Quercetin inhibits the growth of leukemic progenitors and induces the expression of transforming growth factor-beta 1 in these cells. Blood.

[395] Sun J., Sha M., Zhou J., Huang Y. (2025). Quercetin affects apoptosis and autophagy in pediatric acute myeloid leukaemia cells by inhibiting PI3K/AKT signaling pathway activation through regulation of miR-224-3p/PTEN axis. BMC Cancer.

[396] Calgarotto A.K., Maso V., Junior G.C.F., Nowill A.E., Filho P.L., Vassallo J., Saad S.T.O. (2018). Antitumor activities of Quercetin and Green Tea in xenografts of human leukemia HL60 cells. Sci. Rep..

[397] Kawakatsu R., Tadagaki K., Yamasaki K., Kuwahara Y., Nakada S., Yoshida T. (2024). The combination of venetoclax and quercetin exerts a cytotoxic effect on acute myeloid leukemia. Sci. Rep..

[398] Xiao J., Zhang B., Yin S., Xie S., Huang K., Wang J., Yang W., Liu H., Zhang G., Liu X. (2022). Quercetin induces autophagy-associated death in HL-60 cells through CaMKKβ/AMPK/mTOR signal pathway. Acta Biochim. Biophys. Sin..

[399] Marfe G., Tafani M., Indelicato M., Sinibaldi-Salimei P., Reali V., Pucci B., Fini M., Russo M.A. (2009). Kaempferol induces apoptosis in two different cell lines via Akt inactivation, Bax and SIRT3 activation, and mitochondrial dysfunction. J. Cell. Biochem..

[400] Park J.H., Jin C.Y., Lee B.K., Kim G.Y., Choi Y.H., Jeong Y.K. (2008). Naringenin induces apoptosis through downregulation of Akt and caspase-3 activation in human leukemia THP-1 cells. Food Chem. Toxicol..

[401] Wen C., Lu X., Sun Y., Li Q., Liao J., Li L. (2023). Naringenin induces the cell apoptosis of acute myeloid leukemia cells by regulating the lncRNA XIST/miR-34a/HDAC1 signaling. Heliyon.

[402] Shi D., Xu Y., Du X., Chen X., Zhang X., Lou J., Li M., Zhuo J. (2015). Co-treatment of THP-1 cells with naringenin and curcumin induces cell cycle arrest and apoptosis via numerous pathways. Mol. Med. Rep..

[403] Hsiao P.C., Lee W.J., Yang S.F., Tan P., Chen H.Y., Lee L.M., Chang J.L., Lai G.M., Chow J.M., Chien M.H. (2014). Nobiletin suppresses the proliferation and induces apoptosis involving MAPKs and caspase-8/-9/-3 signals in human acute myeloid leukemia cells. Tumour Biol..

[404] Park C., Lee W.S., Go S.I., Nagappan A., Han M.H., Hong S.H., Kim G.S., Kim G.Y., Kwon T.K., Ryu C.H. (2014). Morin, a flavonoid from moraceae, induces apoptosis by induction of BAD protein in human leukemic cells. Int. J. Mol. Sci..

[405] Gao H., Liu Y., Li K., Wu T., Peng J., Jing F. (2016). Hispidulin induces mitochondrial apoptosis in acute myeloid leukemia cells by targeting extracellular matrix metalloproteinase inducer. Am. J. Transl. Res..

[406] Chen J., Cai Y.F., Shao M., Cong H. (2023). Effect of Scutellarin on Proliferation of Acute Myeloid Leukemia Cells and Its Related Mechanism. Zhongguo Shi Yan Xue Ye Xue Za Zhi.

[407] Parsa L., Motafakkerazad R., Soheyli S.T., Haratian A., Kosari-Nasab M., Mahdavi M. (2023). Silymarin in combination with ATRA enhances apoptosis induction in human acute promyelocytic NB4 cells. Toxicon.

[408] Bauvois B., Puiffe M.L., Bongui J.B., Paillat S., Monneret C., Dauzonne D. (2003). Synthesis and biological evaluation of novel flavone-8-acetic acid derivatives as reversible inhibitors of aminopeptidase N/CD13. J. Med. Chem..

[409] Bouchet S., Tang R., Fava F., Legrand O., Bauvois B. (2016). The CNGRC-GG-D(KLAKLAK)2 peptide induces a caspase-independent, Ca2+-dependent death in human leukemic myeloid cells by targeting surface aminopeptidase N/CD13. Oncotarget.

[410] Piedfer M., Bouchet S., Tang R., Billard C., Dauzonne D., Bauvois B. (2013). p70S6 kinase is a target of the novel proteasome inhibitor 3,3′-diamino-4′-methoxyflavone during apoptosis in human myeloid tumor cells. Biochim. Biophys. Acta.

[411] Cárdenas M.G., Blank V.C., Marder M.N., Roguin L.P. (2012). 2′-Nitroflavone induces apoptosis and modulates mitogen-activated protein kinase pathways in human leukaemia cells. Anticancer Drugs.

[412] Chang H., Mi M., Ling W., Zhu J., Zhang Q., Wei N., Zhou Y., Tang Y., Yuan J. (2008). Structurally related cytotoxic effects of flavonoids on human cancer cells in vitro. Arch. Pharmacal Res..

[413] Chang H., Lin H., Yi L., Zhu J., Zhou Y., Mi M., Zhang Q. (2010). 3,6-Dihydroxyflavone induces apoptosis in leukemia HL-60 cell via reactive oxygen species-mediated p38 MAPK/JNK pathway. Eur. J. Pharmacol..

[414] Jeyaraju D.V., Hurren R., Wang X., MacLean N., Gronda M., Shamas-Din A., Minden M.D., Giaever G., Schimmer A.D. (2016). A novel isoflavone, ME-344, targets the cytoskeleton in acute myeloid leukemia. Oncotarget.

[415] Hurrish K.H., Su Y., Patel S., Ramage C.L., Carter J.L., Edwards H., Buck S.A., Wiley S.E., Hüttemann M., Polin L. (2023). Enhancing anti-AML activity of venetoclax by isoflavone ME-344 through suppression of OXPHOS and/or purine biosynthesis. Res. Sq..

[416] Kwon K.B., Yoo S.J., Ryu D.G., Yang J.Y., Rho H.W., Kim J.S., Park J.W., Kim H.R., Park B.H. (2002). Induction of apoptosis by diallyl disulfide through activation of caspase-3 in human leukemia HL-60 cells. Biochem. Pharmacol..

[417] Lin J.G., Chen G.W., Su C.C., Hung C.F., Yang C.C., Lee J.H., Chung J.G. (2002). Effects of garlic components diallyl sulfide and diallyl disulfide on arylamine N-acetyltransferase activity and 2-aminofluorene-DNA adducts in human promyelocytic leukemia cells. Am. J. Chin. Med..

[418] Tan H., Ling H., He J., Yi L., Zhou J., Lin M., Su Q. (2008). Inhibition of ERK and activation of p38 are involved in diallyl disulfide induced apoptosis of leukemia HL-60 cells. Arch. Pharmacal Res..

[419] Dasgupta P., Bandyopadhyay S.S. (2013). Role of di-allyl disulfide, a garlic component in NF-κB mediated transient G2-M phase arrest and apoptosis in human leukemic cell-lines. Nutr. Cancer.

[420] Agassi S.F.T., Yeh T.M., Chang C.D., Hsu J.L., Shih W.L. (2020). Potentiation of Differentiation and Apoptosis in a Human Promyelocytic Leukemia Cell Line by Garlic Essential Oil and Its Organosulfur Compounds. Anticancer Res..

[421] Zheng S., Yang H., Zhang S., Wang X., Yu L., Lu J., Li J. (1997). Initial study on naturally occurring products from traditional Chinese herbs and vegetables for chemoprevention. J. Cell Biochem. Suppl..

[422] Cerella C., Scherer C., Cristofanon S., Henry E., Anwar A., Busch C., Montenarh M., Dicato M., Jacob C., Diederich M. (2009). Cell cycle arrest in early mitosis and induction of caspase-dependent apoptosis in U937 cells by diallyltetrasulfide (Al2S4). Apoptosis.

[423] Ahmed N., Laverick L., Sammons J., Zhang H., Maslin D.J., Hassan H.T. (2001). Ajoene, a garlic-derived natural compound, enhances chemotherapy-induced apoptosis in human myeloid leukaemia CD34-positive resistant cells. Anticancer Res..

[424] Xu B., Monsarrat B., Gairin J.E., Girbal-Neuhauser E. (2004). Effect of ajoene, a natural antitumor small molecule, on human 20S proteasome activity in vitro and in human leukemic HL60 cells. Fundam. Clin. Pharmacol..

[425] Hassan H.T. (2004). Ajoene (natural garlic compound): A new anti-leukaemia agent for AML therapy. Leuk. Res..

[426] Merhi F., Auger J., Rendu F., Bauvois B. (2008). Allium compounds, dipropyl and dimethyl thiosulfinates as antiproliferative and differentiating agents of human acute myeloid leukemia cell lines. Biologics.

[427] Zhang K., Li J., Meng W., Xing H., Yang Y. (2010). C/EBPβ and CHOP participate in tanshinone IIA-induced differentiation and apoptosis of acute promyelocytic leukemia cells in vitro. Int. J. Hematol..

[428] Oh J.H., Lee J.T., Yang E.S., Chang J.S., Lee D.S., Kim S.H., Choi Y.H., Park J.W., Kwon T.K. (2009). The coffee diterpene kahweol induces apoptosis in human leukemia U937 cells through down-regulation of Akt phosphorylation and activation of JNK. Apoptosis.

[429] Liu C.X., Yin Q.Q., Zhou H.C., Wu Y.L., Pu J.X., Xia L., Liu W., Huang X., Jiang T., Wu M.X. (2012). Adenanthin targets peroxiredoxin I and II to induce differentiation of leukemic cells. Nat. Chem. Biol..

[430] Zhen T., Wu C.F., Liu P., Wu H.Y., Zhou G.B., Lu Y., Liu J.X., Liang Y., Li K.K., Wang Y.Y. (2012). Targeting of AML1-ETO in t(8;21) leukemia by oridonin generates a tumor suppressor-like protein. Sci. Transl. Med..

[431] Carter B.Z., Mak D.H., Schober W.D., Dietrich M.F., Pinilla C., Vassilev L.T., Reed J.C., Andreeff M. (2008). Triptolide sensitizes AML cells to TRAIL-induced apoptosis via decrease of XIAP and p53-mediated increase of DR5. Blood.

[432] Zhang T., He Y.M., Wang J.S., Shen J., Xing Y.Y., Xi T. (2011). Ursolic acid induces HL60 monocytic differentiation and upregulates C/EBPβ expression by ERK pathway activation. Anticancer Drugs.

[433] Hostanska K., Reichling J., Bommer S., Weber M., Saller R. (2003). Hyperforin a constituent of St John’s wort (Hypericum perforatum L.) extract induces apoptosis by triggering activation of caspases and with hypericin synergistically exerts cytotoxicity towards human malignant cell lines. Eur. J. Pharm. Biopharm..

[434] Liu J.Y., Liu Z., Wang D.M., Li M.M., Wang S.X., Wang R., Chen J.P., Wang Y.F., Yang D.P. (2011). Induction of apoptosis in K562 cells by dicyclohexylammonium salt of hyperforin through a mitochondrial-related pathway. Chem.-Biol. Interact..

[435] Merhi F., Tang R., Piedfer M., Mathieu J., Bombarda I., Zaher M., Kolb J.P., Billard C., Bauvois B. (2011). Hyperforin Inhibits Akt1 Kinase Activity and Promotes Caspase-mediated Apoptosis involving Bad and Noxa Activation in Human Myeloid Tumor Cells. PLoS ONE.

[436] Quiney C., Billard C., Faussat A.M., Salanoubat C., Kolb J.P. (2007). Hyperforin inhibits P-gp and BCRP activities in chronic lymphocytic leukaemia cells and myeloid cells. Leuk. Lymphoma.

[437] Skopek R., Palusińska M., Kaczor-Keller K., Pingwara R., Papierniak-Wyglądała A., Schenk T., Lewicki S., Zelent A., Szymański Ł. (2023). Choosing the Right Cell Line for Acute Myeloid Leukemia (AML) Research. Int. J. Mol. Sci..

[438] Chabot G.G., Touil Y.S., Pham M.H., Dauzonne D. (2010). Flavonoids in Cancer Prevention and Therapy: Chemistry, Pharmacology, Mechanisms of Action, and Perspectives for Cancer Drug Discovery. Altern. Complement. Ther. Cancer.

[439] Singla R.K., Dubey A.K., Garg A., Sharma R.K., Fiorino M., Ameen S.M., Haddad M.A., Al-Hiary M. (2019). Natural Polyphenols: Chemical Classification, Definition of Classes, Subcategories, and Structures. J. AOAC Int..

[440] Carrano R., Grande M., Leti Maggio E., Zucca C., Bei R., Palumbo C., Focaccetti C., Nardozi D., Lucarini V., Angiolini V. (2024). Dietary Polyphenols Effects on Focal Adhesion Plaques and Metalloproteinases in Cancer Invasiveness. Biomedicines.

[441] Dias M.C., Pinto D., Silva A.M.S. (2021). Plant Flavonoids: Chemical Characteristics and Biological Activity. Molecules.

[442] Liga S., Paul C., Péter F. (2023). Flavonoids: Overview of Biosynthesis, Biological Activity, and Current Extraction Techniques. Plants.

[443] Ashrafizadeh M., Zarrabi A., Mirzaei S., Hashemi F., Samarghandian S., Zabolian A., Hushmandi K., Ang H.L., Sethi G., Kumar A.P. (2021). Gallic acid for cancer therapy: Molecular mechanisms and boosting efficacy by nanoscopical delivery. Food Chem. Toxicol..

[444] Shao Y., Luo W., Guo Q., Li X., Zhang Q., Li J. (2019). In vitro and in vivo effect of hyaluronic acid modified, doxorubicin and gallic acid co-delivered lipid-polymeric hybrid nano-system for leukemia therapy. Drug Des. Dev. Ther..

[445] Tóth E., Erdődi F., Kiss A. (2021). Activation of Myosin Phosphatase by Epigallocatechin-Gallate Sensitizes THP-1 Leukemic Cells to Daunorubicin. Anti-Cancer Agents Med. Chem..

[446] Becattini B., Kitada S., Leone M., Monosov E., Chandler S., Zhai D., Kipps T.J., Reed J.C., Pellecchia M. (2004). Rational design and real time, in-cell detection of the proapoptotic activity of a novel compound targeting Bcl-X(L). Chem. Biol..

[447] Kitada S., Leone M., Sareth S., Zhai D., Reed J.C., Pellecchia M. (2003). Discovery, characterization, and structure-activity relationships studies of proapoptotic polyphenols targeting B-cell lymphocyte/leukemia-2 proteins. J. Med. Chem..

[448] Etxebarria A., Landeta O., Antonsson B., Basañez G. (2008). Regulation of antiapoptotic MCL-1 function by gossypol: Mechanistic insights from in vitro reconstituted systems. Biochem. Pharmacol..

[449] Swarn S., Alaseem A.M., Sharma S., Paria A., Prajapati B.G., Alasiri G., Gandhi S.M., Kapoor D.U. (2025). Gossypol-based nanocarriers for cancer treatment: Advances and future perspectives. J. Biomater. Sci. Polym. Ed..

[450] Sharma K., Kumar M., Dukare A., Vigneshwaran N., Thappa C., Saxena S., Pandiyan K., D’Souza C., Singh R. (2025). Gossypol and Semisynthetic Derivatives: Chemistry, Bioactivities, and Mechanism of Actions. Chem. Biodivers..

[451] Shaito A., Posadino A.M., Younes N., Hasan H., Halabi S., Alhababi D., Al-Mohannadi A., Abdel-Rahman W.M., Eid A.H., Nasrallah G.K. (2020). Potential Adverse Effects of Resveratrol: A Literature Review. Int. J. Mol. Sci..

[452] Ersöz N., Adan A. (2022). Resveratrol triggers anti-proliferative and apoptotic effects in FLT3-ITD-positive acute myeloid leukemia cells via inhibiting ceramide catabolism enzymes. Med. Oncol..

[453] Fulda S. (2010). Resveratrol and derivatives for the prevention and treatment of cancer. Drug Discov. Today.

[454] Horvath Z., Murias M., Saiko P., Erker T., Handler N., Madlener S., Jaeger W., Grusch M., Fritzer-Szekeres M., Krupitza G. (2006). Cytotoxic and biochemical effects of 3,3′,4,4′,5,5′-hexahydroxystilbene, a novel resveratrol analog in HL-60 human promyelocytic leukemia cells. Exp. Hematol..

[455] Howells L.M., Berry D.P., Elliott P.J., Jacobson E.W., Hoffmann E., Hegarty B., Brown K., Steward W.P., Gescher A.J. (2011). Phase I randomized, double-blind pilot study of micronized resveratrol (SRT501) in patients with hepatic metastases--safety, pharmacokinetics, and pharmacodynamics. Cancer Prev. Res..

[456] Salman U.-I., Ahmed M.B., Ul-Islam M., Shehzad A., Lee Y.S. (2019). Switching from Conventional to Nano-natural Phytochemicals to Prevent and Treat Cancers: Special Emphasis on Resveratrol. Curr. Pharm. Des..

[457] Santos A.C., Pereira I., Magalhães M., Pereira-Silva M., Caldas M., Ferreira L., Figueiras A., Ribeiro A.J., Veiga F. (2019). Targeting Cancer Via Resveratrol-Loaded Nanoparticles Administration: Focusing on In Vivo Evidence. AAPS J..

[458] Surapally S., Jayaprakasam M., Verma R.S. (2020). Curcumin augments therapeutic efficacy of TRAIL-based immunotoxins in leukemia. Pharmacol. Rep..

[459] Sun D., Zhou J.K., Zhao L., Zheng Z.Y., Li J., Pu W., Liu S., Liu X.S., Liu S.J., Zheng Y. (2017). Novel Curcumin Liposome Modified with Hyaluronan Targeting CD44 Plays an Anti-Leukemic Role in Acute Myeloid Leukemia in Vitro and in Vivo. ACS Appl. Mater. Interfaces.

[460] Amjad E., Sokouti B., Asnaashari S. (2022). A systematic review of anti-cancer roles and mechanisms of kaempferol as a natural compound. Cancer Cell Int..

[461] Banik K., Khatoon E., Harsha C., Rana V., Parama D., Thakur K.K., Bishayee A., Kunnumakkara A.B. (2022). Wogonin and its analogs for the prevention and treatment of cancer: A systematic review. Phytother. Res..

[462] Dauzonne D., Folléas B., Martinez L., Chabot G.G. (1997). Synthesis and in vitro cytotoxicity of a series of 3-aminoflavones. Eur. J. Med. Chem..

[463] Barros A., Silva A. (2006). Efficient Synthesis of Nitroflavones by Cyclodehydrogenation of 20-Hydroxychalcones and by the Baker-Venkataraman Method. Monatshefte Für Chem..

[464] Cardenas M., Marder M., Blank V.C., Roguin L.P. (2006). Antitumor activity of some natural flavonoids and synthetic derivatives on various human and murine cancer cell lines. Bioorg. Med. Chem..

[465] Zhang L., Zhang J., Ye Z., Townsend D.M., Tew K.D. (2019). Pharmacology of ME-344, a novel cytotoxic isoflavone. Adv. Cancer Res..

[466] Amagase H. (2006). Clarifying the real bioactive constituents of garlic. J. Nutr..

[467] Lanzotti V. (2006). The analysis of onion and garlic. J. Chromatogr. A.

[468] Khanam S., Mishra P., Faruqui T., Alam P., Albalawi T., Siddiqui F., Rafi Z., Khan S. (2025). Plant-based secondary metabolites as natural remedies: A comprehensive review on terpenes and their therapeutic applications. Front. Pharmacol..

[469] Li F., Gao C., Li X., Wang J., Zhao Y., Ke Y., Liu Y., Liu H.M., Hu Z., Wei L. (2022). Jiyuan oridonin A induces differentiation of acute myeloid leukemia cells including leukemic stem-like cells. Front. Pharmacol..

[470] Chen L., Li D., Guo X., Cheng C., Wei X. (2023). Oridonin Synergistically Enhances the Pro-Apoptotic Effect of Venetoclax on Acute Myeloid Leukemia Cells by Inhibiting AKT Signaling. Front. Biosci. (Landmark Ed.).

[471] Billard C., Merhi F., Bauvois B. (2013). Mechanistic insights into the antileukemic activity of hyperforin. Curr. Cancer Drug Targets.

[472] Menegazzi M., Masiello P., Novelli M. (2020). Anti-Tumor Activity of Hypericum perforatum L. and Hyperforin through Modulation of Inflammatory Signaling, ROS Generation and Proton Dynamics. Antioxidants.

[473] Rothley M., Schmid A., Thiele W., Schacht V., Plaumann D., Gartner M., Yektaoglu A., Bruyère F., Noël A., Giannis A. (2009). Hyperforin and aristoforin inhibit lymphatic endothelial cell proliferation in vitro and suppress tumor-induced lymphangiogenesis in vivo. Int. J. Cancer.

[474] Martínez-Poveda B., Verotta L., Bombardelli E., Quesada A.R., Medina M.A. (2010). Tetrahydrohyperforin and octahydrohyperforin are two new potent inhibitors of angiogenesis. PLoS ONE.

[475] Traeger A., Voelker S., Shkodra-Pula B., Kretzer C., Schubert S., Gottschaldt M., Schubert U.S., Werz O. (2020). Improved Bioactivity of the Natural Product 5-Lipoxygenase Inhibitor Hyperforin by Encapsulation into Polymeric Nanoparticles. Mol. Pharm..

[476] Kohlschütter J., Michelfelder S., Trepel M. (2008). Drug delivery in acute myeloid leukemia. Expert Opin. Drug Deliv..

[477] Wu X., Wang F., Yang X., Gong Y., Niu T., Chu B., Qu Y., Qian Z. (2024). Advances in Drug Delivery Systems for the Treatment of Acute Myeloid Leukemia. Small.

[478] Moukalled N., Abou Dalle I., El Cheikh J., Ye Y., Malarad F., Mohty M., Bazarbachi A. (2024). The emerging role of melflufen and peptide-conjugates in multiple myeloma. Curr. Opin. Oncol..

[479] Jia W., Zhou L., Li L., Zhou P., Shen Z. (2023). Nano-Based Drug Delivery of Polyphenolic Compounds for Cancer Treatment: Progress, Opportunities, and Challenges. Pharmaceuticals.

[480] Baiomy R.F.E. (2024). Quercetin nanoparticles as a therapeutic approach: Pharmacological actions and potential applications in therapy. Biotechnologia.

[481] Kazmi I., Al-Abbasi F.A., Afzal M., Altayb H.N., Nadeem M.S., Gupta G. (2021). Formulation and Evaluation of Kaempferol Loaded Nanoparticles against Experimentally Induced Hepatocellular Carcinoma: In Vitro and In Vivo Studies. Pharmaceutics.

[482] Pandey P., Khan F., Alshammari N., Saeed A., Aqil F., Saeed M. (2023). Updates on the anticancer potential of garlic organosulfur compounds and their nanoformulations: Plant therapeutics in cancer management. Front. Pharmacol..

[483] Liu J., Li X., Li Y., Gong Q., Luo K. (2025). Metformin-based nanomedicines for reprogramming tumor immune microenvironment. Theranostics.

[484] Al Jayoush A.R., Haider M., Khan S.A., Hussain Z. (2025). Hyaluronic acid-functionalized nanomedicines for CD44-receptors-mediated targeted cancer therapy: A review of selective targetability and biodistribution to tumor microenvironment. Int. J. Biol. Macromol..

[485] Serini S., Trombino S., Curcio F., Sole R., Cassano R., Calviello G. (2023). Hyaluronic Acid-Mediated Phenolic Compound Nanodelivery for Cancer Therapy. Pharmaceutics.

[486] Zhong Y., Meng F., Deng C., Mao X., Zhong Z. (2017). Targeted inhibition of human hematological cancers in vivo by doxorubicin encapsulated in smart lipoic acid-crosslinked hyaluronic acid nanoparticles. Drug Deliv..

[487] Lagadinou E.D., Sach A., Callahan K., Rossi R.M., Neering S.J., Minhajuddin M., Ashton J.M., Pei S., Grose V., O’Dwyer K.M. (2013). BCL-2 inhibition targets oxidative phosphorylation and selectively eradicates quiescent human leukemia stem cells. Cell Stem Cell.

[488] de Beauchamp L., Himonas E., Helgason G.V. (2022). Mitochondrial metabolism as a potential therapeutic target in myeloid leukaemia. Leukemia.

[489] Addanki S., Kim L., Stevens A. (2025). Understanding and Targeting Metabolic Vulnerabilities in Acute Myeloid Leukemia: An Updated Comprehensive Review. Cancers.

[490] Korbecki J., Bosiacki M., Kupnicka P., Barczak K., Chlubek D., Baranowska-Bosiacka I. (2024). CXCR4 as a therapeutic target in acute myeloid leukemia. Leukemia.

[491] Ogana H.A., Hurwitz S., Wei N., Lee E., Morris K., Parikh K., Kim Y.M. (2024). Targeting integrins in drug-resistant acute myeloid leukaemia. Br. J. Pharmacol..

[492] Farber M., Chen Y., Arnold L., Möllmann M., Boog-Whiteside E., Lin Y.A., Reinhardt H.C., Dührsen U., Hanoun M. (2021). Targeting CD38 in acute myeloid leukemia interferes with leukemia trafficking and induces phagocytosis. Sci. Rep..

[493] Matthews A.H., Pratz K.W., Carroll M.P. (2022). Targeting Menin and CD47 to Address Unmet Needs in Acute Myeloid Leukemia. Cancers.

[494] Pelosi E., Castelli G., Testa U. (2023). CD123 a Therapeutic Target for Acute Myeloid Leukemia and Blastic Plasmocytoid Dendritic Neoplasm. Int. J. Mol. Sci..

[495] Majeti R., Chao M.P., Alizadeh A.A., Pang W.W., Jaiswal S., Gibbs K.D., van Rooijen N., Weissman I.L. (2009). CD47 is an adverse prognostic factor and therapeutic antibody target on human acute myeloid leukemia stem cells. Cell.

[496] Ali M.A., Kaleem N., Ali A., Khan N., Khaliq M., Arif N., Almarhoon Z.M., Habtemariam S., Setzer W.N., Calina D. (2025). Pterostilbene as a Multifaceted Anticancer Agent: Molecular Mechanisms, Therapeutic Potential and Future Directions. Med. Oncol..

[497] Ruan Q., Ruan J., Zhang W., Qian F., Yu Z. (2018). Targeting NAD(+) degradation: The therapeutic potential of flavonoids for Alzheimer’s disease and cognitive frailty. Pharmacol. Res..

[498] Escande C., Nin V., Price N.L., Capellini V., Gomes A.P., Barbosa M.T., O’Neil L., White T.A., Sinclair D.A., Chini E.N. (2013). Flavonoid apigenin is an inhibitor of the NAD+ ase CD38: Implications for cellular NAD+ metabolism, protein acetylation, and treatment of metabolic syndrome. Diabetes.

[499] Liu J., He X., Zhen P., Zhou S., Li X. (2016). [Inflammatory cytokines and oxidative stress markers in the inhibition of osteoarthritis by curcumin]. Zhejiang Da Xue Xue Bao Yi Xue Ban.

[500] Tang C., Wang H., Guo L., Cui Y., Zou C., Hu J., Zhang H., Yang G., Zhou W. (2025). Multifunctional Nanomedicine for Targeted Atherosclerosis Therapy: Activating Plaque Clearance Cascade and Suppressing Inflammation. ACS Nano.

[501] Biganeh H., Dizaji S.M., Taghipour Y.D., Murtaza G., Rahimi R. (2023). Nanoformulations of Plant-Derived Compounds as Emerging Therapeutic Approach for Colorectal Cancer. Curr. Drug Deliv..

[502] Rashidi A., Uy G.L. (2015). Targeting the microenvironment in acute myeloid leukemia. Curr. Hematol. Malig. Rep..

[503] Restelli C., Ruella M., Paruzzo L., Tarella C., Pelicci P.G., Colombo E. (2024). Recent Advances in Immune-Based Therapies for Acute Myeloid Leukemia. Blood Cancer Discov..

[504] Dykes K.C., Ball E.D. (2025). A review of antibody-based immunotherapy clinical trials for adult acute myeloid leukemia (AML): Monoclonal antibodies (mAbs) and beyond. Expert Opin. Biol. Ther..

[505] Halik A., Arends C.M., Bullinger L., Damm F., Frick M. (2022). Refining AML Treatment: The Role of Genetics in Response and Resistance Evaluation to New Agents. Cancers.

[506] Mazzeo P., Penir S.M., Shumilov E., Wolf S., Häupl B., Markus K., Shirneshan K., Rittscher K., Brzuszkiewicz E., Aydilek E. (2025). Clonal evolution and apoptosis resistance in myelodysplastic neoplasms and acute myeloid leukemia under treatment: Insights from integrative longitudinal profiling. Leukemia.

[507] Long N.A., Golla U., Sharma A., Claxton D.F. (2022). Acute Myeloid Leukemia Stem Cells: Origin, Characteristics, and Clinical Implications. Stem Cell Rev. Rep..

[508] Niu J., Peng D., Liu L. (2022). Drug Resistance Mechanisms of Acute Myeloid Leukemia Stem Cells. Front. Oncol..

